# Phytochemical analysis and *in*-*vitro* anti-African swine fever virus activity of extracts and fractions of *Ancistrocladus uncinatus*, Hutch and Dalziel (Ancistrocladaceae)

**DOI:** 10.1186/1746-6148-9-120

**Published:** 2013-06-19

**Authors:** Folorunso O Fasina, Oyinlola O Olaokun, Olusola O Oladipo, Margaret M Fasina, Adesoji A Makinde, Livio Heath, Armanda DS Bastos

**Affiliations:** 1 Department of Production Animal Studies, Faculty of Veterinary Science, University of Pretoria, Onderstepoort 0110, South Africa; 2 Mammal Research Institute, Department of Zoology and Entomology, University of Pretoria, Hatfield, South Africa; 3 Phytomedicine Programme, Department of Paraclinical Science, Faculty of Veterinary Science, University of Pretoria, Onderstepoort 0110, South Africa; 4 National Veterinary Research Institute, Vom, Plateau State, Nigeria; 5 Maximum Farms, P. O. Box 23, Vom Post Office, Plateau State, Nigeria; 6 Transboundary Animal Disease Programme, ARC-Onderstepoort Veterinary Institute, Onderstepoort, South Africa

**Keywords:** Ancistrocladus uncinatus, African swine fever virus, Antiviral

## Abstract

**Background:**

African swine fever (ASF), a highly contagious fatal acute haemorrhagic viral disease of pigs currently has no treatment or vaccination protocol and it threatens the pig industry worldwide. Recent outbreaks were managed by farmers with ethnoveterinary preparations with various claims of effectiveness.

**Results:**

We identified 35 compounds using GC-MS protocol and ASF virus (NIG 99) was significantly reduced by some extracts and fractions of the plant. However, the plant was poorly extracted by water and cytotoxicity was found to be a major problem with the use of the plant since its extracts also reduced the primary cells used in the assay.

**Conclusion:**

It is confirmed that the plant has antiviral potentials against ASF virus and farmers’ claims seem to have certain degree of veracity, but finding the best means of exploring the potential of the plant while reducing its cytotoxic effect *in*-*vitro* and *in*-*vivo* will be necessary.

## Background

African swine fever (ASF), a highly contagious fatal acute haemorrhagic viral disease of pigs results in major economic losses and has substantial food security implications. The disease continues to devastate animal resources (pigs) in parts of Sub-Saharan Africa and other infected regions of the world [1-3]. Though studies are on-going with regards to the preventative actions and immunology of ASF virus (ASFV), to date, little success has been made with regards to the development of preventive vaccine targeting the ASF virus or an effective treatment [4]. This is due to the complex nature of the virus, the acute fatality associated with it and the lack of deep understanding of the immune response in ASF infection [5].

Currently, in the event of an outbreak and its possible spread within pig herds, the standard practice remains the zoning, culling of the herd (stamping out) and payment of compensation to prevent epizootics [1], However, in most African countries, the lack of subsidies for animal agriculture and poor implementation of compensation policy has negatively impacted prompt reporting and weakened transboundary animal disease control. Alternative and complementary therapies have instead been explored by resource-poor small-scale pig farmers in West Africa, in an attempt to save their stock in an outbreak situation. This has included unorthodox methods, including the use of plants and other ethnoveterinary preparations, with widely ranging claims of effectiveness.

Medicinal plants have been used as remedies for centuries and numerous ethnoveterinary assessments of Nigerian/West African plants have been undertaken to evaluate their effectiveness [6-8]. The potency of plant products used as antiviral agents are well-described [9,10], and many plants have been used by farmers to ‘manage’ ASF. However, there are limited peer-reviewed records of plants in general, and none specifically from West Africa with evaluated activity against the ASF virus [11-13]. One such plant fed to pigs has had unconfirmed reports of reducing morbidity and mortality is *Ancistrocladus uncinatus*; and there have even been claims of complete freedom from illness following oral administration of *Ancistrocladus uncinatus* preparations. This Liana plant species was previously described by Hutch and Dalziel in South-Eastern Nigeria as reported by Cheek [14] and a related plant has recently been described by Thomas and Gereau [15]. While the geographical extent of *A*. *uncinatus* has been broadly described, *A*. *korupensis* is specifically found in the tropical swamp of the Korup National Park in Cameroun and adjoining Cross River National Park in Nigeria. It has a low population density, with approximately 13 plants per hectare [16].

Anti-malarial and anti-HIV properties of the Ancistrocladus Liana plant have been reported [17-20]. In particular, certain naphthylisoquinoline alkaloids (including korundamine, yaoundamine, korupensamine have been shown to have a wide range of anti-HIV, antimalarial, fungicidal, larvicidal and moluscicidal biological activities [18-20]. Michellamine B, another alkaloid found in the plant, has demonstrated anti-HIV activity through inhibition of viral reproduction, syncitium formation, enzymatic and cell killing activities [19,21].

Although most viral life cycles consist of the following stages: attachment to the host cell, release of viral gene and possibly enzymes into the host cells, replication of viral components using the host systems, assembly of viral components into complete viral particles and the release of viral particles to infect new cells [22]

Various antivirals have been developed and their actions range from identifying and disabling or interfering with functional viral proteins and critical enzymes for the virus, insertion of stop codons into the virus genetic structures, interfering with the ability of the virus to infilterate the target cells by binding the receptors and attachment molecules, by blocking the binding site of the virus or by the use of uncoating inhibitors amongst others [23-25].

The antivirals for DNA viruses of which ASF is one, can operate in one of the following ways:

• DNA synthesis inhibitors which can be *Thymidine kinase* (*TK*) activated or not. The *TK* activated antivirals are categorised as Purine analogues (e.g. Guaning, Ganciclovir, Penclovir) or Pyrimidine analogue (e.g. uridine, Trifluridine, Thymine, Cytosine etc.). The non-*TK* activated antivirals include the Foscarnet and others. In addition, there are other antivirals that are not classified in this group including the Tromantadine, Docosanol and early protein affecting drug like Fomivirsen [23,25].

• Viral assembly disruption for example Rifampicin or by inhibiting mRNA and protein synthesis, e.g. Methisazone [26,27].

• Nucleoside analogue (e.g. Entecavir, Lamivudine), nucleotide analogues (e.g. Adefovir, Tenofovir), Nucleic acid inhibitors (e.g. Cidofovir), interferon-cytokine stimulation (e.g. Interferon alpha 2b,) or with unknown effect (e.g. Ribavirin, Moroxydine) [28,29].

Specifically, with regards to the ASFV, certain antivirals have been assessed against the virus including Chloroquine, which produced a time-dependent, fully-reversible inhibition of both cytopathic effects and the production of African swine fever virus (ASFV) in Vero cells, but does not have any direct effect on the virus nor on viral adsorption and internalization [30]. Cholesterol reducing/removing drugs like Nystatin and Methyl-β-Cyclodextrin have been proved to affect ASFV fusion and subsequent replication since Cholesterol in the target membrane is needed for these functions to be completed [31,32]. These drugs directly impact on ASFV entry and infection of cells.

Other drugs include Fluoroquinones a group of drugs that has severely reduced the cytopathic effect of ASFV infected Vero cells from early phase of infection and prevented the detection of ASFV genome 7-days post treatment. The drug also caused altered viral protein possibly because of the putative ASFV-topoisomerase II enzyme which was targeted and had its activity modified [33]. Lauryl gallate has been tested and confirmed to strongly inhibit African swine fever virus at non-toxic concentration. ASFV production in Vero cells was completely prevented by the addition of the drug 1 hour before virus adsorption; however in cells that were 5–8 hours post infection, the drug had no effect. This same drug has been shown to prevent both cellular and viral DNA synthesis and viral transcription amongst other effect [34].

A dose-dependent viral-inhibition of African swine fever virus has been reported in in-vitro assessment of aqueous extracts of *Pophyridium cruentum*, *Chlorella autotrophica* and *Ellipsoidon sp*. possibly due to sulphated polysaccharides [35]; and both resveratrol and oxyresveratrol also worked in dose-dependent manners in an in-vitro experiment causing a 98-100% reduction in virus multiplication and viral titres; and inhibited viral DNA replication. Though early viral protein synthesis was observed in this experiment, late viral protein synthesis and viral factory formation were blocked [13].

In addition, Valproic acid has been reported to cause a significant reduction in the yield of ASFV and other enveloped viruses possibly through its effect on viral maturation and envelope formation in enveloped viruses [36] and other such drugs that have been tested against ASFV include but not limited to Chlorpromazine, Dynamin, Clathrin and Cholera toxin [32,37].

In this study, we used a molecular biology approach to evaluate the antiviral potentials of the plant *A*. *uncinatus* in an *in*-*vitro* model of infection. Primary bone marrow cells were infected with ASFV, treated with crude extracts and fractions of *A*. *uncinatus* and their effect on the virus were evaluated by real-time and conventional PCR.

## Results

### Phytochemical screening of leaves, stem and roots of Ancistrocladus uncinatus

Leaf, root and stem portions of the pulverised plant revealed the presence of alkaloids, cardiac glycosides and steroids; saponins and flavonoids were only recovered from the leaves while tannins were recovered from the stem (Table 1). None of the plant portion contained anthraquinones.

**Table 1:** **Secondary metabolites found in the different stem barks, leaves and roots of the *A. uncinatus***

**Sample**	**Saponin**	**Alkaloids**	**Cardiac glycosides**	**Steroids**	**Tannin**	**Anthraquinones**	**Flavonoids**
Leaves	+	+	+	+	-	-	+
Roots	-	+	+	+	-	-	-
Stem	-	+	+	+	+	-	-

### Determination of chemical compounds from A. uncinatus

The chemical compounds present in the different portions of the plants identified by Gas chromatography–mass spectrometry (GC-MS) are summarised in Table 2. A total of 35 chemical compounds were identified with N-Formylkorupensamin B being the most abundant in the plant but concentrated more in the stem and leaves. Certain compounds or their derivatives were present in all parts of the plant while other compounds were recovered only from certain parts of the plant (see Table 2, and Additional files 1, 2 and 3).

**Table 2:** **Compound expressed from the stem barks, leaves and roots of *Ancistrocladus uncinatus***

	Compounds (source)*	**Molecular weight**	**Retention time**	**Retention index**
1.	n-Hexadecanoic acid (S, L)	256	25.43	1968
2.	7-Hexadecenoic acid (S)	268	27.24	1886
3.	9-Hexadecenoic acid (S, L, R)	254	27.76	1976
4.	Octadecanoic acid (S, L)	284	28.06	2167
5.	1-Butanamine (S)	155	29.58	1103
6.	1,9-Nonanediol (S, R)	160	31.63	1401
7.	Hexadecanoic acid (S, L, R)	330	31.94	2498
8.	3-Bromooctane (S)	192	32.37	1049
9.	1,3-Tetradecenal (S)	210	33.88	1591
10.	2-Quinolinecarboxylic acid (S)	421	38.31	2310
11.	N,N’-Bis (p-Methoxybenxylidine) benzidine (S, L, R)	420	38.50	3749
12.	N-Formylkorupensamin b (S, L)	407	39.28	3792
13.	4-Acetoxy-6′,7-dimethyl-5′,8′-dimethoxy-1,2′-binaphthalene-1′,4′,5,8-tetrone (S)	460	41.97	3926
14.	4-Pentadecyne (S)	242	42.69	1755
15.	Stigmasterol,22,23-dihydro- (S)	414	44.14	2731
16.	Bicyclo[3.1.0]hexan-3-ol (S)	154	45.35	1079
17.	(Z)6,(Z)9-Pentadecadien-1-ol (L)	224	31.56	1771
18.	13-Oxabicyclo[10.1.0]tridecane (L)	182	33.88	1450
19.	7-Tetradecenal (L)	210	31.63	1609
20.	Squalene (L, R)	410	35.58	2914
21.	Silane (L)	442	35.78	2647
22.	Beta-Tocopherol (L, R)	416	38.30	3036
23.	1H,3H-Furo[3,4-c]furan (L, R)	446	39.28	3243
24.	Vitamin E (dl-alpha-Tocopherol) (L, R)	430	39.96	3149
25.	Gamma-Sitosterol (L)	414	44.14	2731
26.	3,6-Octadien-1-ol,3,7-dimethyl-(Z)- (L)	154	45.37	1228
27.	Cyclopentanol,3-methyl-2-(2-pentenyl)- (L)	168	46.09	1315
28.	Decane, 1-chloro- Decyl Chloride (R)	176	44.13	1240
29.	3-Octadecyne (R)	250	45.34	1828
30.	2-Isopropyl-5-methylcyclohexymethanol (R)	170	42.68	1280
31.	9-Octadecenoic acid (R)	296	27.24	2085
32.	1-Fluorononane (R)	146	31.93	889
33.	3,8-Dibenzoyl-1-nitro-3,6,8-triazabicyclo[4.3.1]decane (R)	394	32.83	3353
34.	Oxalic acid (R)	368	28.68	2606
35.	2,2,3,3,4,4,-Hexamethyltetrahydrofuran (R)	156	29.58	992

*Source of chemical compound as determined by gas chromatograph-mass spectrometry. S = stem, L = leaves and R = combination of residual leaves, stem and root. A total of 35 compound and its derivatives were clearly isolated from the *A*. *uncinatus* stem, leaves and root.

See Additional files 1, 2 and 3 for details of the identified compounds.

### Phytochemical constituents expressed on silica gel

**Figure 1:**
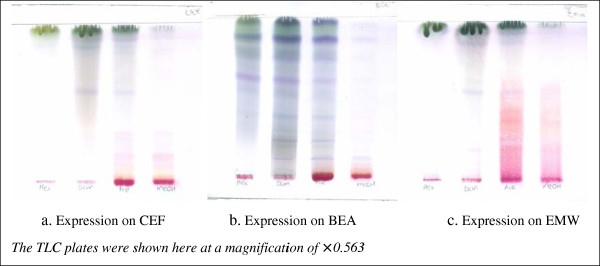
**Expression fraction of Acetone extracts of A. uncinatus on TLC plates using three different expression methods. a-c** are chromatograms of Hexane (Hex), Di-Chloro Methane (DCM), Acetone (Ace) and methanolic (MeOH) extracts respectively. *CEF = Chloroform-Ethyl acetate-Formic acid; BEA = Benzene-Ethanol-Ammonia (BEA), and EMW = Ethyl acetate-methanol–water.*

Thin-layer-chromatography of fractions of Acetone extracts in Ethyl acetate-methanol–water (EMW), Benzene-Ethanol-Ammonia (BEA), and Chloroform-Ethyl acetate-Formic acid (CEF) revealed that several active principles exist in *A*. *uncinatus* and that these were best expressed using BEA followed by CEF and then EMW. It appears that the dominant principles in the plant were non-polar/basic compounds but other chemicals with varying polarities were also observed. The retention factors (R_*f*_) of the 10 clearly identified compounds in BEA were: 0.125; 0.175; 0.225; 0.263; 0.375; 0.538; 0.763; 0.850; 0.888 and 0.925 (see Figure 1b).

### Cell viability and cytotoxicity assays

**Figure 2:**
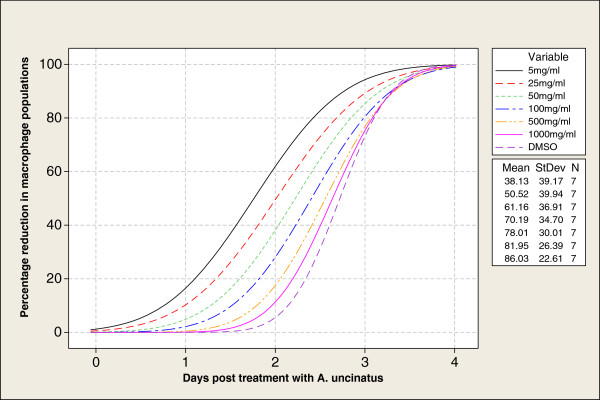
Dose-Effect Curves and Cytotoxicity patterns of A. uncinatus.

PBMC were confirmed viable since the cell culture media gradually used up the phenol red in the medium and changed the colour from orange to pale yellow over a period of 7 days. The plates inoculated with ASF NIG/99 virus showed distinct rosette formations around the macrophages, an indication that the macrophages were infected and haemadsorped with the pig red blood cells in the medium. There was no visible reduction in cell population when compared with cells inoculated for diagnostic purposes and no rosette formations were visible in the plates inoculated with placebo (wash buffer only). Complete or partial CPE was observed with concentrations of extract ≥5 mg/ml and for the pure extract diluent (DMSO), however a 1:1000 dilution of the diluent was non cytotoxic to the PBMC; there was no apparent reduction in the macrophages population and rosette formations developed normally compared to the cells without the diluent (see Figure 2).

### Antiviral assay of extract of A. uncinatus and its fractions on African swine fever virus

Cells in the PBMC grew normally until approximately 96 hours post-infection following which some reductions in rosette formations were observed. However, after 120 hours, marked reduction in the population of Macrophages and CPE were observed indicating cell deaths. Cell culture plates were read approximately 108–109 hours post-treatment. Noticeable reductions in the quantity of rosette formations were observed in wells treated with acetone, dichloromethane and methanol extracts and also in wells treated with hexane, methanol and ethyl acetate fractions of acetone extract (Table 3). Hexane and chloroform extracts and chloroform fraction of Acetone extract showed minimal reduction in the number of rosettes observed and counted, indicating weak activities against ASFV (Table 3). The IC_50_ of the Acetone extract was determined to be 17 μg.

**Table 3:** **Observation of activities of *Ancistrocladus uncinatus *extracts and fractions on haemadsorbing African swine fever virus *in-vitro *5-days post infection**

	**1**	**2**	**3**	**4**	**5**	**6**	**7**	**8**	**9**	**10**	**11**	**12**
**Mg/ml**	**ASF NIG/99 (+)**	**Acetone (C)**	**Hexane(C)**	**DCM (C)**	**Chloroform (C)**	**Methanol (C)**	**Hexane (F)**	**Methanol (F)**	**Ethyl acetate (F)**	**Chloroform (F)**	**Blank**	**Placebo (−)**
1	+++	-	+	-	++	-	-	-	-	+		-
0.5	+++	-	++	-	++	-	-	-	-	+		-
0.25	+++	-	++	-	++	-	-	-	-	++		-
0.125	+++	-	+++	-	+++	-	-	-	+	++		-
0.0625	+++	-	+++	+	+++	-	-	+	+	+++		-
0.03125	+++	+	+++	++	+++	++	+	++	++	+++		-
0.015625	+++	++	+++	++	+++	++	++	++	++	+++		-
0.0078125	+++	+++	+++	+++	+++	+++	+++	+++	+++	+++		-

+++ = ≥50 rosettes were observed and counted in the well; ++ = 10–50 rosettes were observed and counted; + = ≤10 rosettes were observed and counted. - = no rosette was observed. C = crude extract and F = fraction of extract. N.B. Rosette formation is an indication of virus infection of the macrophage cells in the PBMC medium.

### Conventional PCR and real-time PCR (QPCR) assays

**Figure 3:**
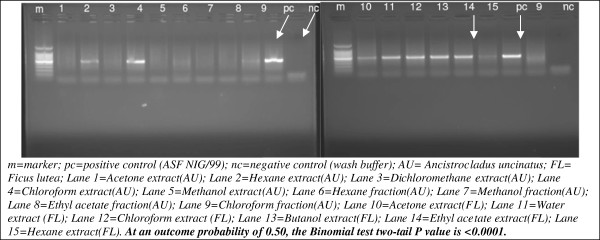
Agarose gel electrophoresis of conventional PCR assay used to asses the effect of A. uncinatus on ASF virus.

The PCR results confirmed the observed reduction in rosette formation associated with reduced activities of ASFV *in*-*vitro* in the presence of *A*. *uncinatus* extracts or fractions. It will appear that Acetone, Dichloromethane, Methanol extracts and hexane, methanol and ethyl acetate fractions of Acetone extract were effective against the ASFV as no 478 bp product was observed (Figure 3, Lanes 1–9). However, Hexane extract and Chloroform fraction displayed partial activities while Chloroform extract showed no activity against ASFV. The *Ficus lutea* plant control showed no activity against ASFV (Figure 3, Lane 10–15). Both the positive and negative controls passed the internal quality control test required to accept the results (see Figure 3).

No detectable level of ASF viral genome was observed and quantitated from the QPCR since the fluorescent measurement was not above the background signal for any of the tested samples and no sigmoid-shaped curve was observed.

### Re-infectivity assay of extract of A. uncinatus

None of the cultures containing plant extracts (acetone, dichloromethane and methanol) and its fractions (hexane, methanol and ethyl acetate fractions) showed rosette formation 96 and 120 hours post-inoculation indicating the lack of infectious virus in the inoculum. However, the positive control wells (ASF NIG/99 virus) displayed characteristic growth patterns and rosette formations that were comparable to the expected standards. No growth was observed in the negative controls (wash buffer). Observed cytotoxicity was also similar to what was previously reported in the cytotoxicity assay.

## Discussion

Certain important plant metabolites were found in abundance in the analysed plant including the cardiac glycosides, alkaloids and steroids. Cardiac glycoside has been employed in the treatment of congestive heart failure and cardiac arrhythmia. The ASF virus affects many visceral organs including the heart and it has been suggested previously that death following infection like ASFV may be due to heart failure [38]. It is possible that this plant positively influences cardiac outputs by increasing the force of contraction through its effect on the sodium-potassium pumps in the cell membrane [39]. In addition, alkaloids and steroids from this plant may have various pharmacological effects and may minimize the effect of the virus on the pig cells during *in*-*vivo* infection. Additional research is required to determine the particular metabolites or combinations of metabolites that are responsible for the therapeutic claims ascribed to this plant by the farmers. Galindo and colleagues [13] and Fabregas et al., [35] has confirmed that chemical compounds from natural plants performed as effectively as those from synthetic sources.

Gas chromatography has been known to separate large numbers of compounds in a single analysis and in combination with mass spectrometry (GC-MS) usually results in a highly selective and sensitive method of chemical compound analysis in plants [40]. In this analysis, we used the method of GC-MS to identify at least 35 compounds from the plant *A*. *uncinatus*. These compounds are consistent with those presented in previous reports [17,18,21]. The range of activities of these compounds extends from anti-tumour, febrifugal, virucidal, anti-insulin, antibacterial, prostate treatment, vaccine constituents to anti-hypercholesterolemic [40-44].

While activities of this plant against HIV and certain other human viruses have been evaluated [17,19], this is the first report of its use against animal viruses specifically. Though, the particular compound or interaction of compounds that was responsible for this anti-ASF activity in the crude extracts and fractions used in this study are not yet known, the *in*-*vitro* results support the claim made by farmers of effectiveness of this plant in the management of ASFV. Based on the re-infectivity assay, some extracts and certain fractions of the plant have good virucidal activities which can be positively explored further. The cell culture system results were further supported by conventional PCR and QPCR. Further testing of each identified compound for individual and combined ranges of biological activity against ASFV is imperative.

It will also be important to carefully examine the cause of cytotoxicity in this plant and devise ways of eliminating or reducing this effect in view of the substantial therapeutic potential of this plant. Though the extracts and fractions significantly reduced ASFV titres, they also did significant damage and caused major reduction in PBMC populations in the culture. Laird and Lisinge [45] and Laird [46] have previously reported on the toxicity associated with the *A korupensis* and this effect appeared to be cumulative in this study using *A*. *uncinatus*. Similar report has been associated with increasing dose of other drugs tested against the virus [13,33,34]. The antiviral activities of this plant are comparable to those of Lauryl gallate and mulberry twigs which caused significantly reduced viral titres and inhibition of viral DNA replication at non-toxic doses [13,34].

It is possible that there is continuous intra-cytoplasmic absorption of *A*. *uncinatus* by the PBMC and the post-96 hour levels of absorption were incompatible with the survival of the macrophages due to this increasing toxicity [44-46]. Thus, since the toxic dose level appears to be quite close to the anticipated effective antiviral dose against ASFV, the plant likely has a narrow therapeutic index in the field. It is possible that some of the pigs that initially recovered following treatment with *A*. *uncinatus* but later died may have succumbed to the toxic effect of the plant. While this plant holds potential for the treatment of certain viral infections in pigs, including ASF, its cytotoxicity remains a concern that will require extensive *in*-*vivo* assessment of acute and chronic toxicity in live animals in order to validate the effectiveness and therapeutic index of *A*. *uncinatus* in the management of ASF in pigs.

The water extraction method poorly expressed the active plant compounds and most of the expressed compounds are basic to neutral. However, since water is an important medium for drug administration, it will be important to conduct additional studies to validate methods by which water may be used in the administration of compounds and extracts from *A*. *uncinatus*.

## Conclusion

In conclusion, the farmers’ claims of the effectiveness of the use of *Ancistrocladus uncinatus* in the management of African swine fever seem to have a degree of empirical support. Our experiment has provided evidence and confirmed that the extracts and fractions of extract from the plant have antiviral/virucidal activities against ASF virus. It significantly reduced the viral titres in the PCR assay and prevented virus replication to undetectable level in the QPCR experiment as well as terminated the infectiousness of the virus *in*-*vitro*. However, the cytotoxic effect of the plant will need to be overcome in order to reduce the negative effect of the plant while still harnessing its therapeutic potential. Further research on the antiviral compounds and effect of the plant holds potential for uncovering a novel antiviral compound and should be explored further for this and other animal viruses for which treatment options are either limited or non-existent.

## Methods

### Pre-screening of ethnoveterinary products used for ASF management, plant collection and identification

Oral interviews were conducted with selected pig farmers from different zones of Nigeria on the management of African swine fever using ethnoveterinary preparations during the past outbreaks of the disease, as part of the national swine disease surveillance programme. Many ethnoveterinary preparations were reported as being used which were screened against published resources to determine whether any preliminary antiviral potential of the plants on the list had been confirmed previously [6-8]. *Ancistrocladus uncinatus* was selected on this basis, for further assessment against the African swine fever virus.

The plant was identified at the Cross River National Park, and sample specimens were collected for the preparation of a herbarium. Authentication of the plant was carried out by Mal. U. S. Gallah at the Herbarium of the Ahmadu Bello University, Zaria, Nigeria using the prescribed standards [14,15], and deposited in the herbarium with the Voucher number 109413. The plant were air-dried in the laboratory and separated into portions of leaves, stems and roots. Each portion was pulverised using Jika-Werke M20 blender (Jika-Werke, Staufen, Germany) and stored in airtight cellophane bags at +4°C until used.

### Phytochemical screening of leaves, stem and roots of Ancistrocladus uncinatus

Portions of the pulverised plant were screened for phytochemical content and for certain secondary metabolites including alkaloids, flavonoids, cardiac glycosides, saponins, tannins, anthraquinones, triterpenes and steroids using standard methods [8,47-49].

### Determination of chemical compounds from A. uncinatus

The chemical compounds present in the plant were determined using the analysis of gas chromatography–mass spectrometry (GC-MS) and the modified method of Ivanov and Sandell [50]. Briefly described, 2 g portions of leaves, stems and whole plant (stems, leaves and roots) of *A*. *uncinatus* were each extracted with petroleum ether and injected into column of the Shimadzu Gas Chromatograph-Mass Spectrometer (GC-MS) QP 2010 PLUS (Shimadzu, Japan) and its software programme for analysis. Oven temperature was 60°C and injection temperature was 250°C, with a linear velocity of 46.3 cm/sec, a capillary column flow rate of 1.61 mL/min and a pressure of 100.2 kPa. For the GC programme, the Ion source was set at 200°C and the interface temperature of 250°C had a threshold of 3000 [51]. The MS analysis was done based on comparative retention times, mass and peaks of the chemical compounds using the NIST05.LIB as the reference database [52]. This library enables the facilitation of comparison of generated spectra with the standards using Probability Based Matching algorithms [52].

The (GC-MS) QP 2010 PLUS had also been pre-fitted with a set of automated internal validity programmes for the analysis, including the adjustment of retention time function, scan measurement, quick and accurate compound identification from chromatogram, search based on mass spectra similarity and other quality assurance-quality control functions [53].

### Extractions and fractions from A. uncinatus

Individual distilled water, Acetone (Ace), Methanol (MeOH), Hexane (Hex), Chloroform (Chloro) and DiChloroMethane (DCM) extracts of each of the plant parts were made using previously described methods [54]. Briefly, 10 g of the finely ground plant material was soaked in 100 ml of each of the solvents in an Erlenmeyer flasks. The contents were shaken on a Labotec shaker M202 (Labotec Pty, South Africa) for 30 minutes after which each extract was centrifuged at ≈ 2150 rpm (4000 g, 12 cm radial distance) for 5 minutes in a Rotofix 32A centrifuge (Hettich Zentrifugen, Tuttlingen, Germany). The supernatants were filtered using a 125mmØ qualitative circles Whatman paper (no. 1) and a glass funnel into pre-weighed glass vials. The whole process was repeated thrice for each extract to exhaustively extract the plant materials and the total volumes of each filtrate were combined and solvent air-dried at room temperature in a fume cupboard. The final products were weighed individually and stored at +4°C until further use.

The different extracts were dissolved in Dimethyl sulfoxide (DMSO) to make a final 100 mg/ml stock solution (ratio of 100 mg of extract to 1 ml DMSO). The quantity of materials recovered, with the exception of the water extraction process, was sufficient to enable further analysis.

### Phytochemical analysis on silica gel

Portions (1 ml) of the acetone extract were dissolved in 9 ml of Hexane, Dichloromethane, Acetone and Methanol to make a 10 mg/ml fractions of each solution. Ten microlitres (10 μ) of each solution was spotted on a pre-labelled aluminium-backed TLC silica plates (TLC Silica Gel 60 F_254_, Merck, Darmstadt, Germany) with a micropipette, 1 ml from the bottom of the plates and thin-layer-chromatography was carried out in Ethyl acetate-methanol–water [40:5.4:5] (EMW; polar/neutral), Benzene-Ethanol-Ammonia [90:10:1] (BEA; non-polar/basic), and Chloroform-Ethyl acetate-Formic acid [5:4:1] (CEF; intermediate polarity/acidic) using the method of Kotze and Eloff [54]. Chromatograms were developed in closed tanks in which the eluent wetted the TLC plates. The final chromatograms were air-dried and sprayed with Vanillin vapour (0.1 g)-Methanol (28 ml)-H_2_SO_4_ (1 ml) solution. The Vanillin-sprayed plates were then heated with dry heat for approximately 3 minutes at 110°C for optimal colour development and detection of the separated compounds.

Based on the expression on BEA, the retention factors of the 10 clearly identified compounds were calculated using the formula:

$$R_{f}=\frac{\mathrm{Distance}\;\mathrm{travelled}\;\mathrm{by}\;\mathrm{substance}}{\mathrm{Distance}\;\mathrm{travelled}\;\mathrm{by}\;\mathrm{solvent}}$$

### African swine fever virus and the primary bone marrow culture

ASF NIG/99 (a haemadsorbing virus responsible for major ASF outbreaks in Nigeria in 1999) was obtained from the virus repository of the Transboundary Animal Disease Programme (TADP) of the ARC-Onderstepoort Veterinary Institute, South Africa. Primary bone marrow culture (PBMC) adjusted to 1 × 10^7^ cells/ml was prepared in the 96-well flat bottom tissue culture plates (Corning Costar®, Sigma Aldrich, Aston Manor, South Africa) according to the Standard Operating Procedures (R&D ASF 04–00) of TADP and incubated at 37°C for 48 hours at 5% CO_2_. The plates were observed under the microscope for growth of macrophages, after which the liquid contents of the plates were discarded 48 hours post preparation and 100 μl of freshly prepared growth medium was dispensed into each well of the plates. The primary cells were then available for virus infection. Additional file 4.

### Cell viability and cytotoxicity assays

To assess for the viability of the PBM cells, consistency of the plates were first checked for colour change (light orange to pale yellow due to active metabolism in the plates). Furthermore, each culture plate was inoculated with 100 μl of ASF NIG/99 virus (7.0 log_10_ HAD_50_/ml.) and the placebo (wash buffer), sealed and incubated in a 5% CO_2_ incubator at 37 C for 48 hours and checked for haemadsorption activity (rosette formation) and cytopathic effect (CPE). Cytotoxicity assay was done by inoculating the PBMC with different concentrations of crude acetone extract of *A*. *uncinatus* (1000 mg/ml, 500 mg/ml, 100 mg/ml, 50 mg/ml, 25 mg/ml, 5 mg/ml and 1 mg/ml), and the diluent (DMSO); and checking daily for decreasing number of macrophages and rosette formations. Based on the quantitated values obtained, a dose-effect graph was plotted for the different concentrations of the acetone extract (see Figure 2).

### Antiviral assays of extract of A. uncinatus and its fractions on African swine fever

#### Cell culture assay system

The modified methods of Vanden Berghe et al. [9] and Ying-Wang et al., [10] were used when carrying out the antiviral assessment of the plant.

Fresh PBMCs were prepared on the 96-well flat bottom tissue culture plates as stated above and infected with 100 μl of the ASF NIG/99 virus (7.0 log_10_ HAD_50_/ml).

One in two (1:2) serial dilutions of the extracts (Acetone, Hexane, DochloroMethane, Methanol and Chloroform) and its fractions (Hexane, Methanol, Ethyl Acetate and Chloroform) were prepared in ordinary 96-well U-bottom plates to deliver 1 mg/ml up to 0.0078 mg/ml in a 50 μl of each dilution. These dilutions were added to rows in the ASF infected plates (see Table 4). *Ficus lutea* extracts were used as plant controls. Only 50 μl of the wash buffer was added to the positive controls and no virus, extract or fraction was added to the negative controls. The plates, prepared in triplicate were sealed, and each of the experiments was performed twice. The plates were incubated in a 5% CO_2_ incubator at 37 C for 48 hours and checked for haemadsorption activity (rosette formation) and CPE. The 50% inhibitory concentration, IC_50_ (a dose that will reduce the activity of the virus by approximately 50%) was calculated by using the formula:

$$\begin{array}{l} \mathrm{EXP}\;(\mathrm{LN}\left(\mathrm{Conc}>50\%\right)-((\mathrm{signal}>50\%-50)/\\ \;(\mathrm{signal}>50\%-\mathrm{signal}<50\%)*\mathrm{LN}\\ \;(\mathrm{conc}>50\%/\mathrm{conc}<50\%))) \end{array}$$

Using an automated excel worksheet developed by Professors Maes and Cos of Antwerp University.

**Table 4:** Set up of the test system for the antiviral assay

	**1**	**2**	**3**	**4**	**5**	**6**	**7**	**8**	**9**	**10**	**11**	**12**
**mg/ml**	**ASF NIG/99 (+)**	**Acetone (C)**	**Hexane(C)**	**DCM (C)**	**Chloroform (C)**	**Methanol (C)**	**Hexane (F)**	**Methanol (F)**	**Ethyl acetate (F)**	**Chloroform (F)**	**Blank**	**Placebo (−)**
1	+	*	*	*	*	*	*	*	*	*		-
0.5	+	*	*	*	*	*	*	*	*	*		-
0.25	+	*	*	*	*	*	*	*	*	*		-
0.125	+	*	*	*	*	*	*	*	*	*		-
0.0625	+	*	*	*	*	*	*	*	*	*		-
0.03125	+	*	*	*	*	*	*	*	*	*		-
0.015625	+	*	*	*	*	*	*	*	*	*		-
0.0078125	+	*	*	*	*	*	*	*	*	*		-

(C) = crude extract; (F) = Fraction; DCM = Dichloro Methane; + = Positive control (ASF NIG/99); * = Crude extract or fractions as stated in the table; - = Placebo/Negative control (wash buffer).

### PCR and real-time PCR

Following a 7-day incubation period, the plates were observed under the microscope and the 1 mg/ml test systems of the *A*. *uncinatus* extracts and their fractions were harvested and assessed by conventional PCR targeting a 478 bp region of the *p72* gene to determine if there was any reduction in viral titres due to the effect of the plant. Briefly, viral DNA was extracted from the harvests using the supplier-prescribed *High Pure PCR Template Preparation Kit* (Roche Diagnostic GmbH, Mannheim, Germany) protocol. A set of forward and reverse primers: (p72U-GGCACAAGTTCGGACATGT [sense] and p72D-TGTAACGCAGCACAG [anti-sense] were used to amplify the C-terminal end of virus protein (VP) 72, as previously described [55]. The resulting products were sized by 1.5% agarose gel electrophoresis against a 100 bp marker (Fermentas).

Real time/quantitative PCR (QPCR) was used to determine the residual quantity of the ASF viral genome that was left in the extract/fraction treated samples or whether viral replication subsists in the presence of extract. Briefly, a set of forward (King-s (5′-CTGCTCATGGTATCAATCTTATCGA-3′) and reverse King-a (5′-GATACCACAAGATCRGCCGT-3′) primers, that amplify a conserved 250 bp region of VP72 were combined with a TaqMan probe (5′-FAM-CCACGGGAGGAATACCAACCCAGTG-TAMRA-3′) that detects the amplified product with the label/reporter at the 5′ end [6-carboxy-fluorescein (FAM) and a quencher at the 3′ end (6-carboxy-tetramethyl-rhodamine (TAMRA)] [56]. The system was optimised at 95°C 3 min; 95°C 10s; 58°C 30s and 45 cycles with a cycle threshold (Ct) value of 32 ± 2. The complete protocol is available at http://asf-referencelab.info/asf/files/SOPs/SOP-ASFPCR22008.pdf.

### Re-infectivity assay

Re-infectivity assay was performed to determine whether the observed effect of the plant on the virus was virucidal or virustatic and to correlate the PCR results with the cell culture; briefly, 100 μl of the recently harvested virus-extracts/fractions as well as the positive (ASF NIG/99 virus) and the negative (wash buffer) controls were filtered using the 0.22 μ filter and inoculated again into freshly prepared PBMC. The culture plates were incubated as described above and microscopically inspected 72, 96 and 120 hours post-infection to determine the residual virus infectivity following exposure to the extracts/fractions.

### Ethical approval

Ethical clearance and approval were obtained as part of the overall approval for the project at the National Veterinary Research Institute, Vom, Nigeria under the Project Code 025060410100000 (*Development of rapid and effective Diagnostic and Control tools for African Swine Fever*). No human or animal subject was used directly in the project.

## Competing interests

We do not have any competing interest that should prevent the publication of this article.

## Authors’ contributions

FOF, LH, ADSB conceived the project and draw up the protocols. MMF identified and prepared the plant for laboratory analysis with OOO+. FOF and OOO carried out the TLC and extraction procedures; OOO+, MMF and FOF carried out the phytochemical screening and GC-MS; FOF did the cell culture and molecular biology works. LH, ADSB and FOF carried out the analyses. All authors contributed to the drafting of the final manuscript and approved it for submission.

## Supplementary Material

Additional file 1 Gas chromatography-mass spectrometry result of the leaf, stem bark and root of Ancistrocladus uncinatus. Click here for file

Additional file 2 Gas chromatography-mass spectrometry result of the leaf of Ancistrocladus uncinatus. Click here for file

Additional file 3 Gas chromatography-mass spectrometry result of the stem bark of Ancistrocladus uncinatus. Click here for file

Additional file 4 Preparation of Primary Bone Marrow Cultures. Click here for file
