# Supplementary material for: Phytochemical analysis and in-vitro anti-African swine fever virus activity of extracts and fractions of Ancistrocladus uncinatus, Hutch and Dalziel (Ancistrocladaceae)
Source: BMC Vet Res. 2013 Jun 19;9:120. doi: 10.1186/1746-6148-9-120 (PMC3694037; doi:10.1186/1746-6148-9-120)

NARICT

FASINA

GCMS-QP2010 PLUS
SHIMADZU,JAPAN

NARICT, ZARIA
GCMS ANALYSIS

SAMPLE - LEAVES + STEM BARK + ROOT

Chromatogram

Chromatogram LEAVES + STEM Sample C:\GCMSsolution\SOLA OLADIPO\SOLA OLADIPO003.qgd

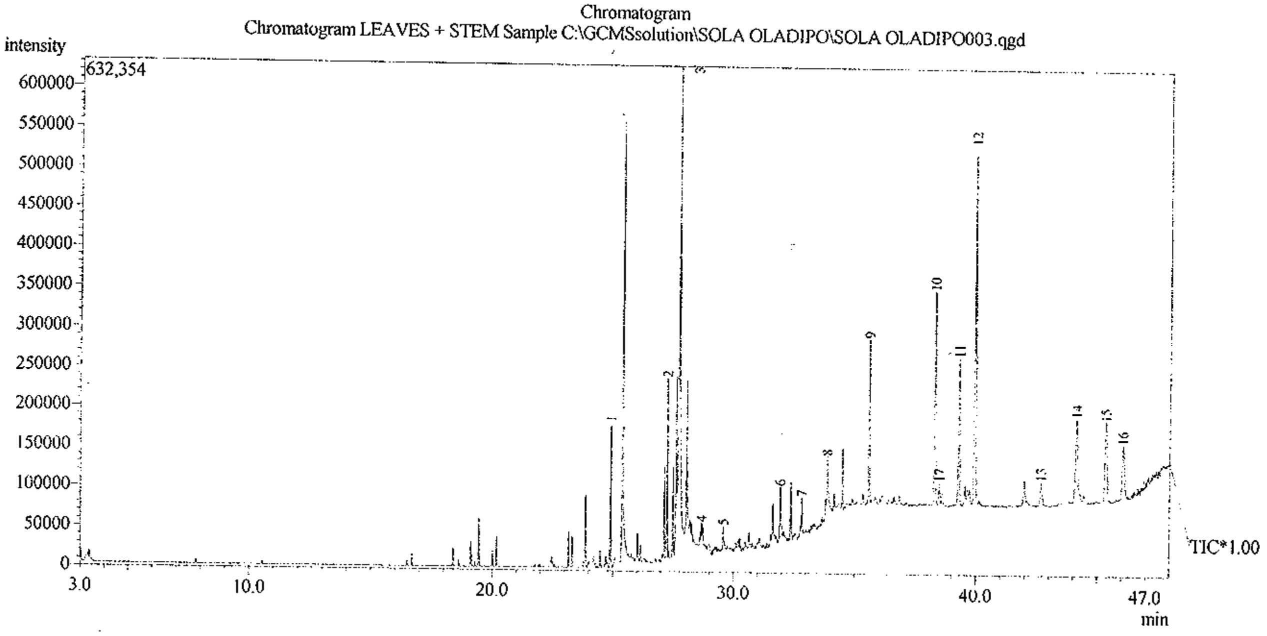

Method

[Comment]

===== Analytical Line 1 =====

[AOC-20i]

# of Rinses with Presolvent	:5
# of Rinses with Solvent(post)	:5
# of Rinses with Sample	:3
Plunger Speed(Suction)	:High
Viscosity Comp. Time	:0.2 sec
Plunger Speed(Injection)	:High
Syringe Insertion Speed	:High
Injection Mode	:Normal
Pumping Times	:5
Inj. Port Dwell Time	:0.3 sec
Terminal Air Gap	:No
Plunger Washing Speed	:High
Washing Volume	:8uL
Syringe Suction Position	:0.0 mm
Syringe Injection Position	:0.0 mm
Use 3 Solvent Vial	:1 vial

[GC-2010]

Column Oven Temp.	:60.0 °C
Injection Temp.	:250.00 °C
Injection Mode	:Split
Flow Control Mode	:Linear Velocity
Pressure	:100.2 kPa
Total Flow	:6.2 mL/min
Column Flow	:1.61 mL/min
Linear Velocity	:46.3 cm/sec
Purge Flow	:3.0 mL/min
Split Ratio	:1.0
High Pressure Injection	:OFF
Carrier Gas Saver	:OFF
Splitter Hold	:OFF

Oven Temp. Program

Rate	Temperature(°C)	Hold Time(min)
-	60.0	3.00
7.00	140.0	0.00
7.00	280.0	15.00

< Ready Check Heat Unit >
Column Oven : Yes
SPL2 : Yes
MS : Yes
< Ready Check Detector(FTD) >
< Ready Check Baseline Drift >
< Ready Check Injection Flow >
SPL2 Carrier : Yes
SPL2 Purge : Yes
< Ready Check APC Flow >
< Ready Check Detector APC Flow >
External Wait :No
Equilibrium Time :3.0 min

[GC Program]

[GCMS-QP2010 Plus]
IonSourceTemp :200.00 °C
Interface Temp. :250.00 °C
Solvent Cut Time :2.50 min
Detector Gain Mode :Relative
Detector Gain :0.00 kV
Threshold :3000

[MS Table]
--Group 1 - Event 1--
Start Time :3.00min
End Time :48.00min
ACQ Mode :Scan
Event Time :0.50sec
Scan Speed :1666
Start m/z :40.00
End m/z :800.00

Sample Inlet Unit :GC

[MS Program]
Use MS Program :OFF

Spectrum

Line#:1 R.Time:24.9(Scan#:2629)
MassPeaks:15
RawMode:Single 24.9(2629) BasePeak:74(30549)
BG Mode:24.9(2632) Group 1 - Event 1

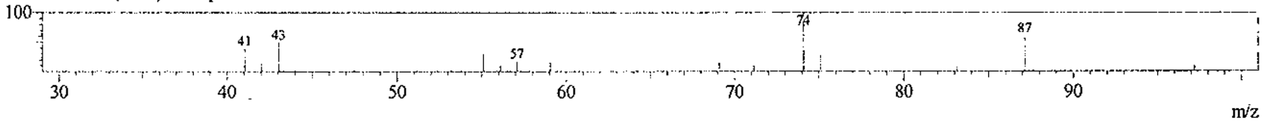

Line#:2 R.Time:27.2(Scan#:2910)
MassPeaks:27
RawMode:Single 27.2(2910) BasePeak:55(16146)
BG Mode:27.3(2913) Group 1 - Event 1

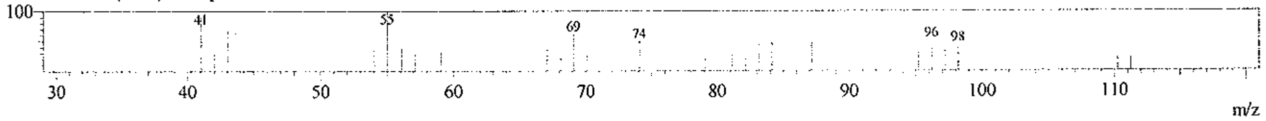

Line#:3 R.Time:27.7(Scan#:2968)
MassPeaks:39
RawMode:Single 27.7(2968) BasePeak:55(41348)
BG Mode:27.8(2972) Group 1 - Event 1

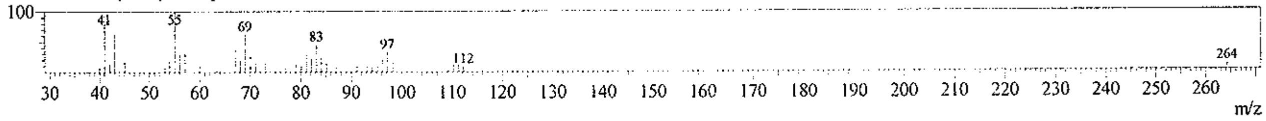

Line#:4 R.Time:28.7(Scan#:3083)
MassPeaks:11
RawMode:Single 28.7(3083) BasePeak:83(3084)
BG Mode:28.7(3085) Group 1 - Event 1

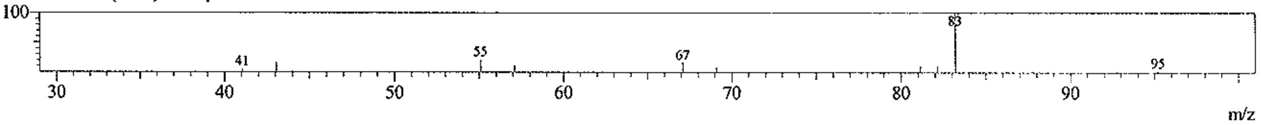

Line#:5 R.Time:29.6(Scan#:3191)
MassPeaks:8
RawMode:Single 29.6(3191) BasePeak:98(3737)
BG Mode:29.6(3193) Group 1 - Event 1

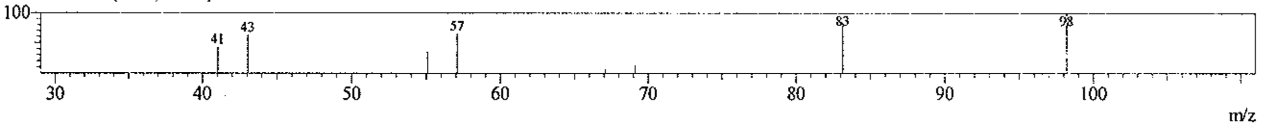

Line#:6 R.Time:31.9(Scan#:3473)
MassPeaks:18
RawMode:Single 31.9(3473) BasePeak:84(4060)
BG Mode:32.0(3477) Group 1 - Event 1

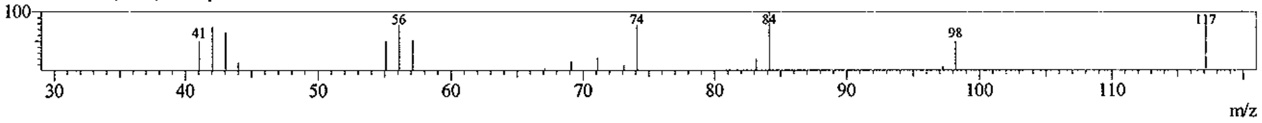

Line#:7 R.Time:32.8(Scan#:3580)
MassPeaks:13
RawMode:Single 32.8(3580) BasePeak:349(10814)
BG Mode:32.8(3577) Group 1 - Event 1

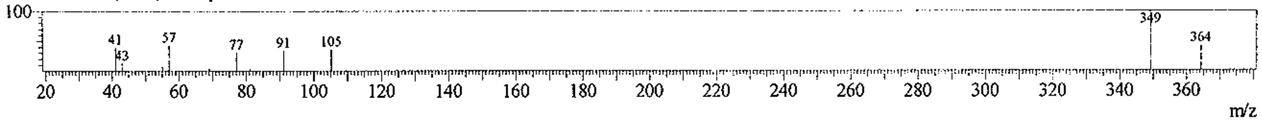

Line#:8 R.Time:33.9(Scan#:3706)
MassPeaks:22
RawMode:Single 33.9(3706) BasePeak:96(3623)
BG Mode:33.9(3709) Group 1 - Event 1

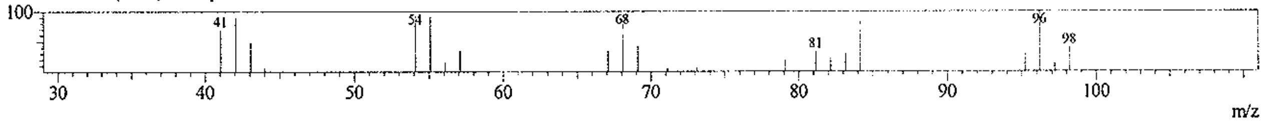

Line#:9 R.Time:35.6(Scan#:3911)
MassPeaks:25
RawMode:Single 35.6(3911) BasePeak:69(42398)
BG Mode:35.6(3914) Group 1 - Event 1

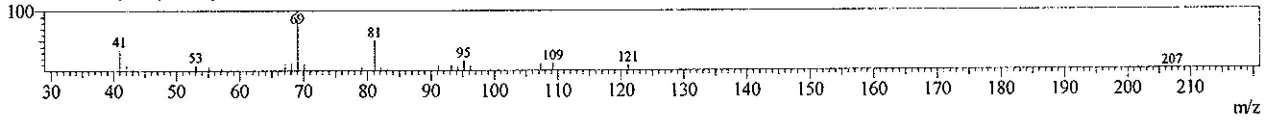

Line#:10 R.Time:38.3(Scan#:4235)
MassPeaks:29
RawMode:Single 38.3(4235) BasePeak:416(51332)
BG Mode:38.3(4241) Group 1 - Event 1

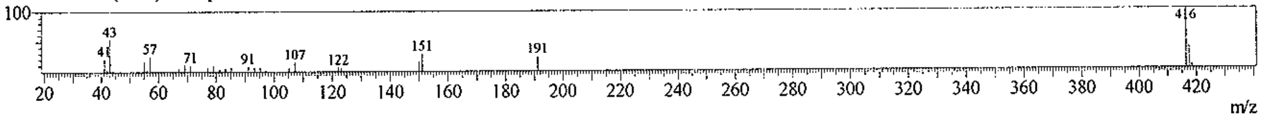

Line#:11 R.Time:39.3(Scan#:4354)
MassPeaks:25
RawMode:Single 39.3(4354) BasePeak:446(29130)
BG Mode:39.3(4359) Group 1 - Event 1

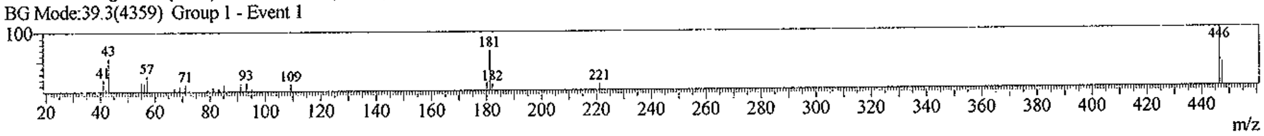

Line#:12 R.Time:39.9(Scan#:4432)
MassPeaks:33
RawMode:Single 39.9(4432) BasePeak:430(79538)
BG Mode:40.0(4437) Group 1 - Event 1

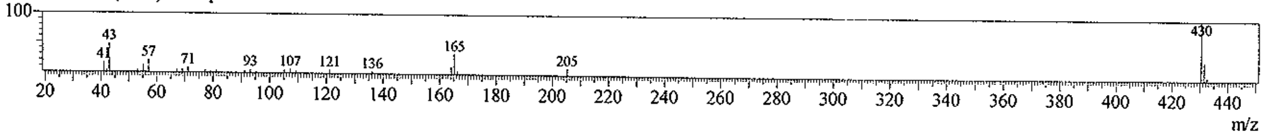

Line#:13 R.Time:42.7(Scan#:4762)
MassPeaks:14
RawMode:Single 42.7(4762) BasePeak:93(3036)
BG Mode:42.7(4768) Group 1 - Event 1

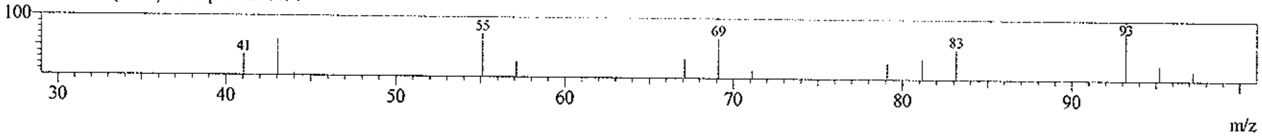

Line#:14 R.Time:44.1(Scan#:4936)
MassPeaks:25
RawMode:Single 44.1(4936) BasePeak:43(9163)
BG Mode:44.2(4944) Group 1 - Event 1

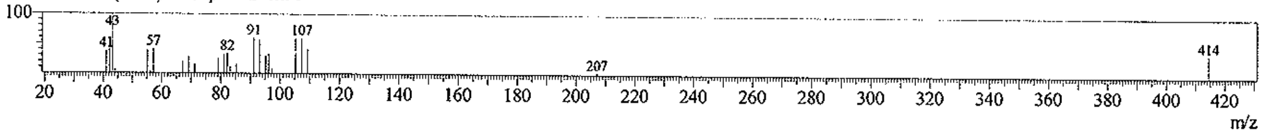

Line#:15 R.Time:45.3(Scan#:5082)
MassPeaks:24
RawMode:Single 45.3(5082) BasePeak:107(6676)
BG Mode:45.4(5091) Group 1 - Event 1

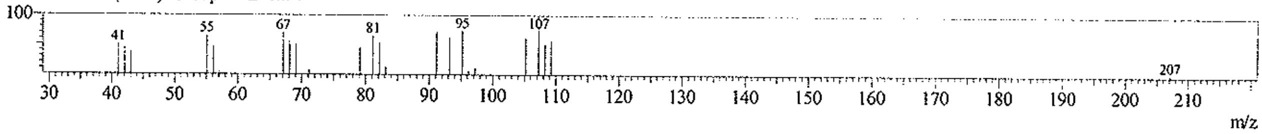

Line#:16 R.Time:46.1(Scan#:5168)
MassPeaks:22
RawMode:Single 46.1(5168) BasePeak:109(4586)
BG Mode:46.1(5177) Group 1 - Event 1

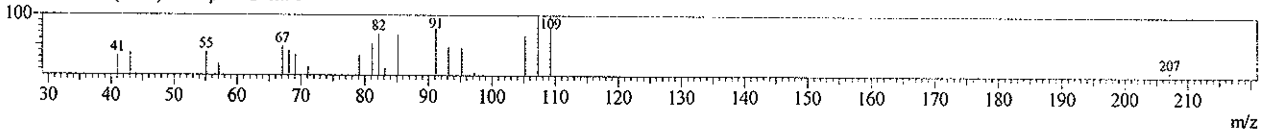

Line#:17 R.Time:38.5(Scan#:4259)
MassPeaks:10
RawMode:Single 38.5(4259) BasePeak:420(9502)
BG Mode:38.5(4264) Group 1 - Event 1

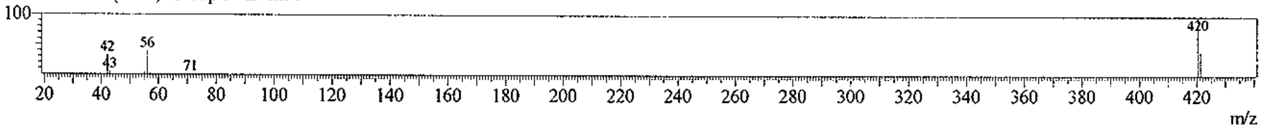

Spectrum Comparison

Library

<< Target >>
Line#:1 R.Time:24.900(Scan#:2629) MassPeaks:15
RawMode:Single 24.900(2629) BasePeak:74.10(30549)
BG Mode:24.925(2632) Group 1 - Event 1

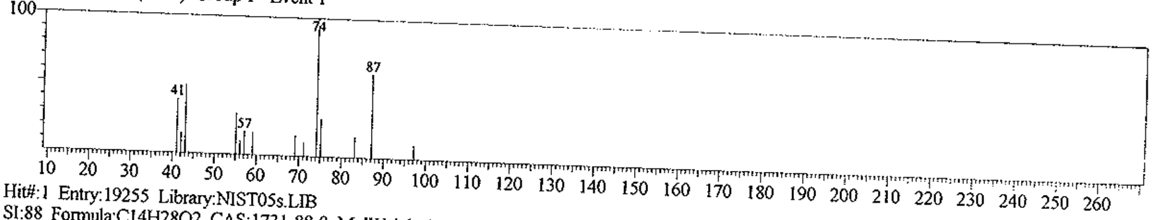

Hit#:1 Entry:19255 Library:NIST05s.LIB
SI:88 Formula:C14H28O2 CAS:1731-88-0 MolWeight:228 RetIndex:1580
CompName:Tridecanoic acid, methyl ester $$ Methyl tridecanoate $$ n-Tridecanoic acid methyl ester $$ Methyl ester of tridecanoic acid $$

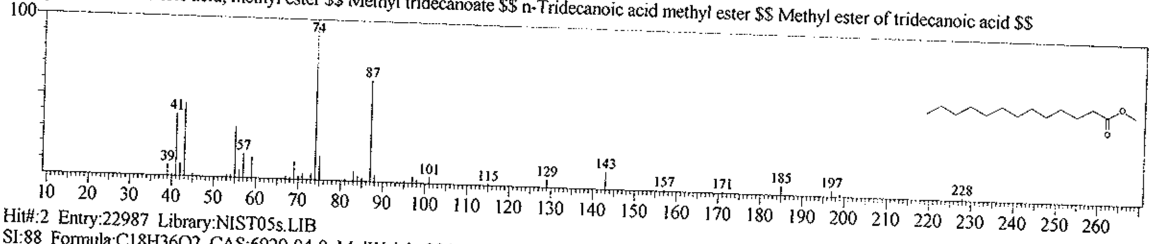

Hit#:2 Entry:22987 Library:NIST05s.LIB
SI:88 Formula:C18H36O2 CAS:6929-04-0 MolWeight:284 RetIndex:1914
CompName:Hexadecanoic acid, 15-methyl-, methyl ester $$ Methyl isoheptadecanoate $$ Methyl 15-methylhexadecanoate $$

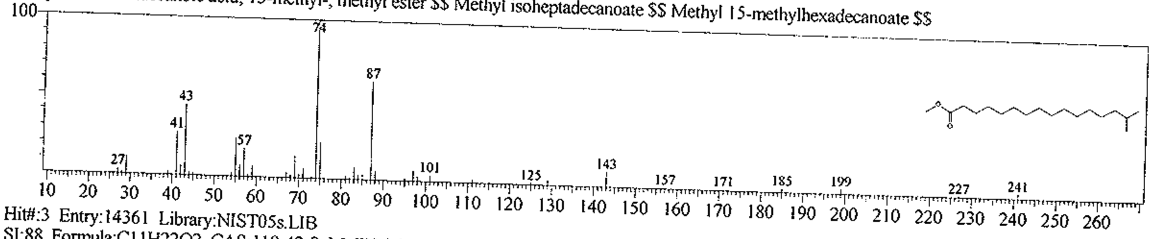

Hit#:3 Entry:14361 Library:NIST05s.LIB
SI:88 Formula:C11H22O2 CAS:110-42-9 MolWeight:186 RetIndex:1282
CompName:Decanoic acid, methyl ester $$ Capric acid methyl ester $$ Metholene 2095 $$ Methyl caprate $$ Methyl caprinate $$ Methyl decanoate $$ Me

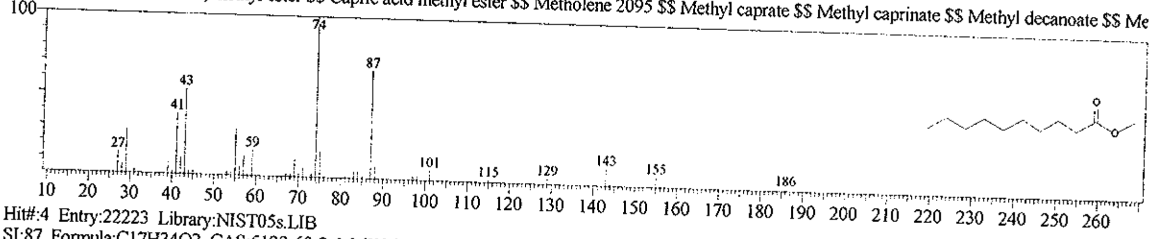

Hit#:4 Entry:22223 Library:NIST05s.LIB
SI:87 Formula:C17H34O2 CAS:5129-60-2 MolWeight:270 RetIndex:1814
CompName:Pentadecanoic acid, 14-methyl-, methyl ester $$ Methyl 14-methylpentadecanoate # $$

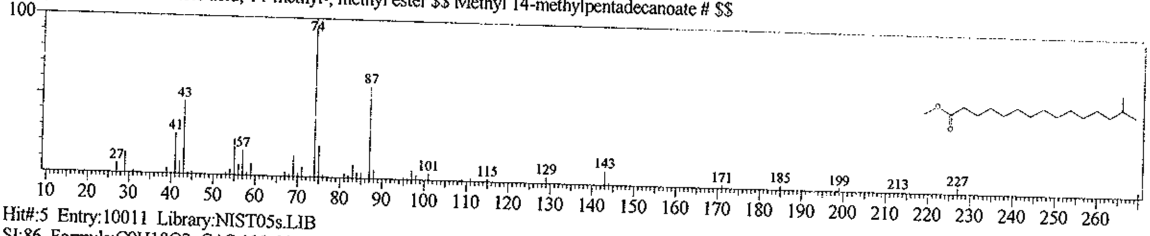

Hit#:5 Entry:10011 Library:NIST05s.LIB
SI:86 Formula:C9H18O2 CAS:111-11-5 MolWeight:158 RetIndex:1083
CompName:Octanoic acid, methyl ester $$ Caprylic acid methyl ester $$ Methyl caprylate $$ Methyl n-octanoate $$ Methyl octanoate $$ Uniphat A20 $$ n

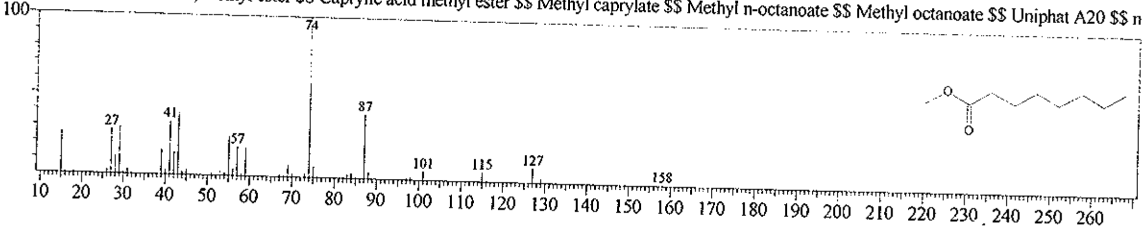

<< Target >>
Line#:2 R.Time:27.242(Scan#:2910) MassPeaks:27
RawMode:Single 27.242(2910) BasePeak:55.05(16146)
BG Mode:27.267(2913) Group 1 - Event 1

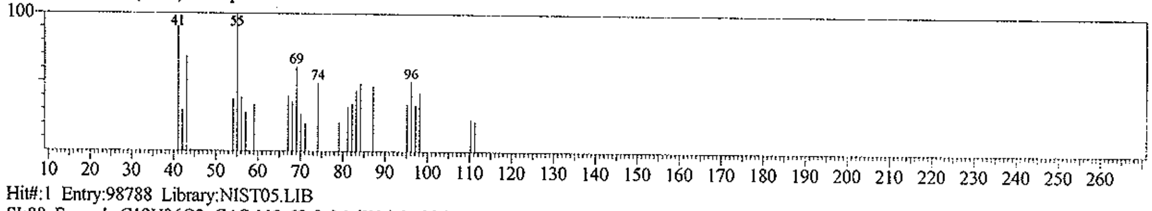

Hit#:1 Entry:98788 Library:NIST05.LIB
SI:88 Formula:C19H36O2 CAS:112-62-9 MolWeight:296 RetIndex:2085
CompName:9-Octadecenoic acid (Z)-, methyl ester $$ Oleic acid, methyl ester $$ Emery oleic acid ester 2301 $$ Methyl cis-9-octadecenoate $$ Methyl ole

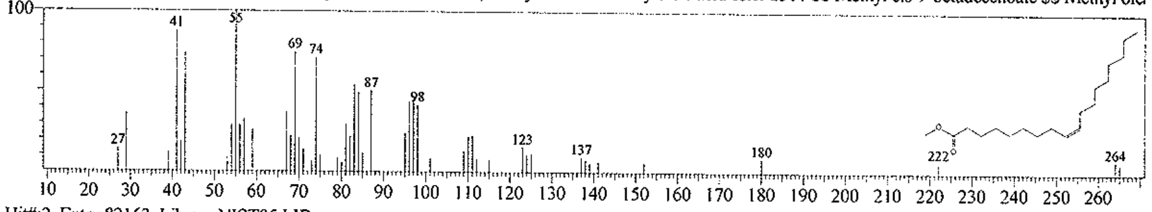

Hit#:2 Entry:82163 Library:NIST05.LIB
SI:88 Formula:C17H32O2 CAS:56875-67-3 MolWeight:268 RetIndex:1886
CompName:7-Hexadecenoic acid, methyl ester, (Z)- $$ Methyl (7E)-7-hexadecenoate # $$

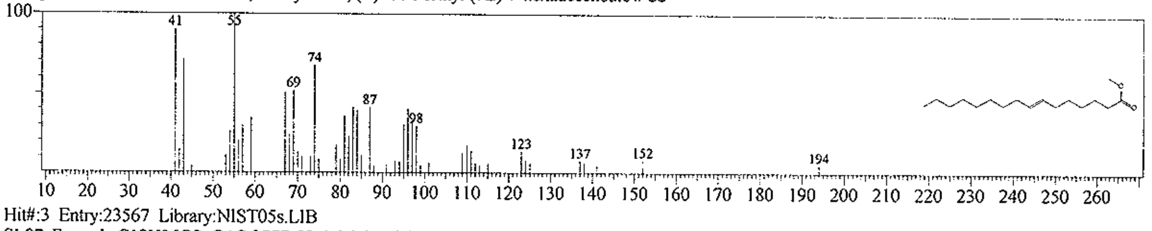

Hit#:3 Entry:23567 Library:NIST05s.LIB
SI:87 Formula:C19H36O2 CAS:2777-58-4 MolWeight:296 RetIndex:2085
CompName:6-Octadecenoic acid, methyl ester, (Z)- $$ Methyl cis-6-octadecenoate $$ Methyl petroselinate $$ Methyl (6Z)-6-octadecenoate # $$

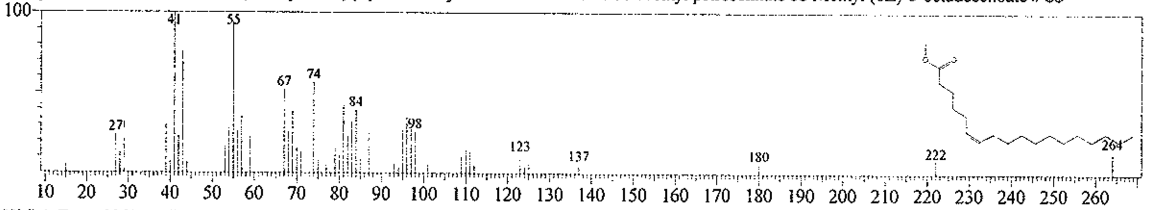

Hit#:4 Entry:22871 Library:NIST05s.LIB
SI:87 Formula:C18H34O2 CAS:10152-61-1 MolWeight:282 RetIndex:1941
CompName:Cyclopropaneoctanoic acid, 2-hexyl-, methyl ester $$ Methyl 8-(2-hexylcyclopropyl)octanoate # $$

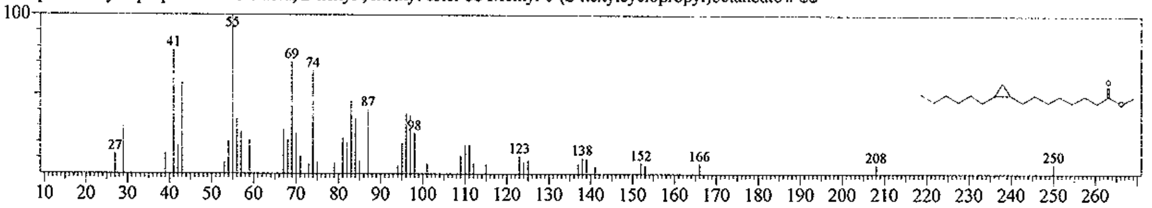

Hit#:5 Entry:23570 Library:NIST05s.LIB
SI:86 Formula:C19H36O2 CAS:1937-62-8 MolWeight:296 RetIndex:2085
CompName:9-Octadecenoic acid, methyl ester, (E)- $$ Elaidic acid, methyl ester $$ Methyl elaidate $$ Methyl trans-9-octadecenoate $$ (E)-9-Octadecenoi

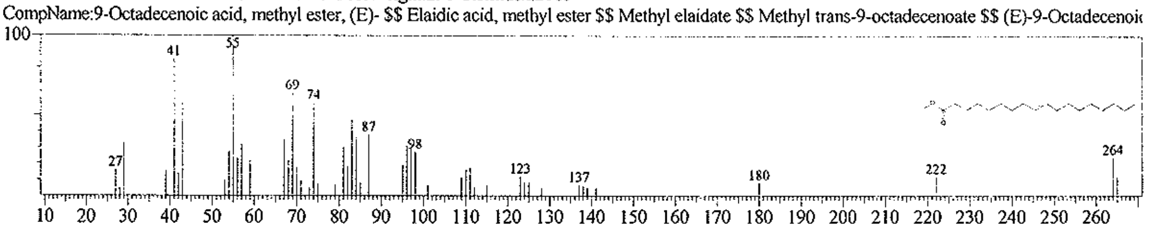

<< Target >>
Line#:3 R.Time:27.725(Scan#:2968) MassPeaks:39
RawMode:Single 27.725(2968) BasePeak:55.10(41348)
BG Mode:27.758(2972) Group 1 - Event 1

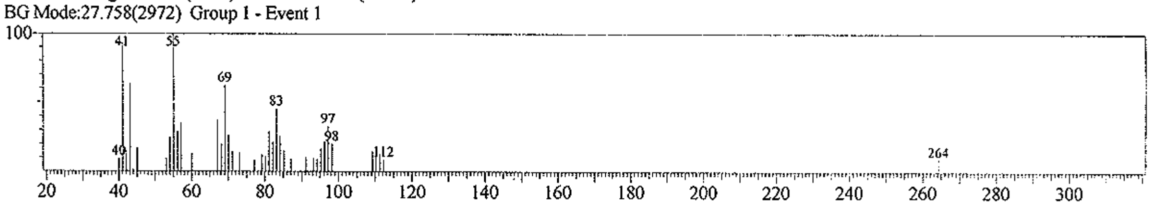

Hit#:1 Entry:73685 Library:NIST05.LIB
SI:89 Formula:C16H30O2 CAS:2091-29-4 MolWeight:254 RetIndex:1976
CompName:9-Hexadecenoic acid $$ (9E)-9-Hexadecenoic acid # $$

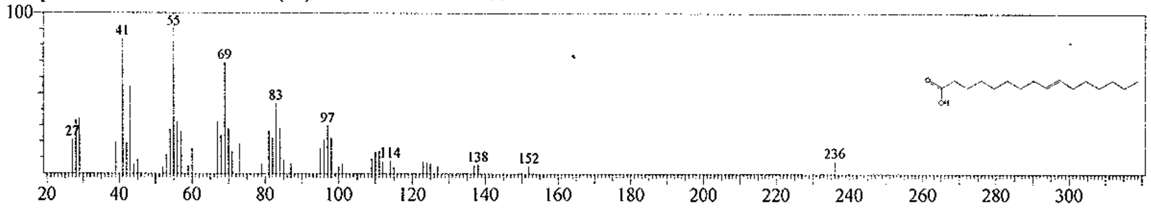

Hit#:2 Entry:121691 Library:NIST05.LIB
SI:88 Formula:C22H42O2 CAS:112-86-7 MolWeight:338 RetIndex:2572
CompName:Erucic acid $$ 13-Docosenoic acid, (Z)- $$ .delta.13-cis-Docosenoic acid $$ cis-13-Docosenoic acid $$ (Z)-13-Docosenoic acid $$ Prifrac 299

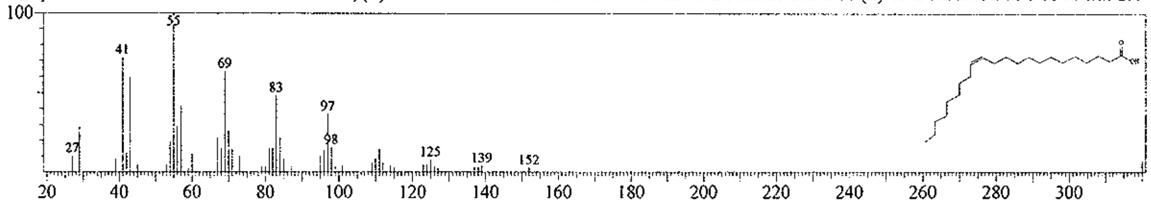

Hit#:3 Entry:90577 Library:NIST05.LIB
SI:88 Formula:C18H34O2 CAS:112-80-1 MolWeight:282 RetIndex:2175
CompName:Oleic Acid $$ 9-Octadecenoic acid (Z)- $$ .delta.(Sup9)-cis-Oleic acid $$ cis-.delta.(Sup9)-Octadecenoic acid $$ cis-Oleic Acid $$ cis-9-Octad

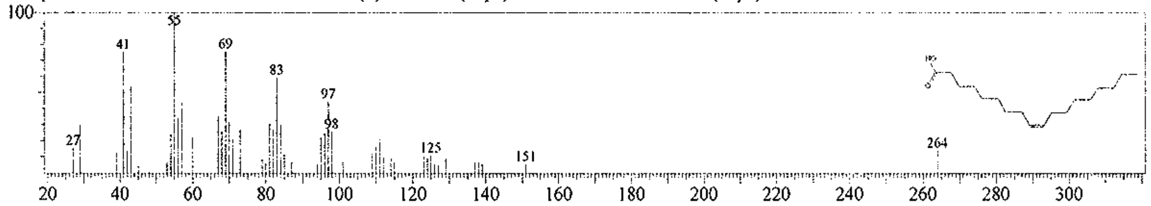

Hit#:4 Entry:22869 Library:NIST05s.LIB
SI:88 Formula:C18H34O2 CAS:112-80-1 MolWeight:282 RetIndex:2175
CompName:Oleic Acid $$ 9-Octadecenoic acid (Z)- $$ .delta.(Sup9)-cis-Oleic acid $$ cis-.delta.(Sup9)-Octadecenoic acid $$ cis-Oleic Acid $$ cis-9-Octad

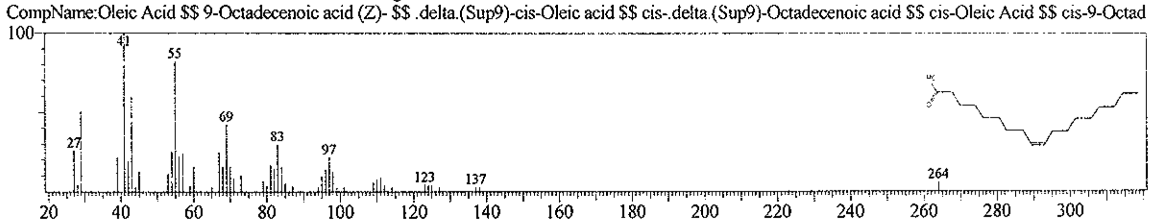

Hit#:5 Entry:121688 Library:NIST05.LIB
SI:88 Formula:C22H42O2 CAS:506-33-2 MolWeight:338 RetIndex:2572
CompName:(E)-13-Docosenoic acid $$ 13-Docosenoic acid, (E)- $$ trans-13-Docosenoic acid $$ Brassidic acid $$ (13E)-13-Docosenoic acid # $$

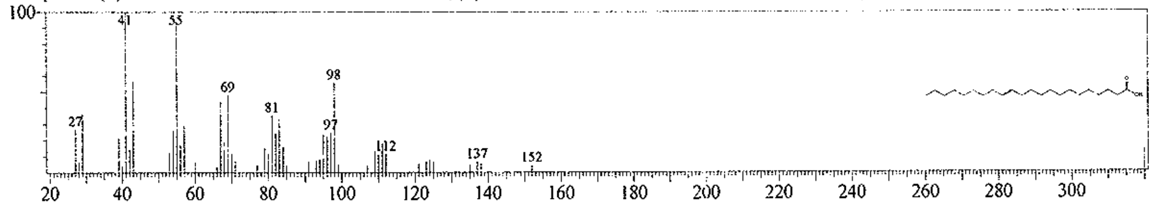

<< Target >>
Line#:4 R.Time:28.683(Scan#:3083) MassPeaks:11
RawMode:Single 28.683(3083) BasePeak:83.20(3084)
BG Mode:28.700(3085) Group 1 - Event 1

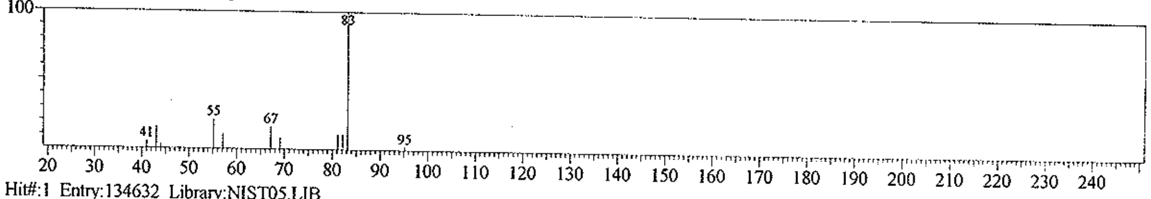

Hit#:1 Entry:134632 Library:NIST05.LIB
SI:85 Formula:C22H40O4 CAS:0-00-0 MolWeight:368 RetIndex:2606
CompName:Oxalic acid, cyclohexyl tetradecyl ester

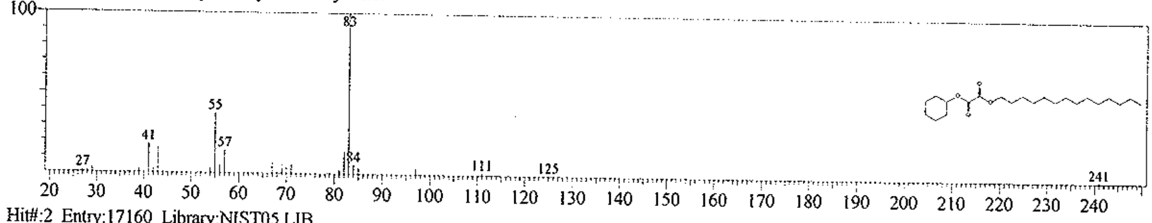

Hit#:2 Entry:17160 Library:NIST05.LIB
SI:85 Formula:C11H22 CAS:74421-06-0 MolWeight:154 RetIndex:972
CompName:2-Heptene, 5-ethyl-2,4-dimethyl- $$ 5-Ethyl-2,4-dimethyl-2-heptene # $$

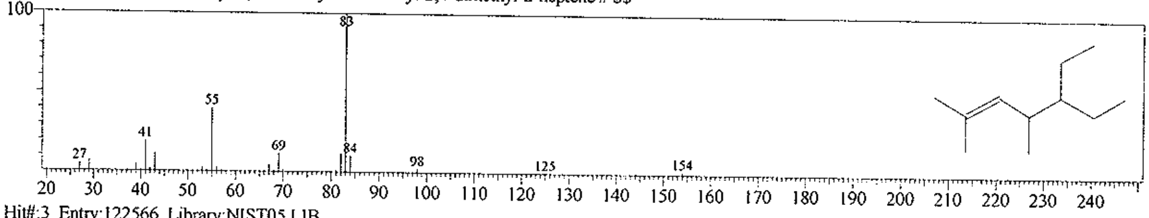

Hit#:3 Entry:122566 Library:NIST05.LIB
SI:84 Formula:C20H36O4 CAS:0-00-0 MolWeight:340 RetIndex:2407
CompName:Oxalic acid, cyclohexyl dodecyl ester

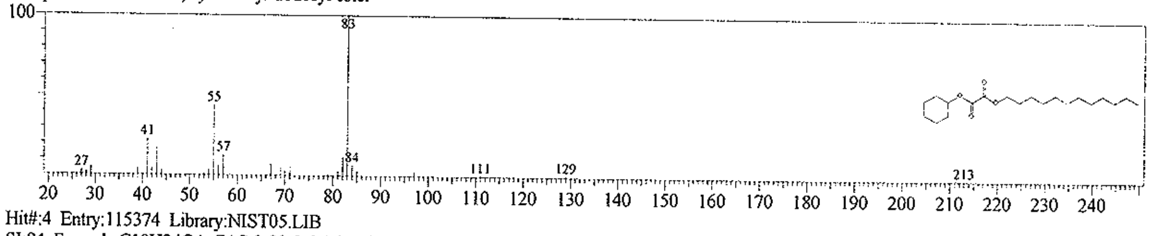

Hit#:4 Entry:115374 Library:NIST05.LIB
SI:84 Formula:C19H34O4 CAS:0-00-0 MolWeight:326 RetIndex:2308
CompName:Oxalic acid, cyclohexyl undecyl ester

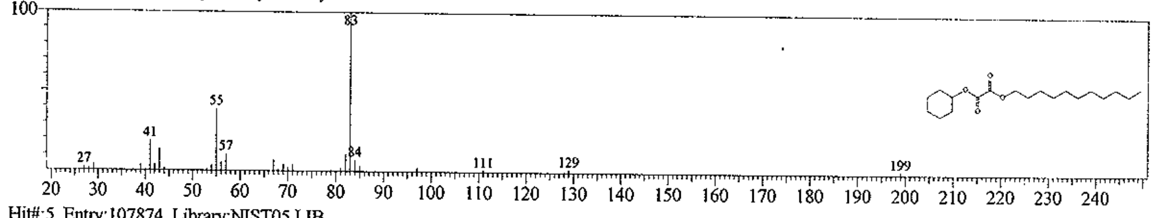

Hit#:5 Entry:107874 Library:NIST05.LIB
SI:84 Formula:C18H32O4 CAS:0-00-0 MolWeight:312 RetIndex:2209
CompName:Oxalic acid, cyclohexyl decyl ester

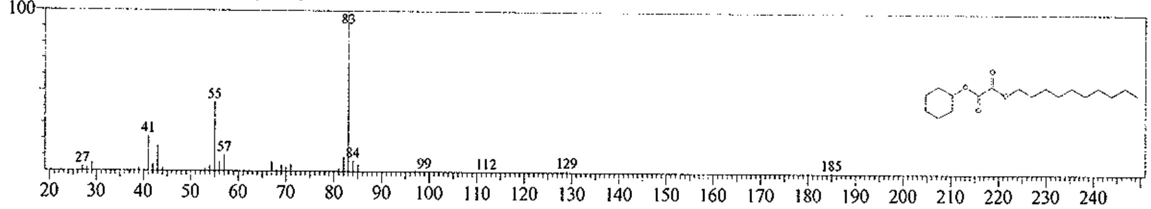

<< Target >>
Line#:5 R.Time:29.583(Scan#:3191) MassPeaks:8
RawMode:Single 29.583(3191) BasePeak:98.20(3737)
BG Mode:29.600(3193) Group 1 - Event 1

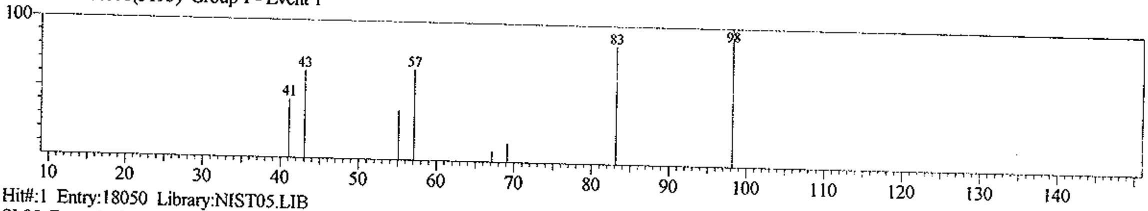

Hit#:1 Entry:18050 Library:NIST05.LIB
SI:85 Formula:C10H20O CAS:0-00-0 MolWeight:156 RetIndex:992
CompName:2,2,3,3,4,4-Hexamethyltetrahydrofuran

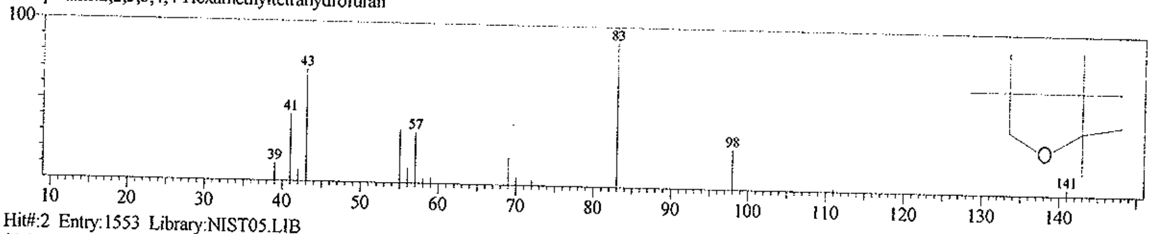

Hit#:2 Entry:1553 Library:NIST05.LIB
SI:81 Formula:C5H6O2 CAS:25414-22-6 MolWeight:98 RetIndex:718
CompName:Furan, 2-methoxy- $$ 2-Methoxyfuran $$ 2-Furyl methyl ether # $$

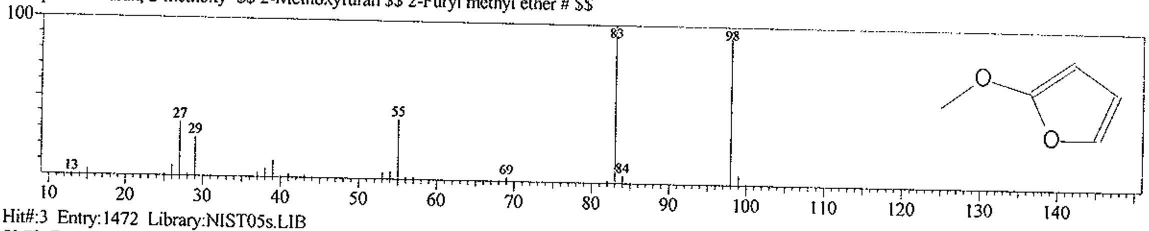

Hit#:3 Entry:1472 Library:NIST05s.LIB
SI:79 Formula:C5H6O2 CAS:25414-22-6 MolWeight:98 RetIndex:718
CompName:Furan, 2-methoxy- $$ 2-Methoxyfuran $$ 2-Furyl methyl ether # $$

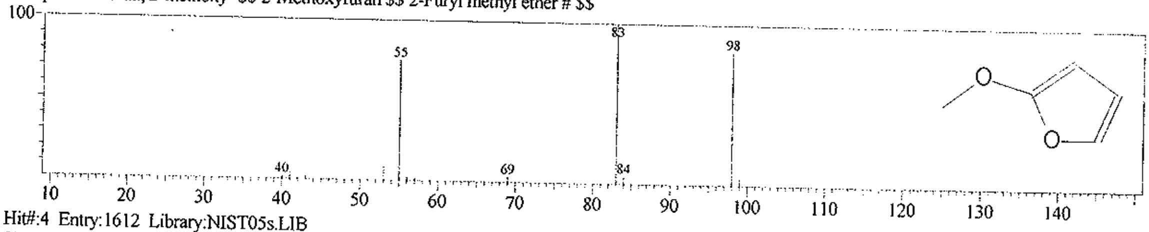

Hit#:4 Entry:1612 Library:NIST05s.LIB
SI:78 Formula:C7H14 CAS:762-63-0 MolWeight:98 RetIndex:641
CompName:2-Pentene, 4,4-dimethyl-, (Z)- $$ (Z)-4,4-Dimethyl-2-pentene $$ cis-4,4-Dimethyl-2-Pentene $$ (Z)-(CH3)3CCH=CHCH3 $$ 4,4-Dimethyl-cis

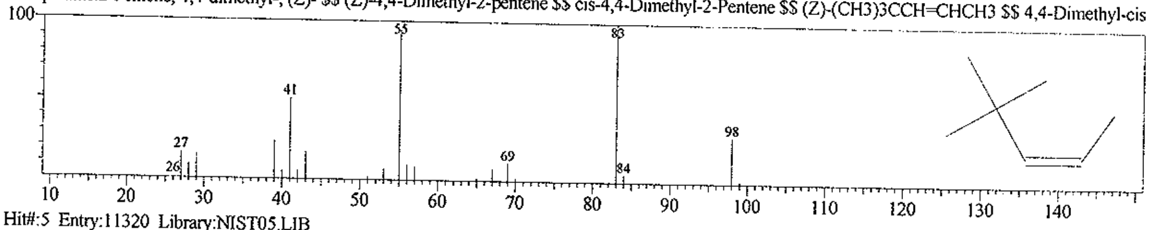

Hit#:5 Entry:11320 Library:NIST05.LIB
SI:78 Formula:C7H11NO2 CAS:42282-85-9 MolWeight:141 RetIndex:1148
CompName:2,4-Azetidinedione, 3,3-diethyl- $$ Malonimide, 2,2-diethyl- $$ 3,3-Diethylazetidin-2,4-dione $$

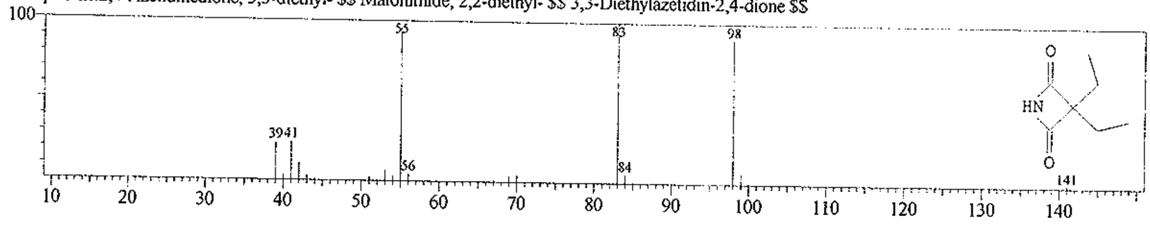

<< Target >>
Line#:6 R.Time:31.933(Scan#:3473) MassPeaks:18
RawMode:Single 31.933(3473) BasePeak:84.15(4060)
BG Mode:31.967(3477) Group 1 - Event 1

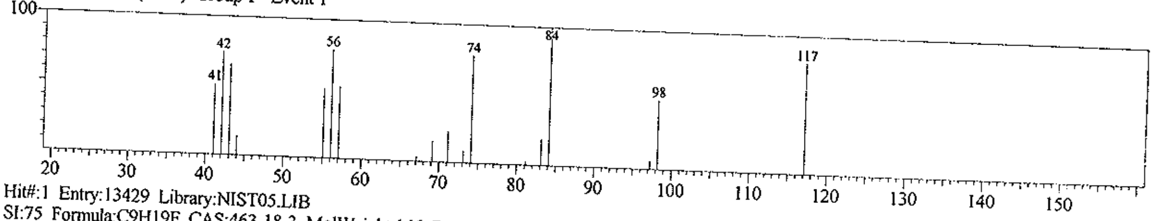

Hit#:1 Entry:13429 Library:NIST05.LIB
SI:75 Formula:C9H19F CAS:463-18-3 MolWeight:146 RetIndex:889
CompName:1-Fluorononane $$ n-Nonyl fluoride $$ Nonane, 1-fluoro- $$

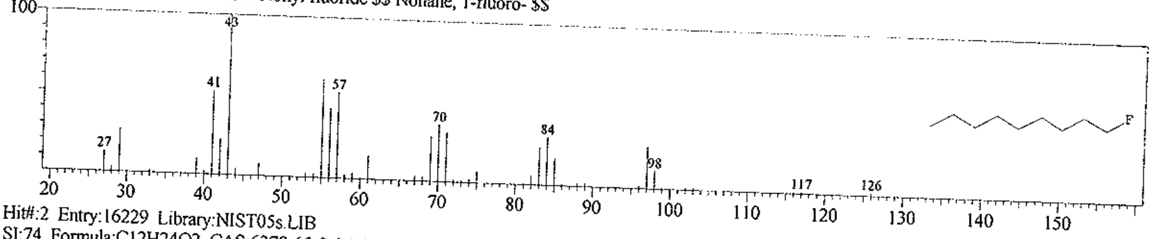

Hit#:2 Entry:16229 Library:NIST05s.LIB
SI:74 Formula:C12H24O2 CAS:6378-65-0 MolWeight:200 RetIndex:1381
CompName:Hexanoic acid, hexyl ester $$ n-Hexyl hexanoate $$ Hexyl caproate $$ Hexyl hexanoate $$ Hexyl hexoate $$ n-Hexyl caproate $$ n-Hexyl n-h

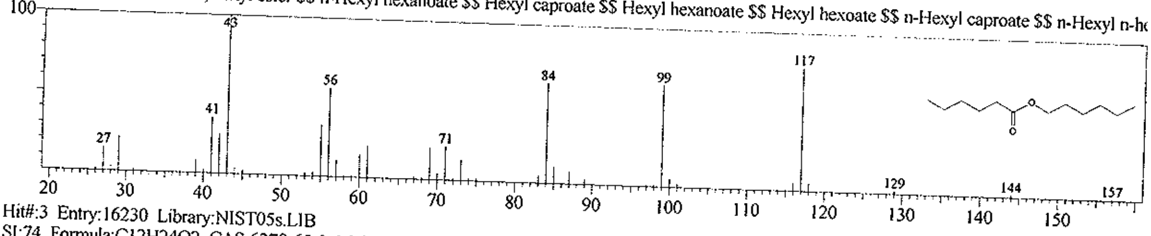

Hit#:3 Entry:16230 Library:NIST05s.LIB
SI:74 Formula:C12H24O2 CAS:6378-65-0 MolWeight:200 RetIndex:1381
CompName:Hexanoic acid, hexyl ester $$ n-Hexyl hexanoate $$ Hexyl caproate $$ Hexyl hexanoate $$ Hexyl hexoate $$ n-Hexyl caproate $$ n-Hexyl n-h

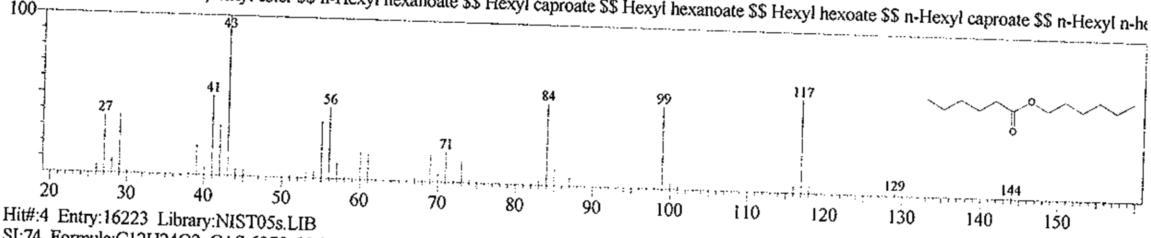

Hit#:4 Entry:16223 Library:NIST05s.LIB
SI:74 Formula:C12H24O2 CAS:6378-65-0 MolWeight:200 RetIndex:1381
CompName:Hexanoic acid, hexyl ester $$ n-Hexyl hexanoate $$ Hexyl caproate $$ Hexyl hexanoate $$ Hexyl hexoate $$ n-Hexyl caproate $$ n-Hexyl n-h

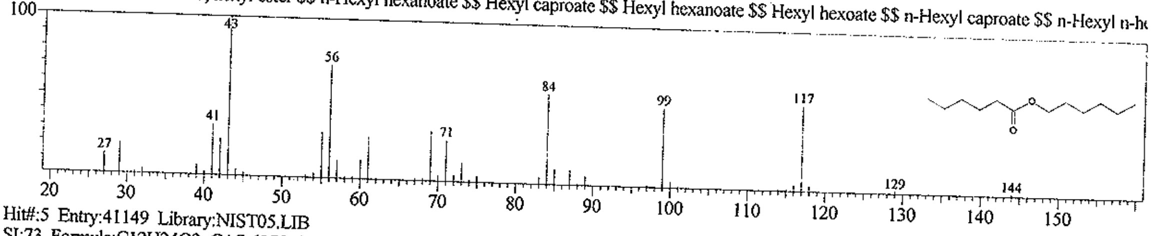

Hit#:5 Entry:41149 Library:NIST05.LIB
SI:73 Formula:C12H24O2 CAS:6378-65-0 MolWeight:200 RetIndex:1381
CompName:Hexanoic acid, hexyl ester $$ n-Hexyl hexanoate $$ Hexyl caproate $$ Hexyl hexanoate $$ Hexyl hexoate $$ n-Hexyl caproate $$ n-Hexyl n-h

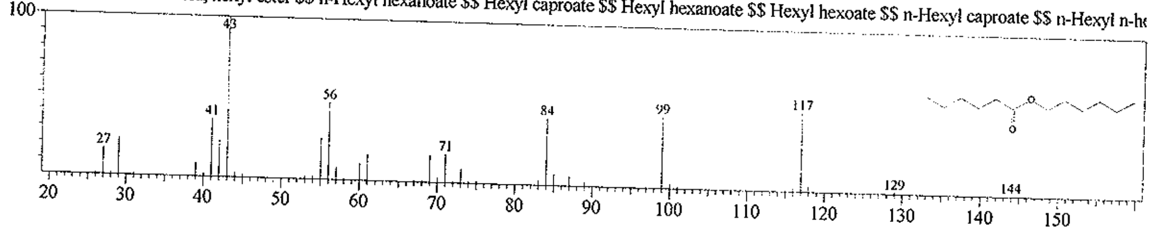

<< Target >>
Line#:7 R.Time:32.825(Scan#:3580) MassPeaks:13
RawMode:Single 32.825(3580) BasePeak:349.25(10814)
BG Mode:32.800(3577) Group 1 - Event 1

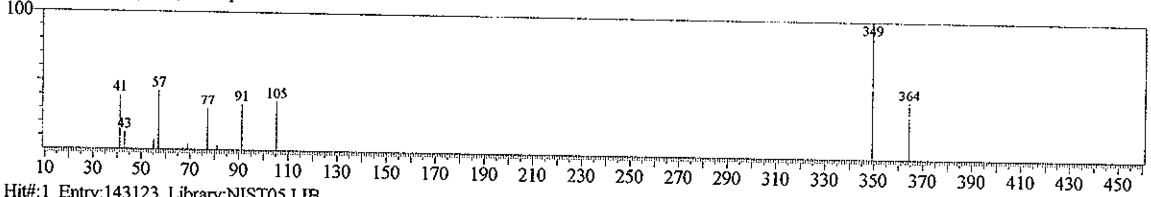

Hit#:1 Entry:143123 Library:NIST05.LIB
SI:55 Formula:C21H22N4O4 CAS:162710-50-1 MolWeight:394 RetIndex:3353
CompName:3,8-Dibenzoyl-1-nitro-3,6,8-triazabicyclo[4.3.1]decane $$ 4,8-Dibenzoyl-6-nitro-1,4,8-triazabicyclo[4.3.1]decane # $$

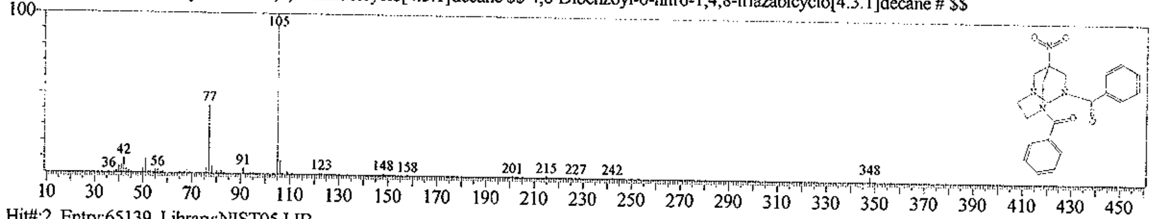

Hit#:2 Entry:65139 Library:NIST05.LIB
SI:55 Formula:C14H21ClO CAS:0-00-0 MolWeight:240 RetIndex:1582
CompName:6-Chloro-2,2,9,9-tetramethyl-3,7-decadiyn-5-ol

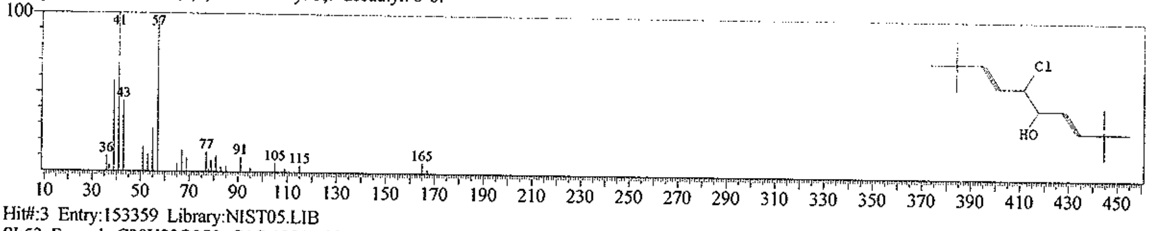

Hit#:3 Entry:153359 Library:NIST05.LIB
SI:53 Formula:C28H22O2S2 CAS:19817-39-1 MolWeight:454 RetIndex:3912
CompName:4,4'-Bis(phenacylthio)biphenyl $$ 2-((4'-[(2-Oxo-2-phenylethyl)sulfanyl][1,1'-biphenyl]-4-yl)sulfanyl)-1-phenylethanone # $$

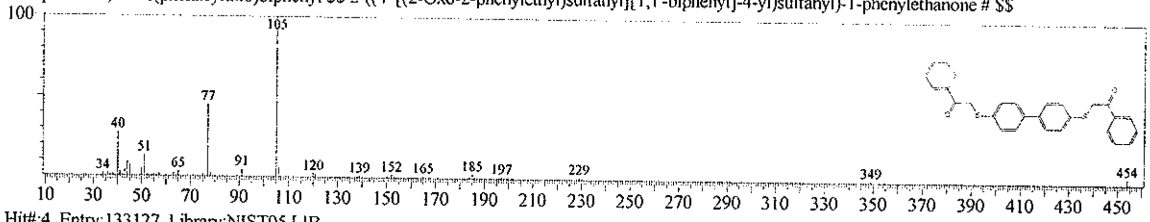

Hit#:4 Entry:133127 Library:NIST05.LIB
SI:53 Formula:C23H24O4 CAS:3253-39-2 MolWeight:364 RetIndex:2673
CompName:2-Propenoic acid, 2-methyl-, (1-methylethylidene)di-4,1-phenylene ester $$ Bisphenol A dimethacrylate $$ 4,4'-Isopropylidenediphenol dimeth

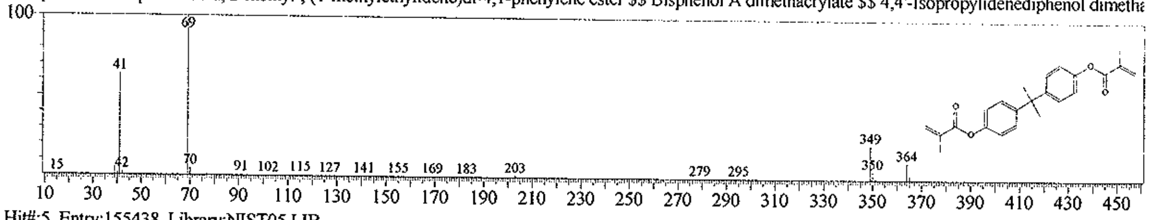

Hit#:5 Entry:155438 Library:NIST05.LIB
SI:52 Formula:C27H24O8 CAS:0-00-0 MolWeight:476 RetIndex:3529
CompName:Methyl tri-O-benzoyl-.alpha.-d-arabinofuranoside

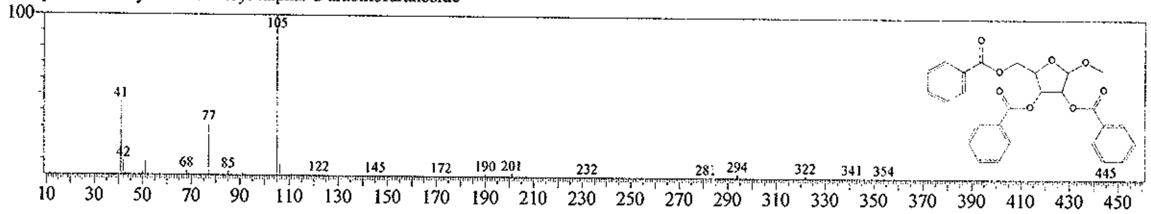

<< Target >>
Line#:8 R.Time:33.875(Scan#:3706) MassPeaks:22
RawMode:Single 33.875(3706) BasePeak:96.20(3623)
BG Mode:33.900(3709) Group 1 - Event 1

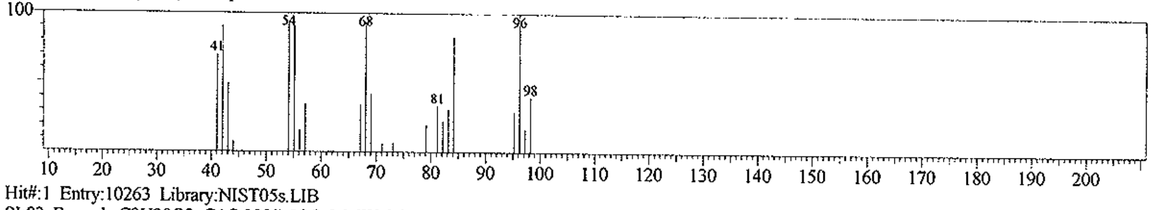

Hit#:1 Entry:10263 Library:NIST05s.LIB
SI:83 Formula:C9H20O2 CAS:3937-56-2 MolWeight:160 RetIndex:1401
CompName:1,9-Nonanediol $$ .alpha.,.omega.-Nonanediol $$

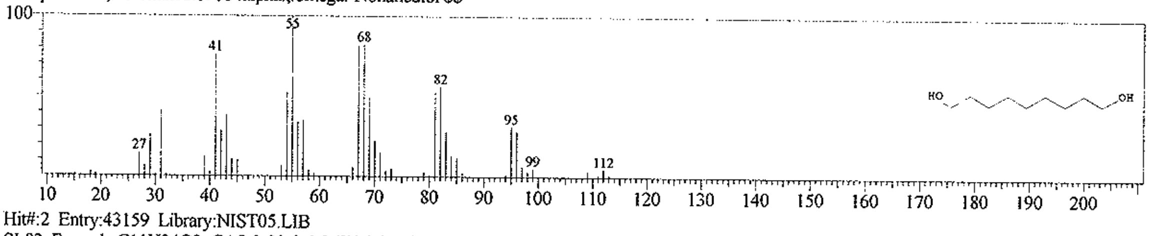

Hit#:2 Entry:43159 Library:NIST05.LIB
SI:83 Formula:C11H24O3 CAS:0-00-0 MolWeight:204 RetIndex:1676
CompName:Nonamethylene glycol

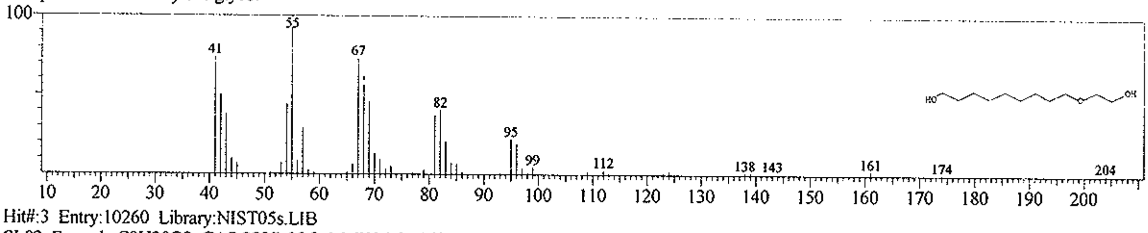

Hit#:3 Entry:10260 Library:NIST05s.LIB
SI:82 Formula:C9H20O2 CAS:3937-56-2 MolWeight:160 RetIndex:1401
CompName:1,9-Nonanediol $$ .alpha.,.omega.-Nonanediol $$

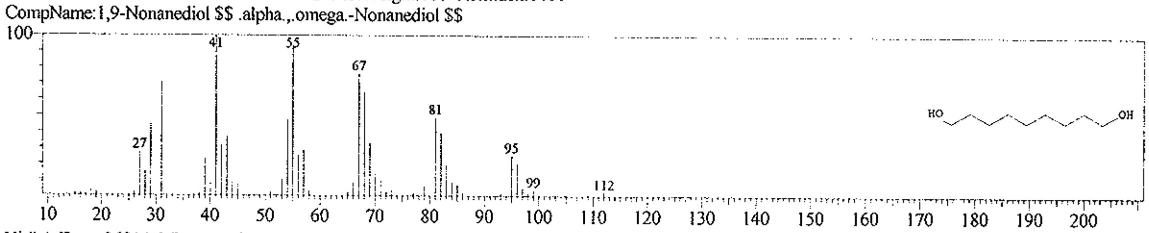

Hit#:4 Entry:26816 Library:NIST05.LIB
SI:82 Formula:C10H22O2 CAS:112-47-0 MolWeight:174 RetIndex:1501
CompName:1,10-Decanediol $$ Decamethylene glycol $$ Decamethylenediol $$ Decane-1,10-diol $$ 1,10-Decamethylene diol $$ 1,6-Bis(2-hydroxyethyl)

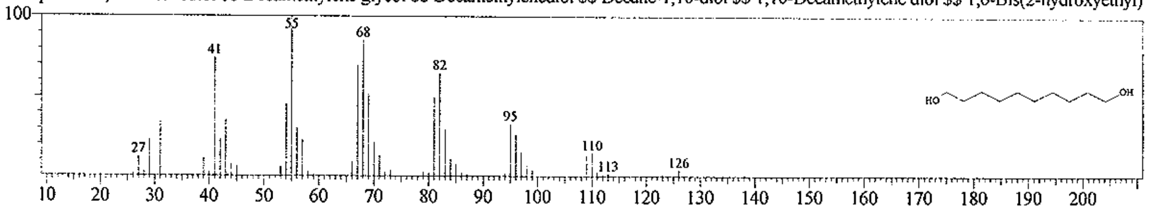

Hit#:5 Entry:12543 Library:NIST05s.LIB
SI:81 Formula:C10H22O2 CAS:112-47-0 MolWeight:174 RetIndex:1501
CompName:1,10-Decanediol $$ Decamethylene glycol $$ Decamethylenediol $$ Decane-1,10-diol $$ 1,10-Decamethylene diol $$ 1,6-Bis(2-hydroxyethyl)

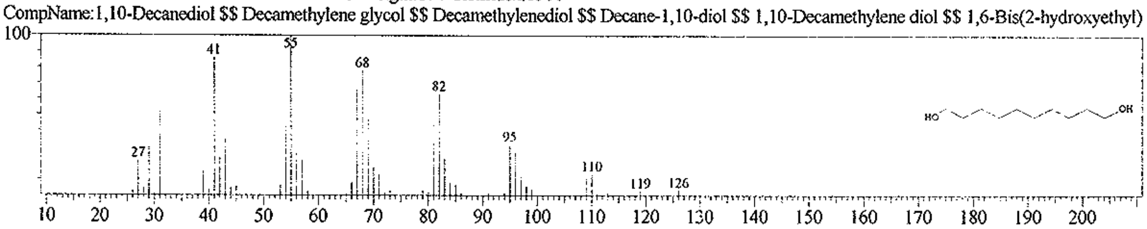

<< Target >>
Line#:9 R.Time:35.583(Scan#:3911) MassPeaks:25
RawMode:Single 35.583(3911) BasePeak:69.10(42398)
BG Mode:35.608(3914) Group 1 - Event 1

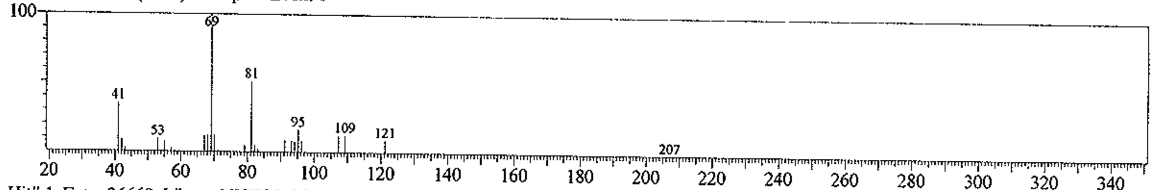

Hit#:1 Entry:26669 Library:NIST05s.LIB
SI:86 Formula:C30H50 CAS:7683-64-9 MolWeight:410 RetIndex:2914
CompName:Squalene $$ 2,6,10,14,18,22-Tetracosahexaene, 2,6,10,15,19,23-hexamethyl- $$ Skvalen $$ Spinacene $$ Supraene $$ (6E,10E,14E,18E)-2,6,

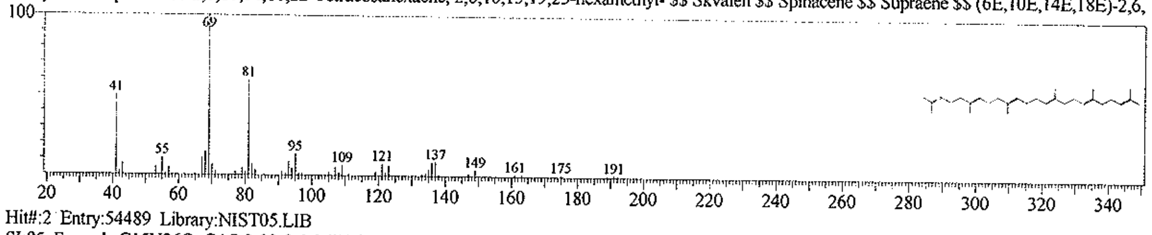

Hit#:2 Entry:54489 Library:NIST05.LIB
SI:85 Formula:C15H26O CAS:0-00-0 MolWeight:222 RetIndex:1710
CompName:Farnesol isomer a

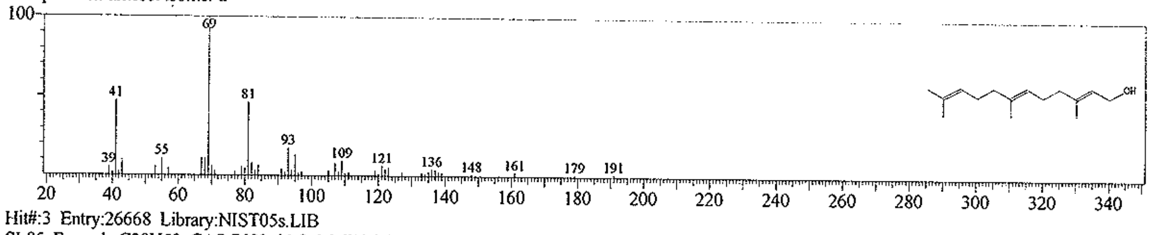

Hit#:3 Entry:26668 Library:NIST05s.LIB
SI:85 Formula:C30H50 CAS:7683-64-9 MolWeight:410 RetIndex:2914
CompName:Squalene $$ 2,6,10,14,18,22-Tetracosahexaene, 2,6,10,15,19,23-hexamethyl- $$ Skvalen $$ Spinacene $$ Supraene $$ (6E,10E,14E,18E)-2,6,

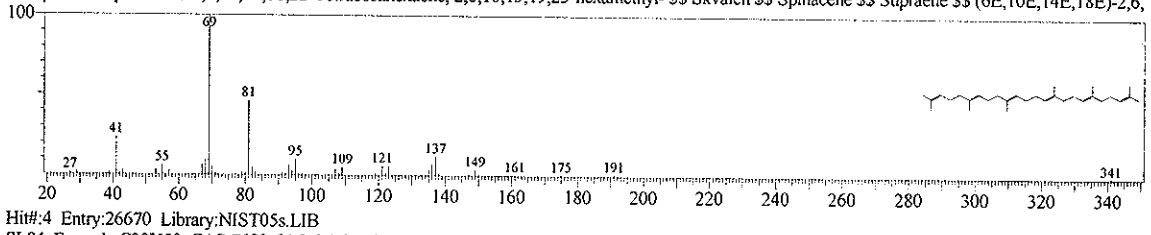

Hit#:4 Entry:26670 Library:NIST05s.LIB
SI:84 Formula:C30H50 CAS:7683-64-9 MolWeight:410 RetIndex:2914
CompName:Squalene $$ 2,6,10,14,18,22-Tetracosahexaene, 2,6,10,15,19,23-hexamethyl- $$ Skvalen $$ Spinacene $$ Supraene $$ (6E,10E,14E,18E)-2,6,

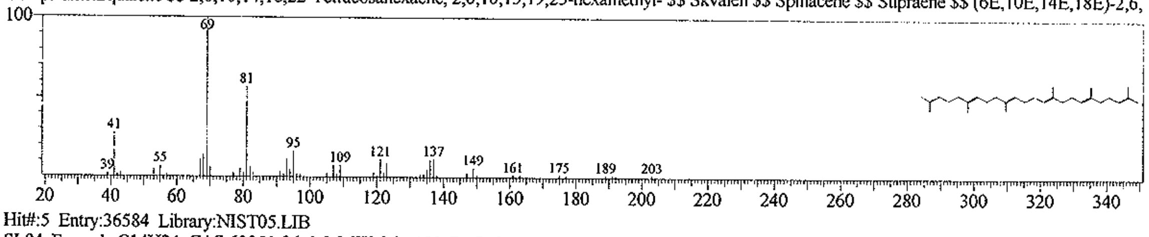

Hit#:5 Entry:36584 Library:NIST05s.LIB
SI:84 Formula:C14H24 CAS:62951-96-6 MolWeight:192 RetIndex:1350
CompName:1,5,9-Undecatriene, 2,6,10-trimethyl-, (Z)- $$ (5Z)-2,6,10-Trimethyl-1,5,9-undecatriene # $$

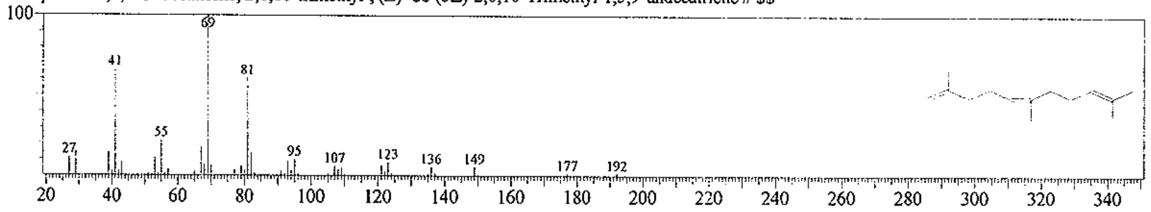

<< Target >>
Line#:10 R.Time:38.283(Scan#:4235) MassPeaks:29
RawMode:Single 38.283(4235) BasePeak:416.45(51332)
BG Mode:38.333(4241) Group 1 - Event 1

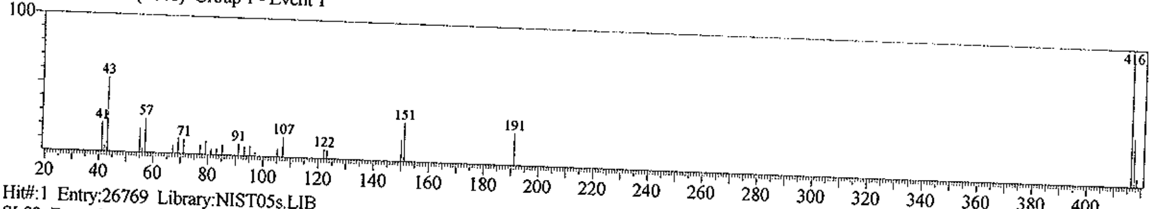

Hit#:1 Entry:26769 Library:NIST05s.LIB
SI:83 Formula:C28H48O2 CAS:148-03-8 MolWeight:416 RetIndex:3036
CompName:.beta.-Tocopherol $$ 2H-1-Benzopyran-6-ol, 3,4-dihydro-2,5,8-trimethyl-2-(4,8,12-trimethyltridecyl)- $$ 6-Chromanol, 2,5,8-trimethyl-2-(4,8,1

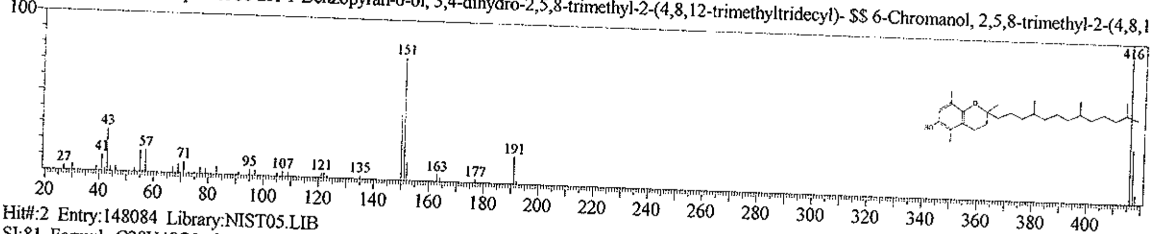

Hit#:2 Entry:148084 Library:NIST05.LIB
SI:81 Formula:C28H48O2 CAS:148-03-8 MolWeight:416 RetIndex:3036
CompName:.beta.-Tocopherol $$ 2H-1-Benzopyran-6-ol, 3,4-dihydro-2,5,8-trimethyl-2-(4,8,12-trimethyltridecyl)- $$ 6-Chromanol, 2,5,8-trimethyl-2-(4,8,1

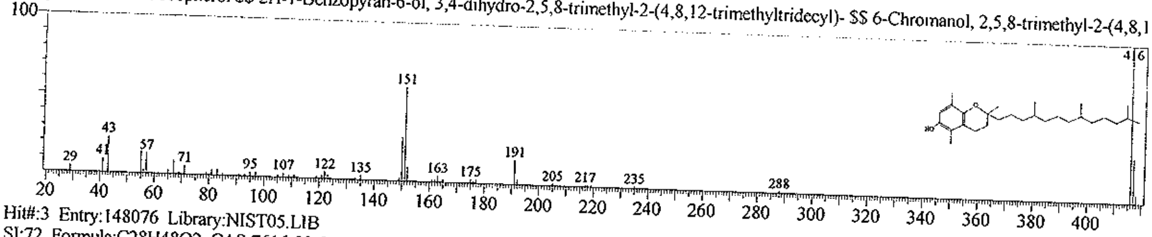

Hit#:3 Entry:148076 Library:NIST05.LIB
SI:72 Formula:C28H48O2 CAS:7616-22-0 MolWeight:416 RetIndex:3036
CompName:.gamma.-Tocopherol $$ 2H-1-Benzopyran-6-ol, 3,4-dihydro-2,7,8-trimethyl-2-(4,8,12-trimethyltridecyl)- $$ 6-Chromanol, 2,7,8-trimethyl-2-(4,

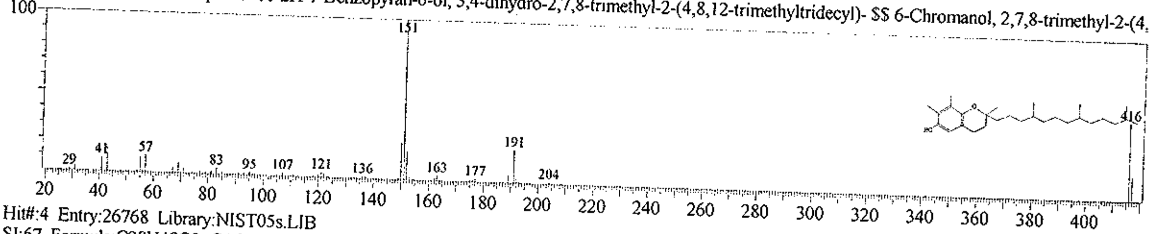

Hit#:4 Entry:26768 Library:NIST05s.LIB
SI:67 Formula:C28H48O2 CAS:7616-22-0 MolWeight:416 RetIndex:3036
CompName:.gamma.-Tocopherol $$ 2H-1-Benzopyran-6-ol, 3,4-dihydro-2,7,8-trimethyl-2-(4,8,12-trimethyltridecyl)- $$ 6-Chromanol, 2,7,8-trimethyl-2-(4,

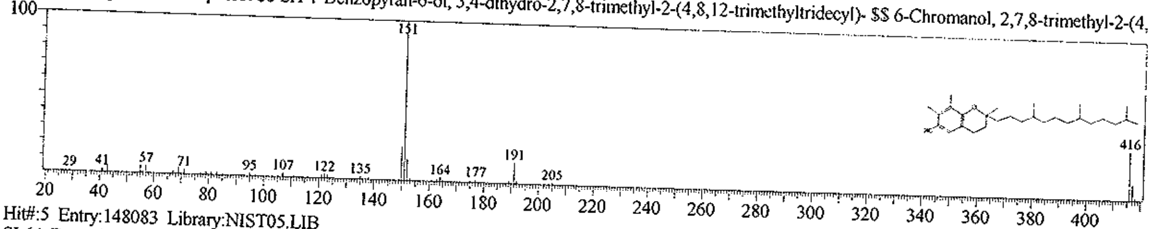

Hit#:5 Entry:148083 Library:NIST05.LIB
SI:64 Formula:C28H48O2 CAS:5837-39-8 MolWeight:416 RetIndex:2706
CompName:Cholestan-6-one, 3-methoxy-, (3.beta.,5.alpha.)- $$ 5.alpha.-Cholestan-6-one, 3.beta.-methoxy- $$ 3.beta.-Methoxy-5.alpha.-cholesten-6-one $$

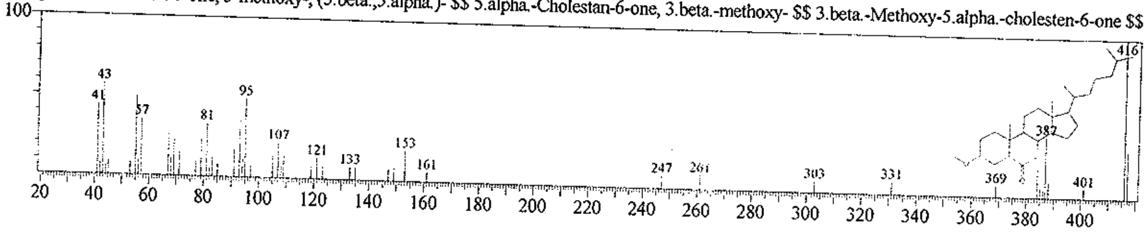

<< Target >>
Line#:11 R.Time:39.275(Scan#:4354) MassPeaks:25
RawMode:Single 39.275(4354) BasePeak:446.45(29130)
BG Mode:39.317(4359) Group 1 - Event 1

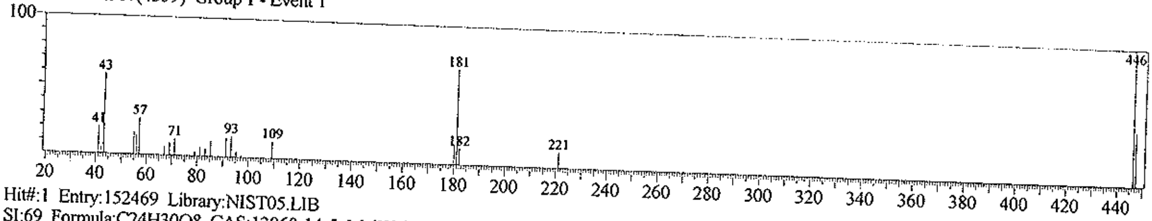

Hit#:1 Entry:152469 Library:NIST05.LIB
SI:69 Formula:C24H30O8 CAS:13060-14-5 MolWeight:446 RetIndex:3243
CompName:1H,3H-Furo[3,4-c]furan, tetrahydro-1,4-bis(3,4,5-trimethoxyphenyl)-, [1S-(1.alpha.,3a.alpha.,4.alpha.,6a.alpha.)]- $$ 1H,3H-Furo[3,4-c]furan, :

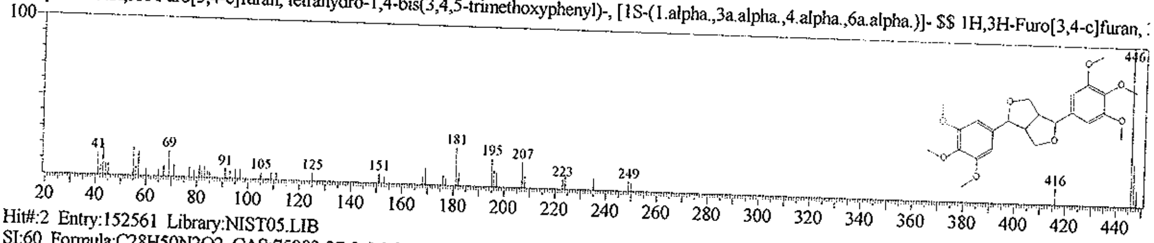

Hit#:2 Entry:152561 Library:NIST05.LIB
SI:60 Formula:C28H50N2O2 CAS:75083-37-3 MolWeight:446 RetIndex:3413
CompName:2,5-Cyclohexadiene-1,4-dione, 2,5-bis(aminomethyl)-3-(2,6,10,14-tetramethylhexadecyl)- $$ 2,5-Bis(aminomethyl)-3-(2,6,10,14-tetramethylhe:

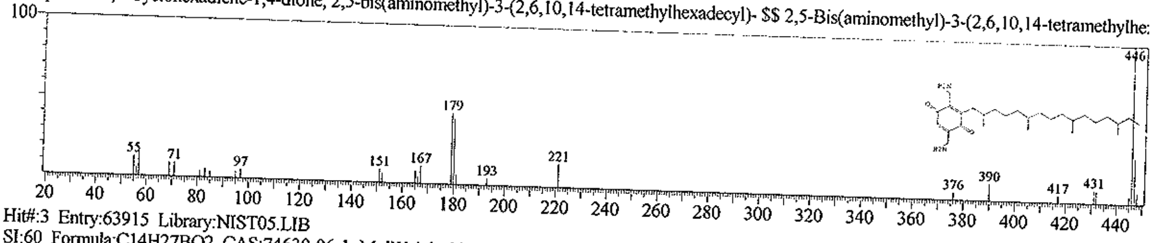

Hit#:3 Entry:63915 Library:NIST05.LIB
SI:60 Formula:C14H27BO2 CAS:74630-06-1 MolWeight:238 RetIndex:0
CompName:4H-1,3,2-Dioxaborin, 2-ethyl-4-methyl-4,6-bis(2-methylpropyl)- $$ 2-Ethyl-4,6-diisobutyl-4-methyl-4H-1,3,2-dioxaborinine # $$

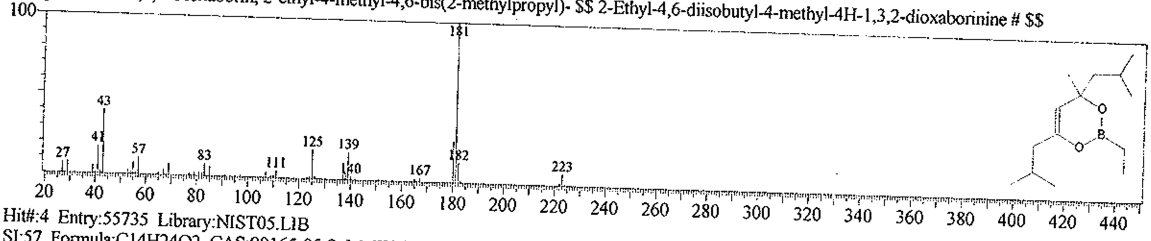

Hit#:4 Entry:55735 Library:NIST05.LIB
SI:57 Formula:C14H24O2 CAS:90165-05-2 MolWeight:224 RetIndex:1591
CompName:Ethanone, 1-[2-(5-hydroxy-1,1-dimethylhexyl)-3-methyl-2-cyclopropen-1-yl]- $$ 1-[2-(5-Hydroxy-1,1-dimethylhexyl)-3-methyl-2-cyclopropen-

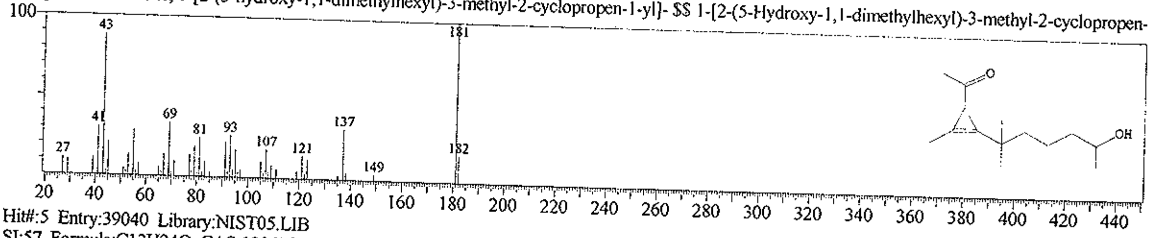

Hit#:5 Entry:39040 Library:NIST05.LIB
SI:57 Formula:C13H24O CAS:100678-94-2 MolWeight:196 RetIndex:1359
CompName:Tetrahydroedulan $$ 2,5,5,8a-Tetramethyloctahydro-2H-chromene # $$

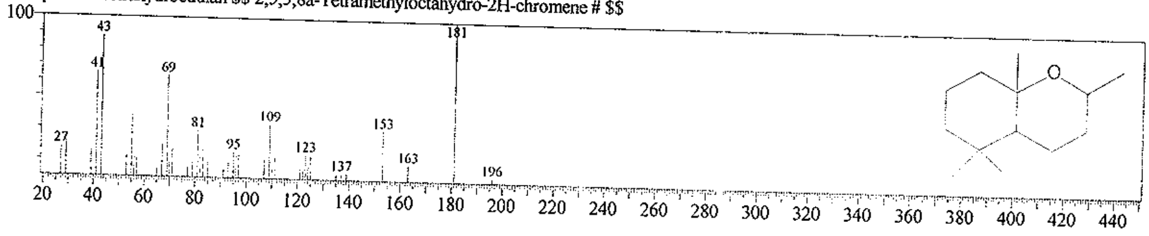

<< Target >>
Line#:12 R.Time:39.925(Scan#:4432) MassPeaks:33
RawMode:Single 39.925(4432) BasePeak:430.45(79538)
BG Mode:39.967(4437) Group 1 - Event 1

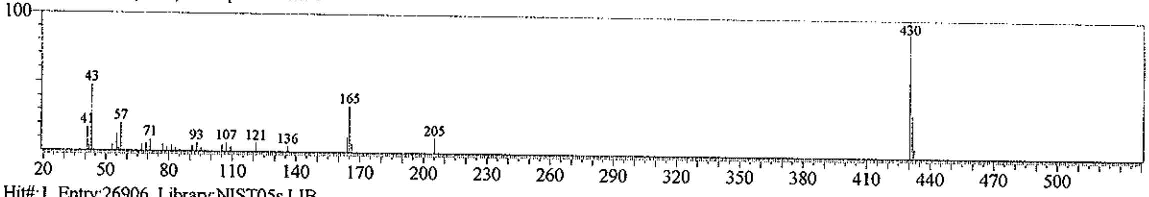

Hit#:1 Entry:26906 Library:NIST05s.LIB
SI:85 Formula:C29H50O2 CAS:10191-41-0 MolWeight:430 RetIndex:3149
CompName:Vitamin E $$ dl-.alpha.-Tocopherol $$ 2H-1-Benzopyran-6-ol, 3,4-dihydro-2,5,7,8-tetramethyl-2-(4,8,12-trimethyltridecyl)- $$ 6-Chromanol, 2

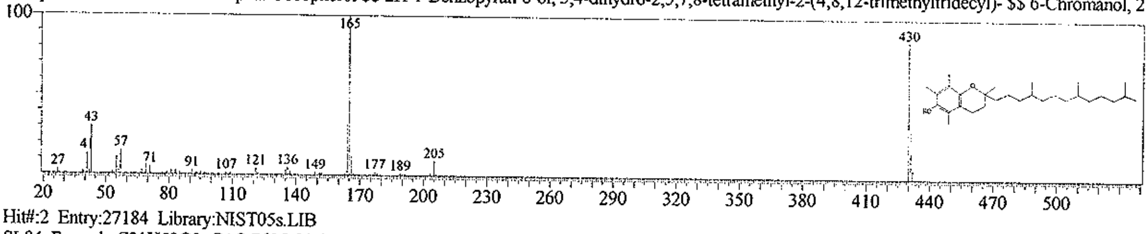

Hit#:2 Entry:27184 Library:NIST05s.LIB
SI:84 Formula:C31H52O3 CAS:7695-91-2 MolWeight:472 RetIndex:3308
CompName:Vitamin E acetate $$ dl-.alpha.-Tocopherol acetate $$ dl-.alpha.-Tocopheryl acetate $$ 2H-1-Benzopyran-6-ol, 3,4-dihydro-2,5,7,8-tetramethyl-

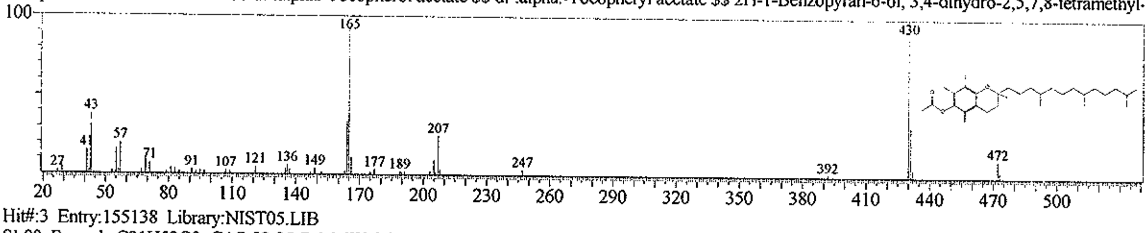

Hit#:3 Entry:155138 Library:NIST05.LIB
SI:80 Formula:C31H52O3 CAS:58-95-7 MolWeight:472 RetIndex:3308
CompName:Vitamin E acetate $$ 2H-1-Benzopyran-6-ol, 3,4-dihydro-2,5,7,8-tetramethyl-2-(4,8,12-trimethyltridecyl)-, acetate, [2R-[2R*(4R*,8R*)]]- $$ 6-

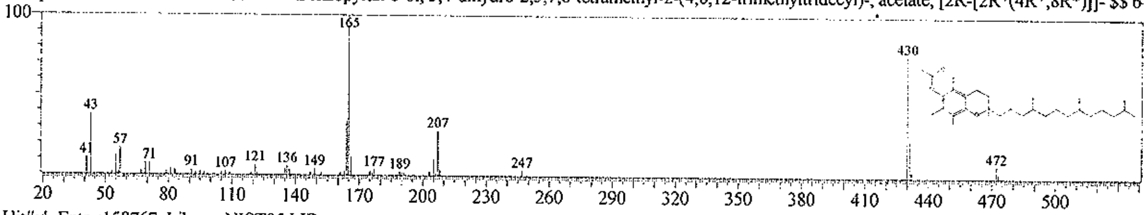

Hit#:4 Entry:158767 Library:NIST05.LIB
SI:80 Formula:C33H54O5 CAS:4345-03-3 MolWeight:530 RetIndex:3863
CompName:dl-.alpha.-Tocopherol succinate $$ Vitamin E succinate $$ D-.alpha.-Tocopherol succinate $$ D-.alpha.-Tocopheryl acid succinate $$ Butanedi

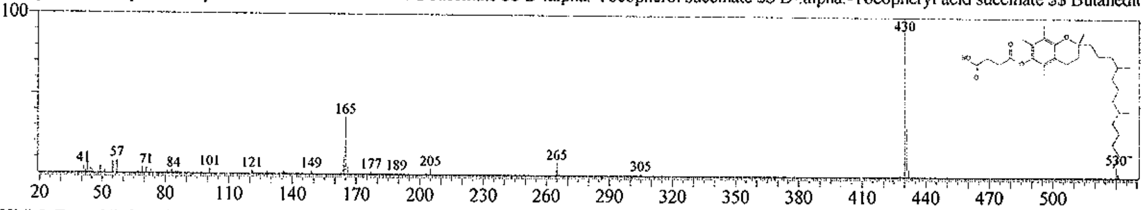

Hit#:5 Entry:27185 Library:NIST05s.LIB
SI:79 Formula:C31H52O3 CAS:58-95-7 MolWeight:472 RetIndex:3308
CompName:Vitamin E acetate $$ 2H-1-Benzopyran-6-ol, 3,4-dihydro-2,5,7,8-tetramethyl-2-(4,8,12-trimethyltridecyl)-, acetate, [2R-[2R*(4R*,8R*)]]- $$ 6-

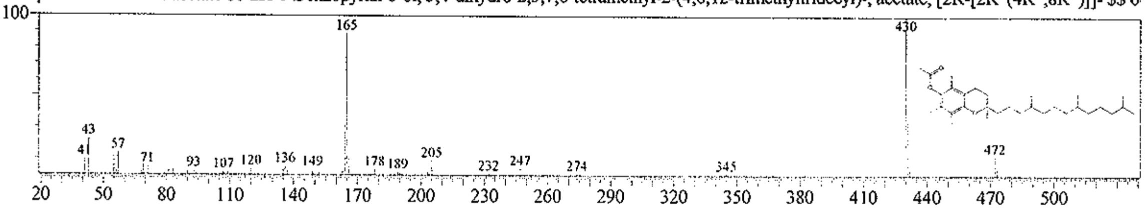

<< Target >>
Line#:13 R.Time:42.675(Scan#:4762) MassPeaks:14
RawMode:Single 42.675(4762) BasePeak:93.20(3036)
BG Mode:42.725(4768) Group 1 - Event 1

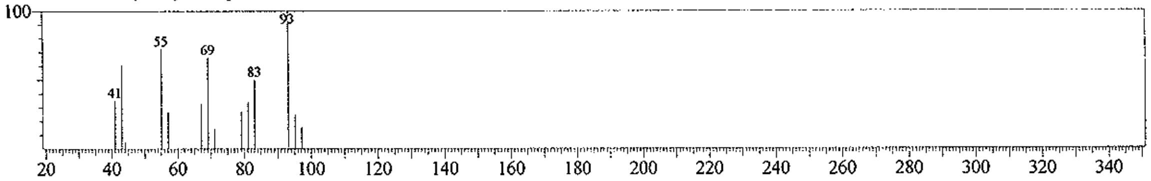

Hit#:1 Entry:24948 Library:NIST05.LIB
SI:78 Formula:C11H22O CAS:0-00-0 MolWeight:170 RetIndex:1280
CompName:2-Isopropyl-5-methylcyclohexylmethanol

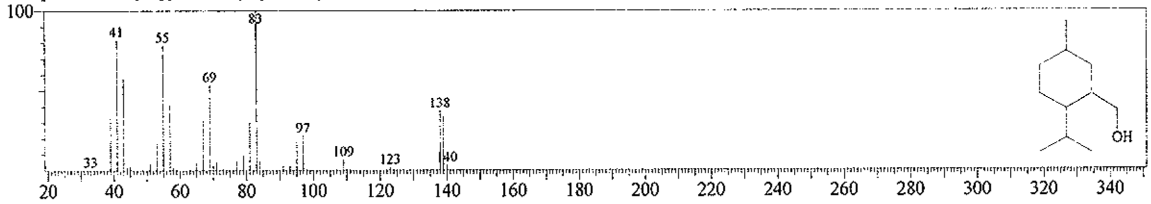

Hit#:2 Entry:39029 Library:NIST05.LIB
SI:77 Formula:C13H24O CAS:74646-37-0 MolWeight:196 RetIndex:1474
CompName:1-Tridecyn-4-ol

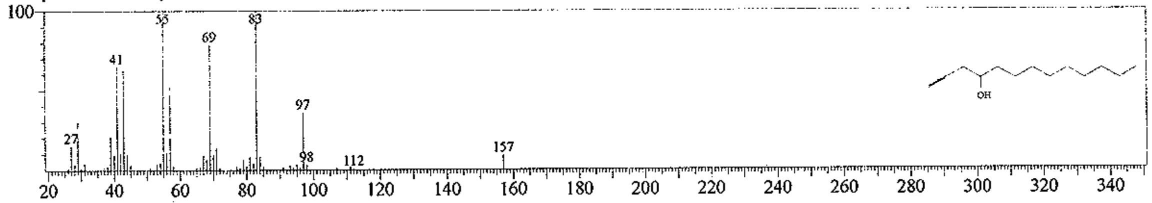

Hit#:3 Entry:31272 Library:NIST05.LIB
SI:76 Formula:C12H22O CAS:74646-36-9 MolWeight:182 RetIndex:1374
CompName:1-Dodecyn-4-ol

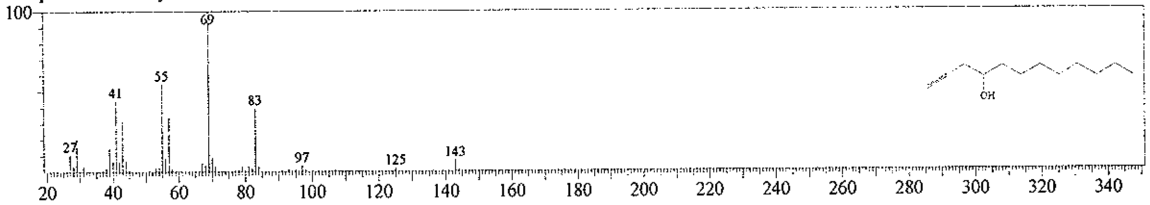

Hit#:4 Entry:48870 Library:NIST05.LIB
SI:75 Formula:C12H23NO2 CAS:118252-04-3 MolWeight:213 RetIndex:1621
CompName:1-Hexyl-2-nitrocyclohexane

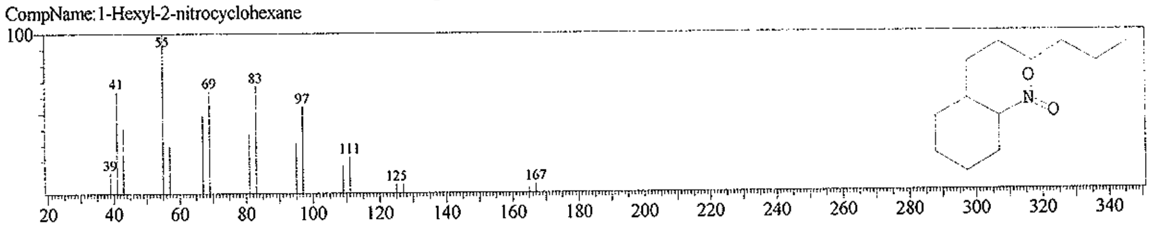

Hit#:5 Entry:124450 Library:NIST05.LIB
SI:75 Formula:C20H38ClP CAS:70971-58-3 MolWeight:344 RetIndex:2158
CompName:Phosphinous chloride, bis[5-methyl-2-(1-methylethyl)cyclohexyl]- $$ Bis(2-isopropyl-5-methylcyclohexyl)phosphinous chloride # $$

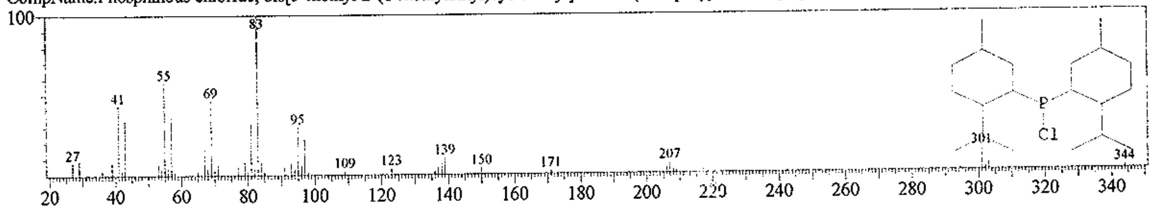

<< Target >>
Line#:14 R.Time:44.125(Scan#:4936) MassPeaks:25
RawMode:Single 44.125(4936) BasePeak:43.05(9163)
BG Mode:44.192(4944) Group 1 - Event 1

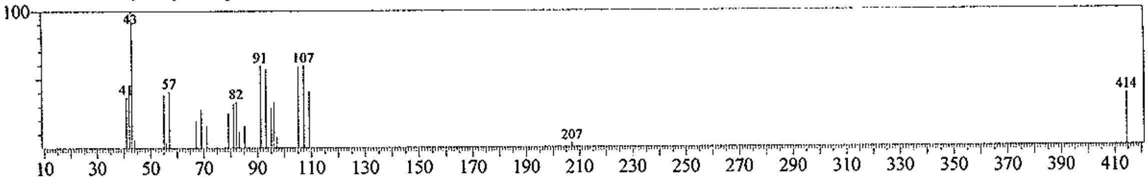

Hit#:1 Entry:27714 Library:NIST05.LIB
SI:73 Formula:C10H21Cl CAS:1002-69-3 MolWeight:176 RetIndex:1240
CompName:Decane, 1-chloro- $$ Decyl chloride $$ 1-Chlorodecane $$ n-Decyl chloride $$

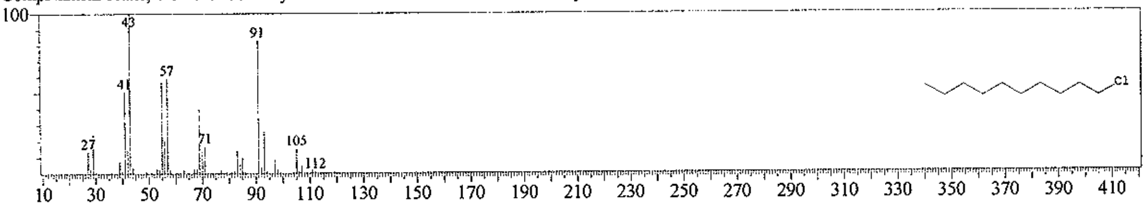

Hit#:2 Entry:20354 Library:NIST05.LIB
SI:73 Formula:C9H19Cl CAS:2473-01-0 MolWeight:162 RetIndex:1141
CompName:Nonane, 1-chloro- $$ Nonyl chloride $$ 1-Chlorononane $$ n-Nonyl chloride $$

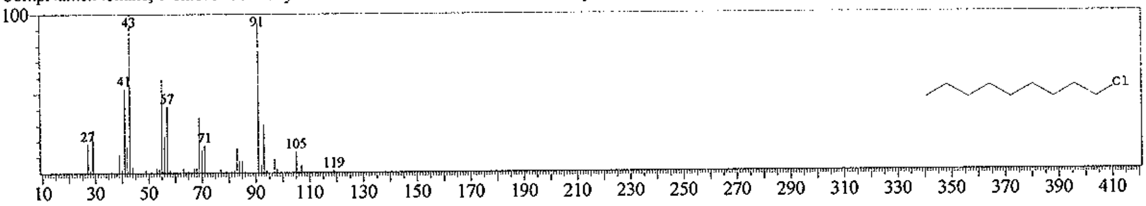

Hit#:3 Entry:16646 Library:NIST05s.LIB
SI:73 Formula:C12H25Cl CAS:112-52-7 MolWeight:204 RetIndex:1439
CompName:Dodecane, 1-chloro- $$ n-Dodecyl chloride $$ Dodecyl chloride $$ Lauryl chloride $$ 1-Chlorododecane $$

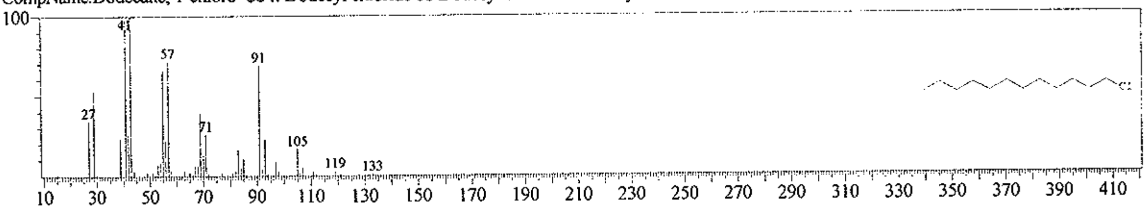

Hit#:4 Entry:53069 Library:NIST05.LIB
SI:73 Formula:C15H24O CAS:0-00-0 MolWeight:220 RetIndex:1531
CompName:trans-Z-.alpha.-Bisabolene epoxide

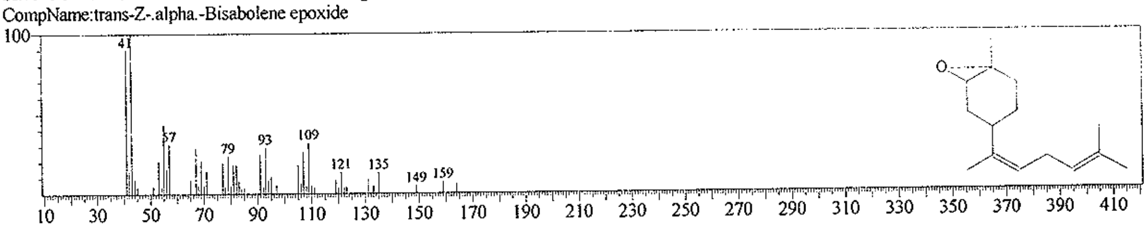

Hit#:5 Entry:12792 Library:NIST05s.LIB
SI:73 Formula:C10H21Cl CAS:1002-69-3 MolWeight:176 RetIndex:1240
CompName:Decane, 1-chloro- $$ Decyl chloride $$ 1-Chlorodecane $$ n-Decyl chloride $$

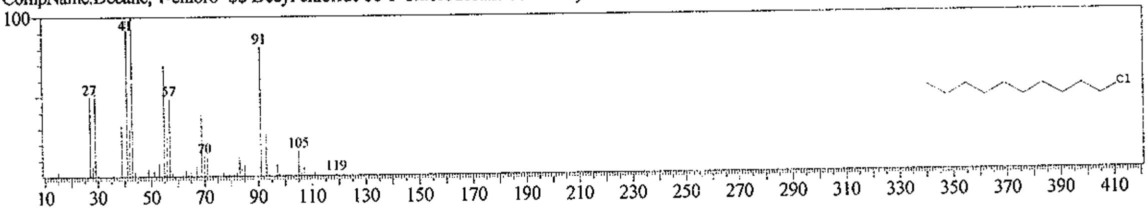

<< Target >>
Line#:15 R.Time:45.342(Scan#:5082) MassPeaks:24
RawMode:Single 45.342(5082) BasePeak:107.25(6676)
BG Mode:45.417(5091) Group 1 - Event 1

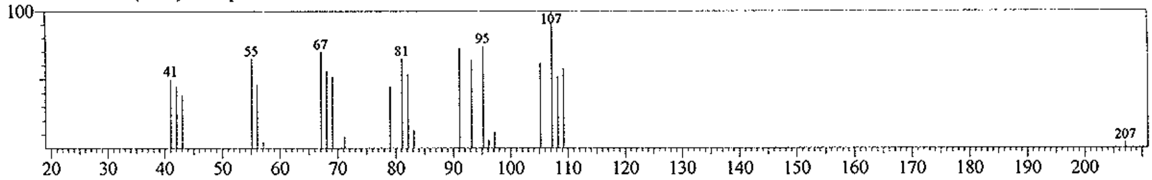

Hit#:1 Entry:71387 Library:NIST05.LIB
SI:78 Formula:C18H34 CAS:61886-64-4 MolWeight:250 RetIndex:1828
CompName:3-Octadecyne

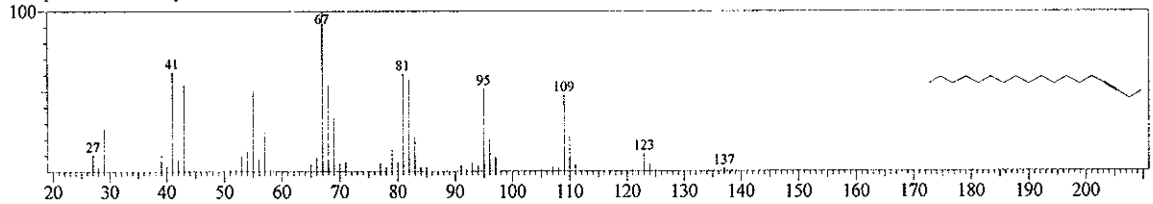

Hit#:2 Entry:22678 Library:NIST05s.LIB
SI:77 Formula:C20H38 CAS:61886-66-6 MolWeight:278 RetIndex:2027
CompName:3-Eicosyne $$ 3-Icosyne # $$

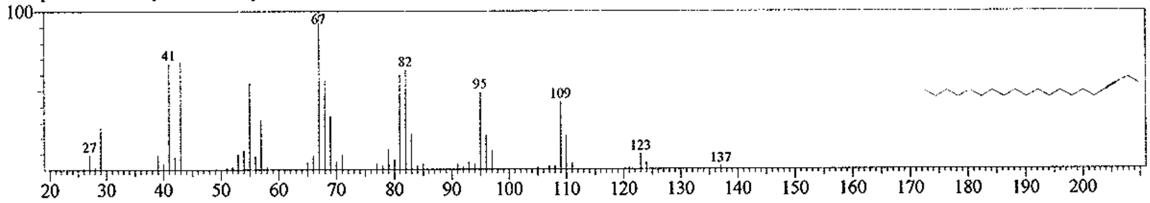

Hit#:3 Entry:15813 Library:NIST05.LIB
SI:77 Formula:C10H16O CAS:5948-04-9 MolWeight:152 RetIndex:1179
CompName:Cyclohexanone, 2-methyl-5-(1-methylethenyl)-, trans- $$ p-Menth-8-en-2-one, trans- $$ trans-Dihydrocarvone $$ Carvone, Dihydro- $$ Dihydr

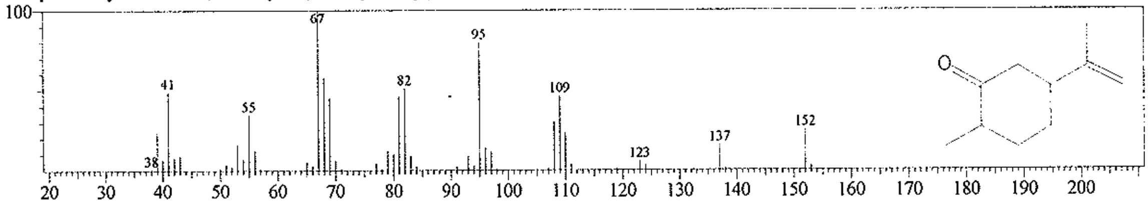

Hit#:4 Entry:8929 Library:NIST05s.LIB
SI:76 Formula:C10H16O CAS:7764-50-3 MolWeight:152 RetIndex:1179
CompName:Cyclohexanone, 2-methyl-5-(1-methylethenyl)- $$ p-Menth-8-en-2-one $$ Dihydrocarvone $$ (+)-Dihydrocarvone $$ D-Dihydrocarvone $$ 2-

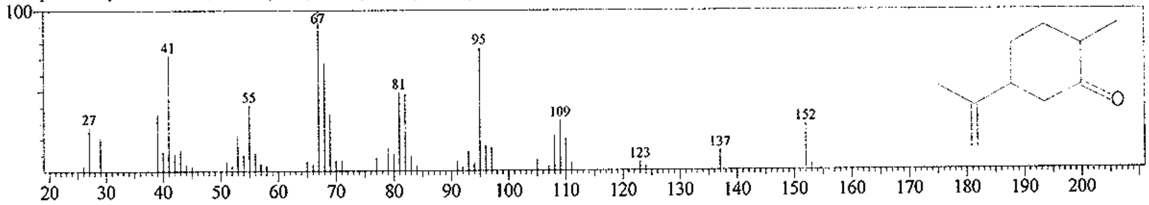

Hit#:5 Entry:55586 Library:NIST05.LIB
SI:76 Formula:C13H20O3 CAS:0-00-0 MolWeight:224 RetIndex:1487
CompName:6-Heptenoic acid, 4-isopropenyl-6-methyl-2-oxo-, ethyl ester

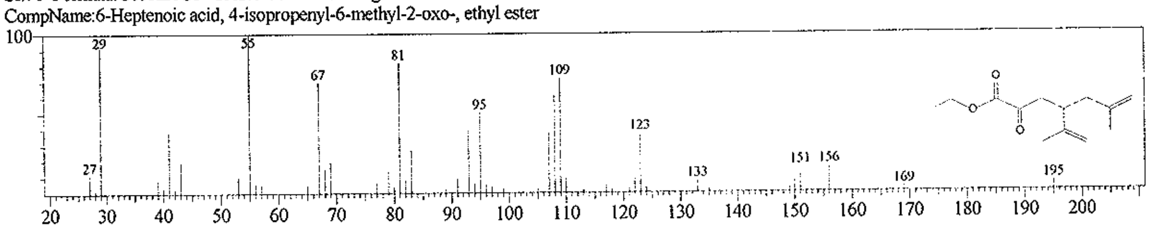

<< Target >>
Line#:16 R.Time:46.058(Scan#:5168) MassPeaks:22
RawMode:Single 46.058(5168) BasePeak:109.25(4586)
BG Mode:46.133(5177) Group 1 - Event 1

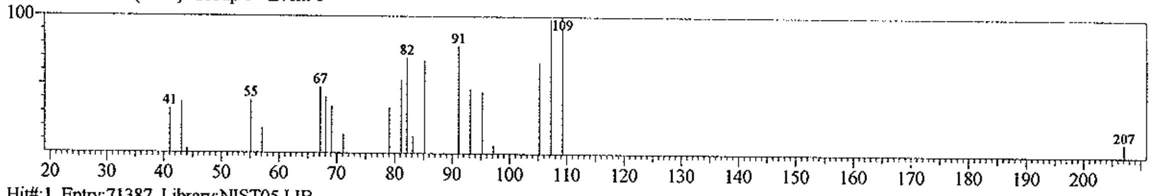

Hit#:1 Entry:71387 Library:NIST05.LIB
SI:74 Formula:C18H34 CAS:61886-64-4 MolWeight:250 RetIndex:1828
CompName:3-Octadecyne

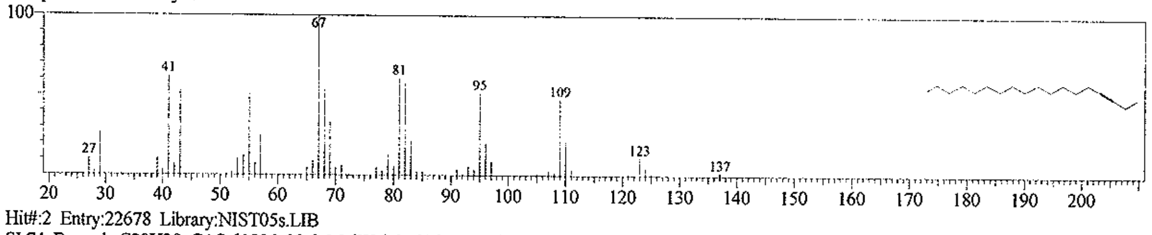

Hit#:2 Entry:22678 Library:NIST05s.LIB
SI:74 Formula:C20H38 CAS:61886-66-6 MolWeight:278 RetIndex:2027
CompName:3-Eicosyne $$ 3-Icosyne # $$

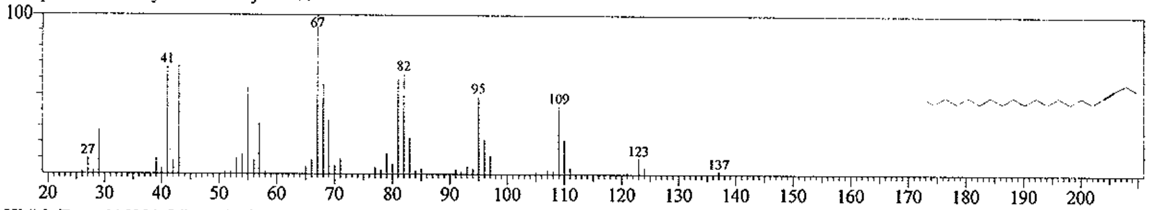

Hit#:3 Entry:115091 Library:NIST05.LIB
SI:72 Formula:C14H21Cl3O2 CAS:0-00-0 MolWeight:326 RetIndex:1985
CompName:Trichloroacetic acid, dodec-9-ynyl ester

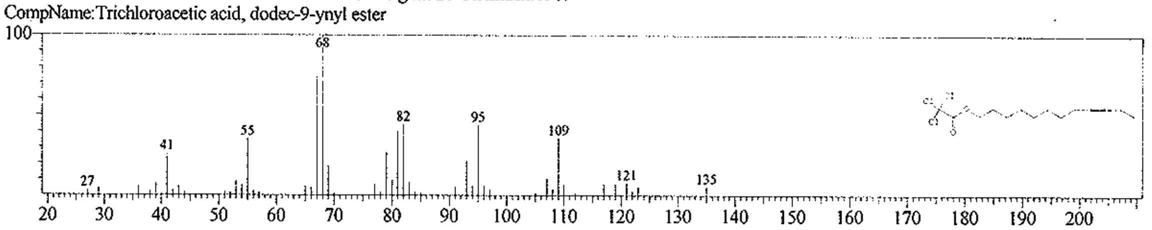

Hit#:4 Entry:54577 Library:NIST05.LIB
SI:72 Formula:C16H30 CAS:61886-62-2 MolWeight:222 RetIndex:1629
CompName:3-Hexadecyne

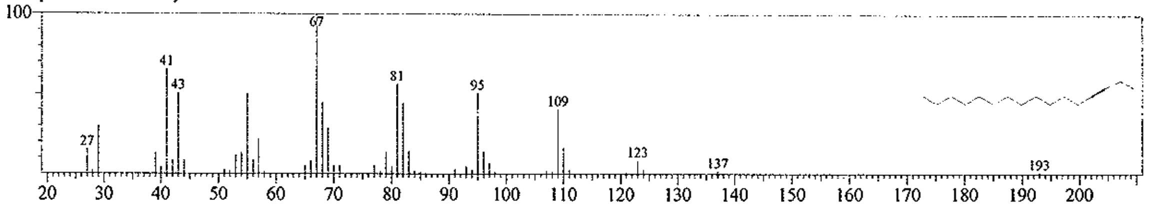

Hit#:5 Entry:15422 Library:NIST05s.LIB
SI:71 Formula:C14H26 CAS:60212-32-0 MolWeight:194 RetIndex:1430
CompName:3-Tetradecyne $$ 3-C14H26 $$

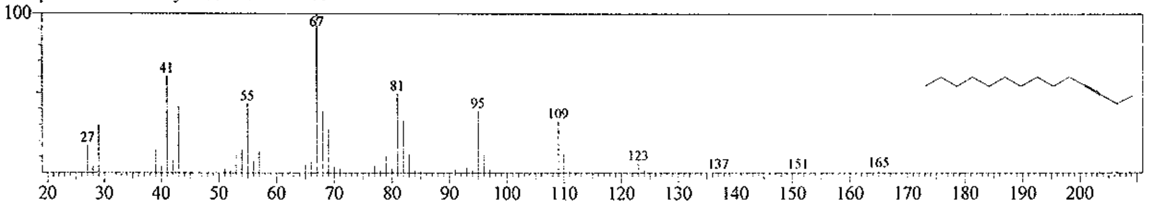

<< Target >>
Line#:17 R.Time:38.483(Scan#:4259) MassPeaks:10
RawMode:Single 38.483(4259) BasePeak:420.30(9502)
BG Mode:38.525(4264) Group 1 - Event 1

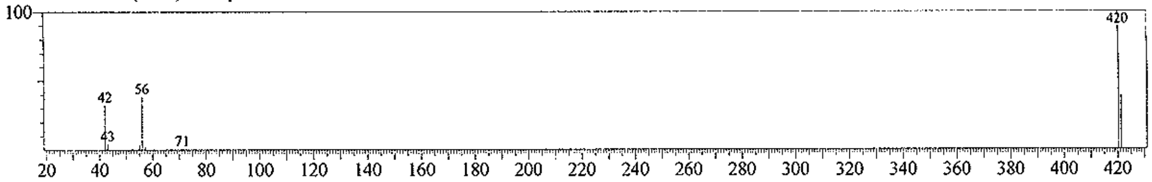

Hit#:1 Entry:148790 Library:NIST05.LIB
SI:61 Formula:C28H24N2O2 CAS:10123-03-2 MolWeight:420 RetIndex:3749
CompName:N,N'-Bis(p-methoxybenzylidene)benzidine

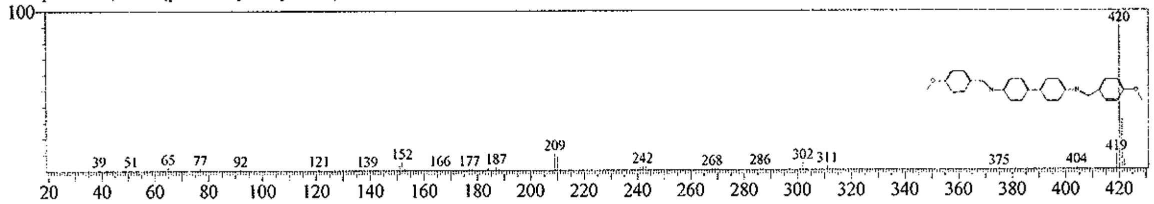

Hit#:2 Entry:148781 Library:NIST05.LIB
SI:58 Formula:C28H18F2N2 CAS:22158-34-5 MolWeight:420 RetIndex:3365
CompName:Pyrazine, 2,5-bis(p-fluorophenyl)-3,6-diphenyl- $$ 2,5-Bis(4-fluorophenyl)-3,6-diphenylpyrazine # $$

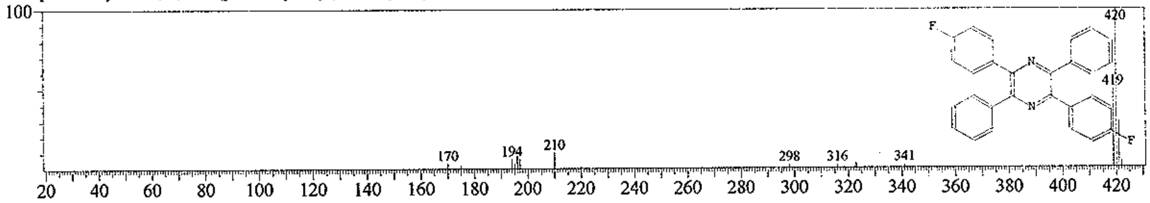

Hit#:3 Entry:148791 Library:NIST05.LIB
SI:55 Formula:C28H24N2O2 CAS:16196-93-3 MolWeight:420 RetIndex:3749
CompName:N,N'-Dibenzylidene-3,3'-dimethoxybenzidine

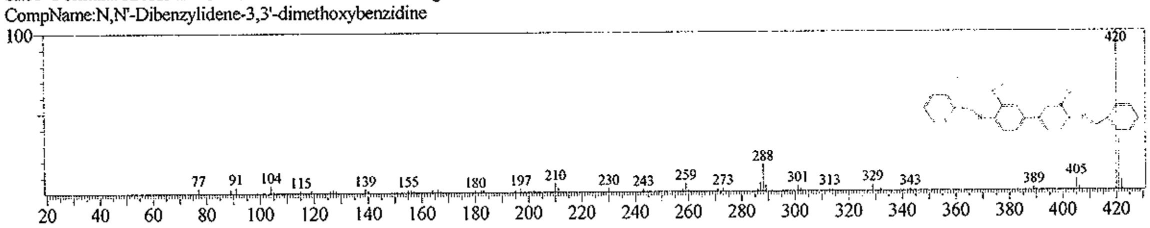

Hit#:4 Entry:148760 Library:NIST05.LIB
SI:53 Formula:C26H44O2S CAS:0-00-0 MolWeight:420 RetIndex:3100
CompName:(Phenylthio) acetic acid, octadecyl ester

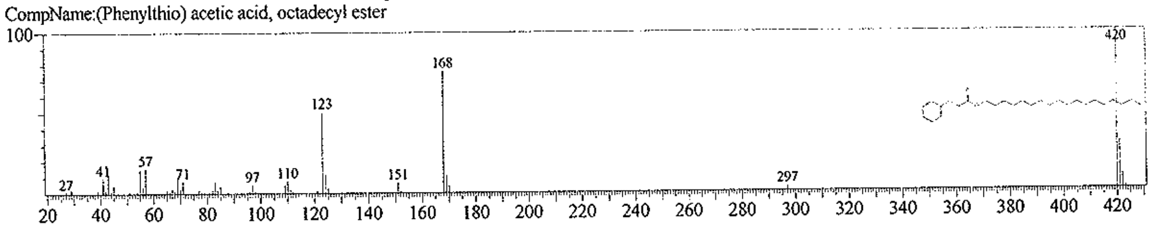

Hit#:5 Entry:148616 Library:NIST05.LIB
SI:53 Formula:C14H11ClF6N2O2S CAS:0-00-0 MolWeight:420 RetIndex:2035
CompName:4-(2-Chloro-1,1,2-trifluoro-2-trifluoromethoxy-ethylsulfanyl)-1,5-dimethyl-2-phenyl-1,2-dihydro-pyrazol-3-one

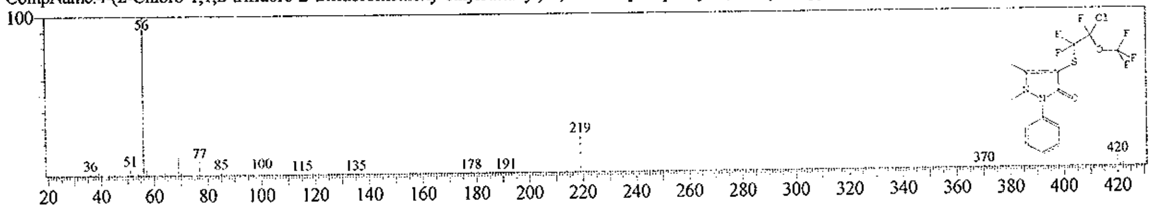

Supplement: Additional file 1 — Gas chromatography-mass spectrometry result of the leaf, stem bark and root of Ancistrocladus uncinatus. [file 1746-6148-9-120-S1.pdf]
