# Supplementary material for: Phytochemical analysis and in-vitro anti-African swine fever virus activity of extracts and fractions of Ancistrocladus uncinatus, Hutch and Dalziel (Ancistrocladaceae)
Source: BMC Vet Res. 2013 Jun 19;9:120. doi: 10.1186/1746-6148-9-120 (PMC3694037; doi:10.1186/1746-6148-9-120)

FASINA

NARICT, ZARIA
GCMS ANALYSIS

GCMS-QP2010 PLUS
SHIMADZU,JAPAN

SAMPLE - LEAVES

Chromatogram

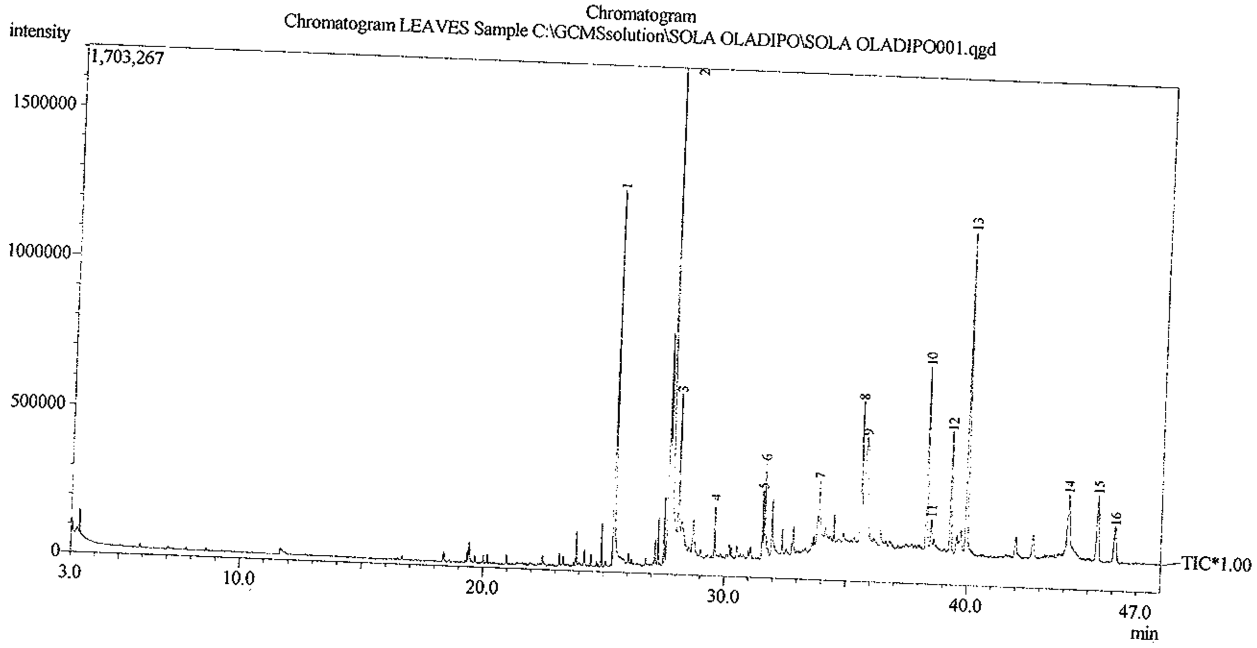

Method

[Comment]

═══ Analytical Line 1 ═══

[AOC-20i]

# of Rinses with Presolvent	:5
# of Rinses with Solvent(post)	:5
# of Rinses with Sample	:3
Plunger Speed(Suction)	:High
Viscosity Comp. Time	:0.2 sec
Plunger Speed(Injection)	:High
Syringe Insertion Speed	:High
Injection Mode	:Normal
Pumping Times	:5
Inj. Port Dwell Time	:0.3 sec
Terminal Air Gap	:No
Plunger Washing Speed	:High
Washing Volume	:8uL
Syringe Suction Position	:0.0 mm
Syringe Injection Position	:0.0 mm
Use 3 Solvent Vial	:1 vial

[GC-2010]

Column Oven Temp.	:60.0 °C
Injection Temp.	:250.00 °C
Injection Mode	:Split
Flow Control Mode	:Linear Velocity
Pressure	:100.2 kPa
Total Flow	:6.2 mL/min
Column Flow	:1.61 mL/min
Linear Velocity	:46.3 cm/sec
Purge Flow	:3.0 mL/min
Split Ratio	:1.0
High Pressure Injection	:OFF
Carrier Gas Saver	:OFF
Splitter Hold	:OFF

Oven Temp. Program

Rate	Temperature(°C)	Hold Time(min)
-	60.0	3.00
7.00	140.0	0.00
7.00	280.0	15.00

< Ready Check Heat Unit >
Column Oven : Yes
SPL2 : Yes
MS : Yes
< Ready Check Detector(FTD) >
< Ready Check Baseline Drift >
< Ready Check Injection Flow >
SPL2 Carrier : Yes
SPL2 Purge : Yes
< Ready Check APC Flow >
< Ready Check Detector APC Flow >
External Wait :No
Equilibrium Time :3.0 min

[GC Program]

[GCMS-QP2010 Plus]
IonSourceTemp :200.00 °C
Interface Temp. :250.00 °C
Solvent Cut Time :2.50 min
Detector Gain Mode :Relative
Detector Gain :0.00 kV
Threshold :3000

[MS Table]
--Group 1 - Event 1--
Start Time :3.00min
End Time :48.00min
ACQ Mode :Scan
Event Time :0.50sec
Scan Speed :1666
Start m/z :40.00
End m/z :800.00

Sample Inlet Unit :GC

[MS Program]
Use MS Program :OFF

Spectrum

Line#:1 R.Time:25.4(Scan#:2691)
MassPeaks:52
RawMode:Single 25.4(2691) BasePeak:43(128740)
BG Mode:25.5(2697) Group 1 - Event 1

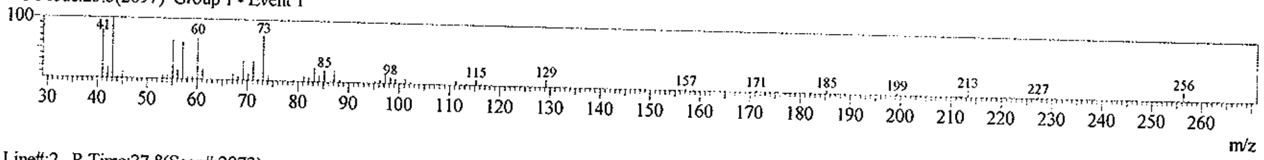

Line#:2 R.Time:27.8(Scan#:2973)
MassPeaks:57
RawMode:Single 27.8(2973) BasePeak:55(134429)
BG Mode:27.7(2966) Group 1 - Event 1

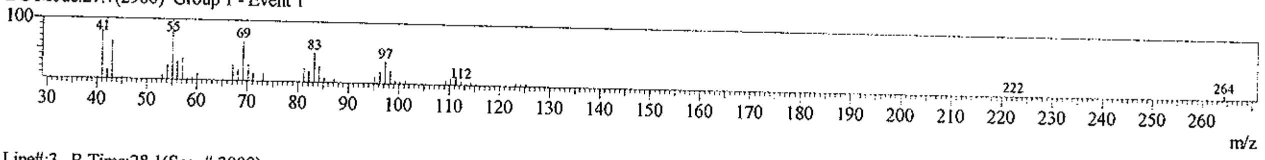

Line#:3 R.Time:28.1(Scan#:3009)
MassPeaks:44
RawMode:Single 28.1(3009) BasePeak:43(40240)
BG Mode:28.1(3013) Group 1 - Event 1

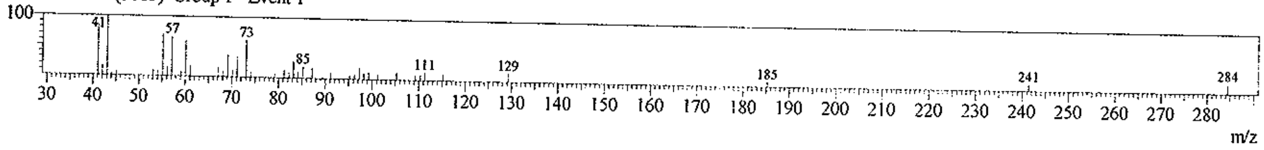

Line#:4 R.Time:29.6(Scan#:3191)
MassPeaks:28
RawMode:Single 29.6(3191) BasePeak:57(16235)
BG Mode:29.6(3195) Group 1 - Event 1

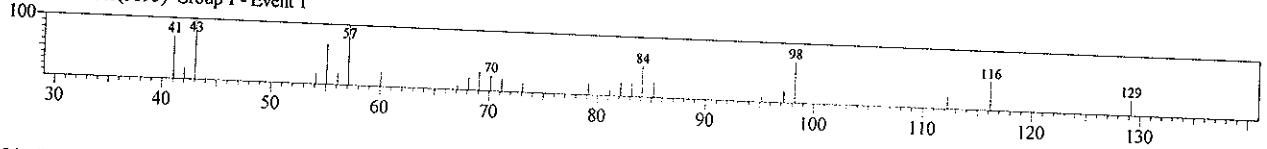

Line#:5 R.Time:31.6(Scan#:3428)
MassPeaks:31
RawMode:Single 31.6(3428) BasePeak:67(16352)
BG Mode:31.6(3431) Group 1 - Event 1

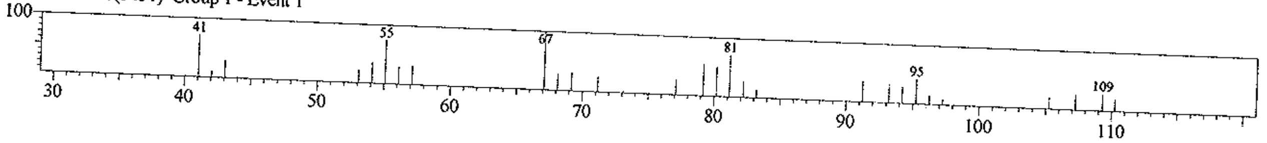

Line#:6 R.Time:31.6(Scan#:3437)
MassPeaks:37
RawMode:Single 31.6(3437) BasePeak:55(23485)
BG Mode:31.7(3441) Group 1 - Event 1

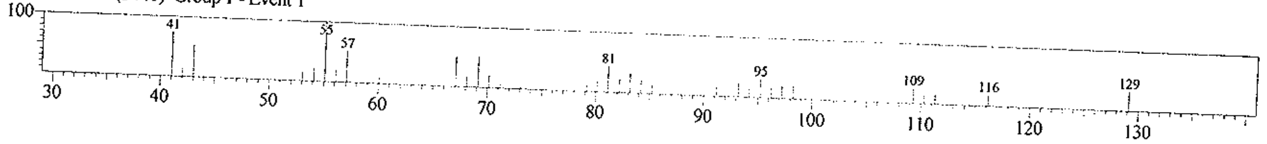

Line#:7 R.Time:33.9(Scan#:3706)
MassPeaks:36
RawMode:Single 33.9(3706) BasePeak:55(7476)
BG Mode:33.9(3709) Group 1 - Event 1

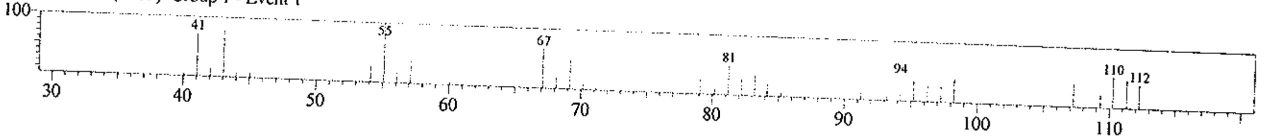

Line#:8 R.Time:35.6(Scan#:3911)
MassPeaks:32
RawMode:Single 35.6(3911) BasePeak:69(109865)
BG Mode:35.6(3916) Group 1 - Event 1

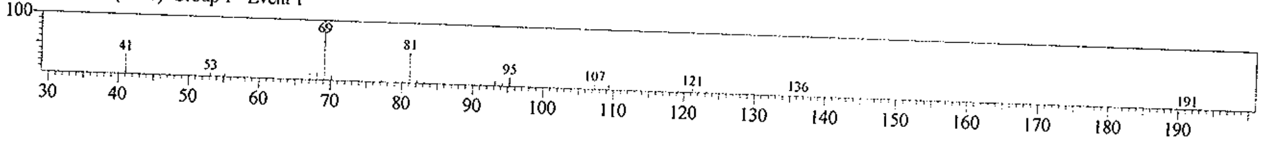

Line#:9 R.Time:35.8(Scan#:3935)
MassPeaks:39
RawMode:Single 35.8(3935) BasePeak:43(22486)
BG Mode:35.7(3927) Group 1 - Event 1

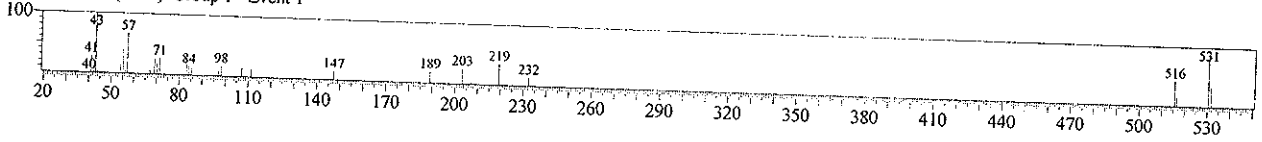

Line#:10 R.Time:38.3(Scan#:4237)
MassPeaks:44
RawMode:Single 38.3(4237) BasePeak:416(111996)
BG Mode:38.4(4243) Group 1 - Event 1

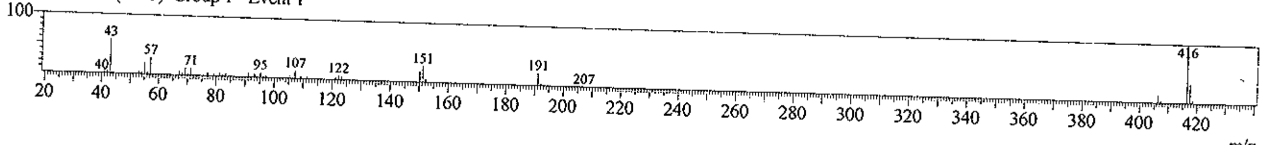

Line#:11 R.Time:38.5(Scan#:4260)
MassPeaks:11
RawMode:Single 38.5(4260) BasePeak:420(30350)
BG Mode:38.5(4264) Group 1 - Event 1

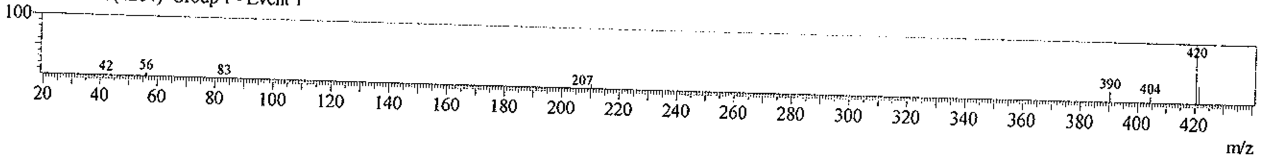

Line#:12 R.Time:39.3(Scan#:4355)
MassPeaks:39
RawMode:Single 39.3(4355) BasePeak:181(48235)
BG Mode:39.3(4351) Group 1 - Event 1

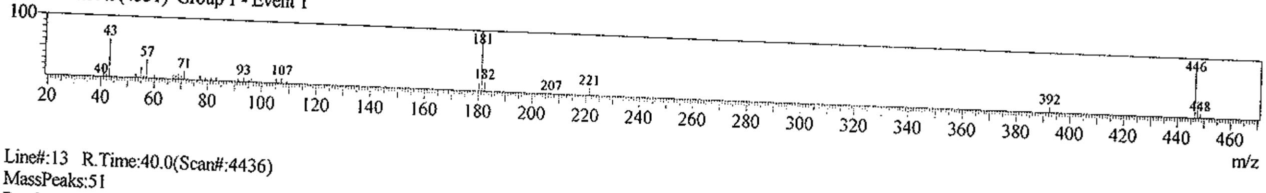

Line#:13 R.Time:40.0(Scan#:4436)
MassPeaks:51
RawMode:Single 40.0(4436) BasePeak:430(174063)
BG Mode:39.9(4429) Group 1 - Event 1

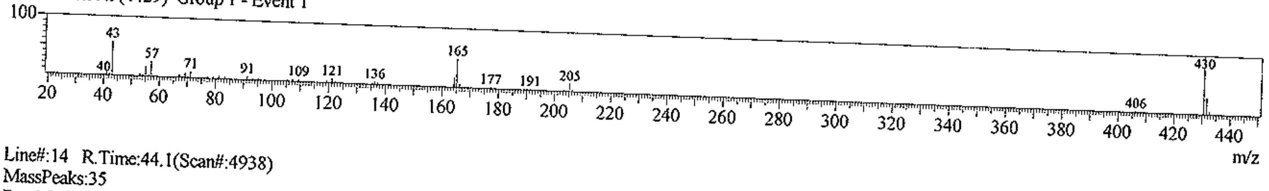

Line#:14 R.Time:44.1(Scan#:4938)
MassPeaks:35
RawMode:Single 44.1(4938) BasePeak:43(15071)
BG Mode:44.2(4947) Group 1 - Event 1

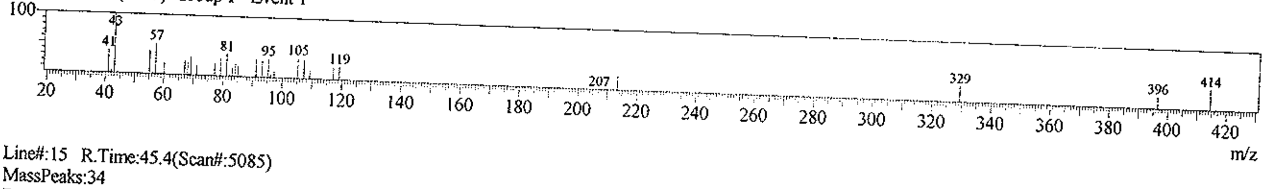

Line#:15 R.Time:45.4(Scan#:5085)
MassPeaks:34
RawMode:Single 45.4(5085) BasePeak:95(10581)
BG Mode:45.3(5076) Group 1 - Event 1

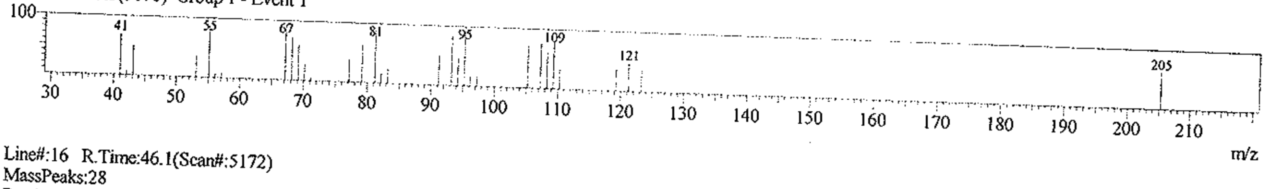

Line#:16 R.Time:46.1(Scan#:5172)
MassPeaks:28
RawMode:Single 46.1(5172) BasePeak:91(5470)
BG Mode:46.0(5163) Group 1 - Event 1

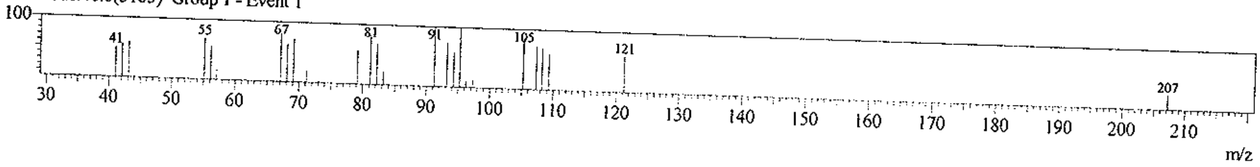

Spectrum Comparison

Library

<< Target >>
Line#:1 R.Time:25.417(Scan#:2691) MassPeaks:52
RawMode:Single 25.417(2691) BasePeak:43.05(128740)
BG Mode:25.467(2697) Group 1 - Event 1

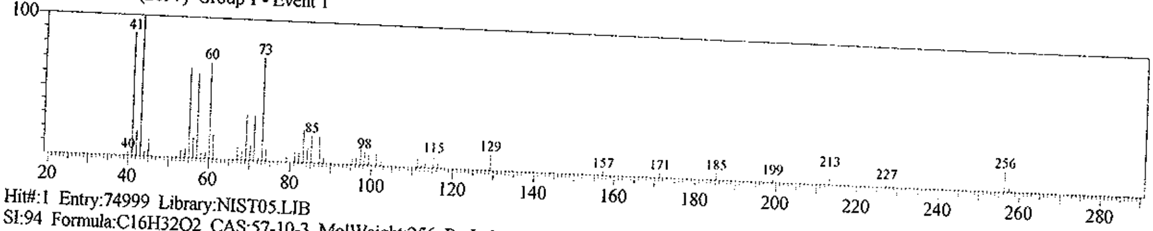

Hit#:1 Entry:74999 Library:NIST05.LIB
SI:94 Formula:C16H32O2 CAS:57-10-3 MolWeight:256 RetIndex:1968
CompName:n-Hexadecanoic acid $$ Hexadecanoic acid $$ n-Hexadecoic acid $$ Palmitic acid $$ Pentadecanecarboxylic acid $$ 1-Pentadecanecarboxylic

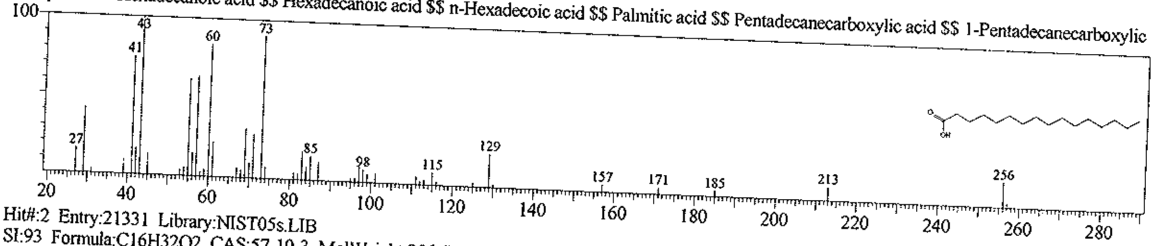

Hit#:2 Entry:21331 Library:NIST05s.LIB
SI:93 Formula:C16H32O2 CAS:57-10-3 MolWeight:256 RetIndex:1968
CompName:n-Hexadecanoic acid $$ Hexadecanoic acid $$ n-Hexadecoic acid $$ Palmitic acid $$ Pentadecanecarboxylic acid $$ 1-Pentadecanecarboxylic

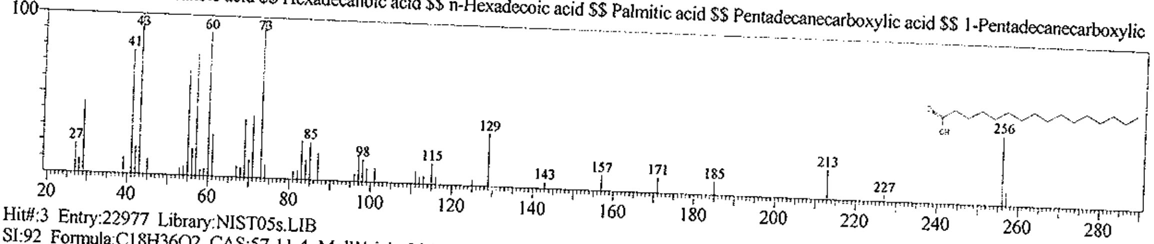

Hit#:3 Entry:22977 Library:NIST05s.LIB
SI:92 Formula:C18H36O2 CAS:57-11-4 MolWeight:284 RetIndex:2167
CompName:Octadecanoic acid $$ Stearic acid $$ n-Octadecanoic acid $$ Humko Industrene R $$ Hydrofol Acid 150 $$ Hystrene S-97 $$ Hystrene T-70 $

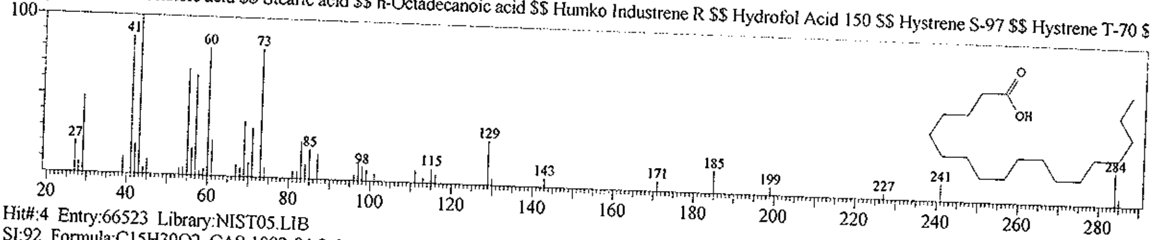

Hit#:4 Entry:66523 Library:NIST05.LIB
SI:92 Formula:C15H30O2 CAS:1002-84-2 MolWeight:242 RetIndex:1869
CompName:Pentadecanoic acid $$ Pentadecylic acid $$ n-Pentadecanoic acid $$ n-Pentadecylic acid $$

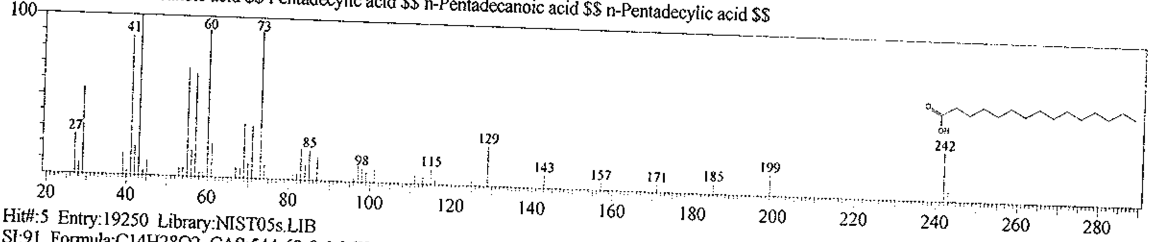

Hit#:5 Entry:19250 Library:NIST05s.LIB
SI:91 Formula:C14H28O2 CAS:544-63-8 MolWeight:228 RetIndex:1769
CompName:Tetradecanoic acid $$ Myristic acid $$ n-Tetradecanoic acid $$ n-Tetradecoic acid $$ Neo-Fat 14 $$ Univol U 316S $$ 1-Tridecanecarboxylic

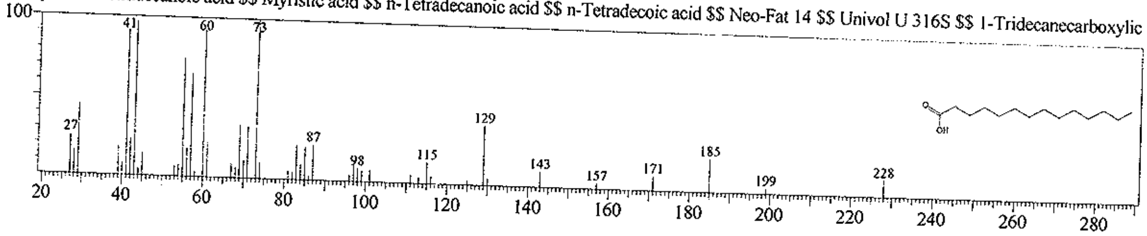

<< Target >>
Line#:2 R.Time:27.767(Scan#:2973) MassPeaks:57
RawMode:Single 27.767(2973) BasePeak:55.10(134429)
BG Mode:27.708(2966) Group 1 - Event 1

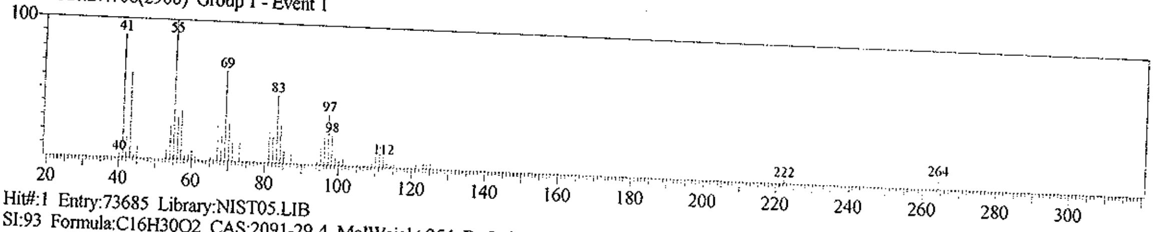

Hit#:1 Entry:73685 Library:NIST05.LIB
SI:93 Formula:C16H30O2 CAS:2091-29-4 MolWeight:254 RetIndex:1976
CompName:9-Hexadecenoic acid $$ (9E)-9-Hexadecenoic acid # $$

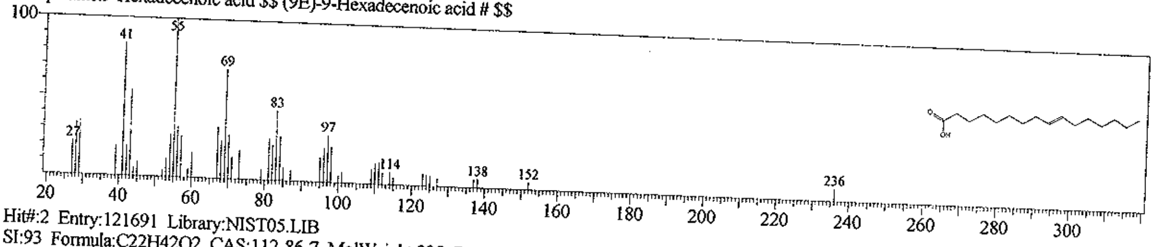

Hit#:2 Entry:121691 Library:NIST05.LIB
SI:93 Formula:C22H42O2 CAS:112-86-7 MolWeight:338 RetIndex:2572
CompName:Erucic acid $$ 13-Docosenoic acid, (Z)- $$ .delta.13-cis-Docosenoic acid $$ cis-13-Docosenoic acid $$ (Z)-13-Docosenoic acid $$ Prifrac 299

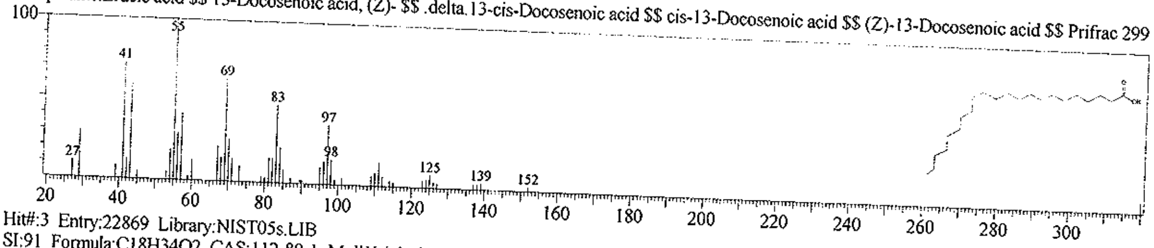

Hit#:3 Entry:22869 Library:NIST05s.LIB
SI:91 Formula:C18H34O2 CAS:112-80-1 MolWeight:282 RetIndex:2175
CompName:Oleic Acid $$ 9-Octadecenoic acid (Z)- $$ .delta.(Sup9)-cis-Oleic acid $$ cis-.delta.(Sup9)-Octadecenoic acid $$ cis-Oleic Acid $$ cis-9-Octad

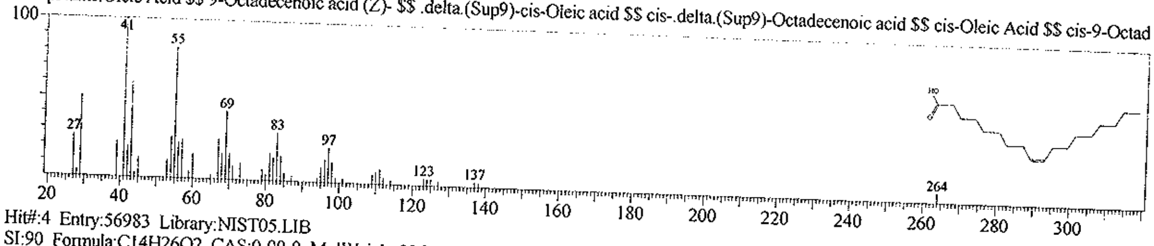

Hit#:4 Entry:56983 Library:NIST05.LIB
SI:90 Formula:C14H26O2 CAS:0-00-0 MolWeight:226 RetIndex:1777
CompName:Z-11-Tetradecenoic acid

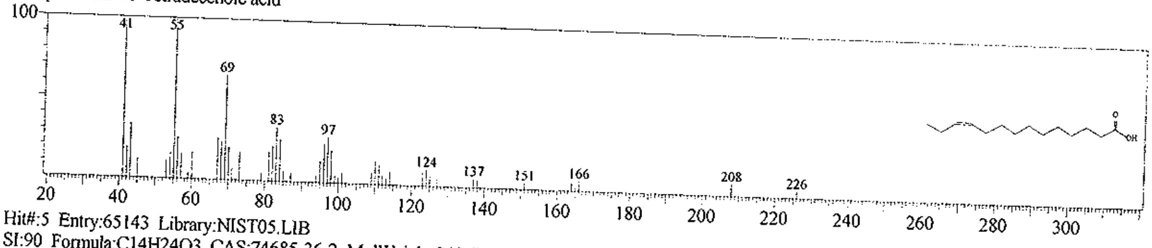

Hit#:5 Entry:65143 Library:NIST05.LIB
SI:90 Formula:C14H24O3 CAS:74685-36-2 MolWeight:240 RetIndex:2137
CompName:Oxacyclotetradecane-2,11-dione, 13-methyl- $$ 13-Methyloxacyclotetradecane-2,11-dione # $$

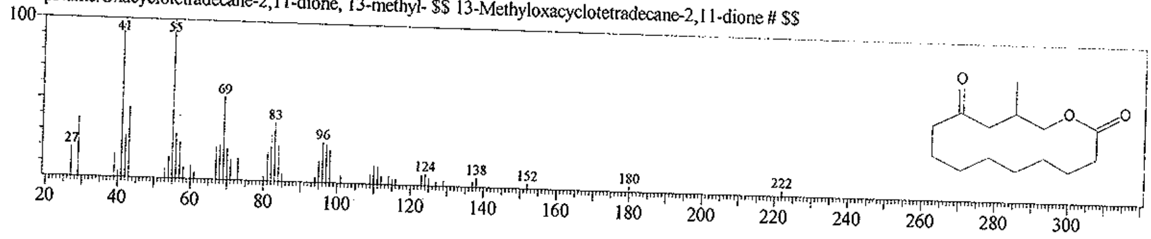

<< Target >>
Line#:3 R.Time:28.067(Scan#:3009) MassPeaks:44
RawMode:Single 28.067(3009) BasePeak:43.10(40240)
BG Mode:28.100(3013) Group 1 - Event 1

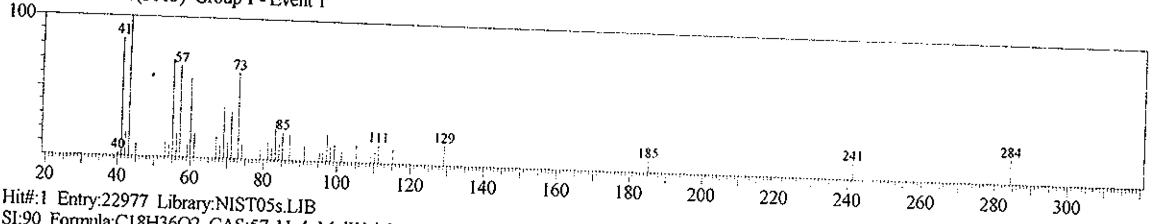

Hit#:1 Entry:22977 Library:NIST05s.LIB
SI:90 Formula:C18H36O2 CAS:57-11-4 MolWeight:284 RetIndex:2167
CompName:Octadecanoic acid $$ Stearic acid $$ n-Octadecanoic acid $$ Humko Industrene R $$ Hydrofol Acid 150 $$ Hystrene S-97 $$ Hystrene T-70 $

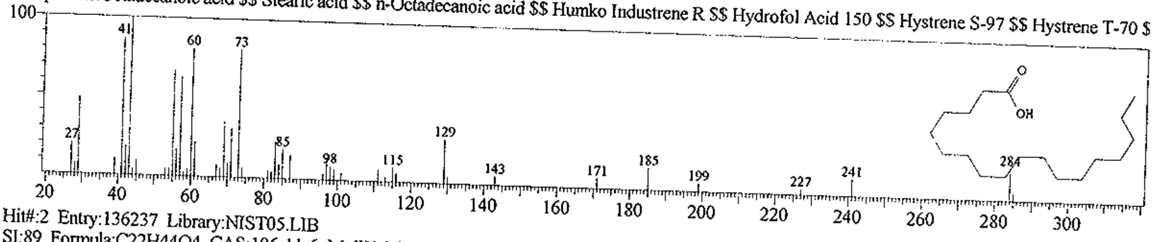

Hit#:2 Entry:136237 Library:NIST05.LIB
SI:89 Formula:C22H44O4 CAS:106-11-6 MolWeight:372 RetIndex:2694
CompName:Octadecanoic acid, 2-(2-hydroxyethoxy)ethyl ester $$ Aqua Cera $$ Atlas G 2146 $$ Cerasynt $$ Cerasynt Special $$ Clindrol SDG $$ Diethy

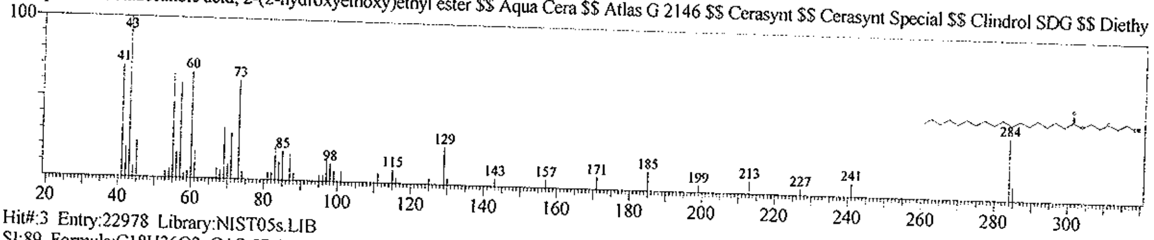

Hit#:3 Entry:22978 Library:NIST05s.LIB
SI:89 Formula:C18H36O2 CAS:57-11-4 MolWeight:284 RetIndex:2167
CompName:Octadecanoic acid $$ Stearic acid $$ n-Octadecanoic acid $$ Humko Industrene R $$ Hydrofol Acid 150 $$ Hystrene S-97 $$ Hystrene T-70 $

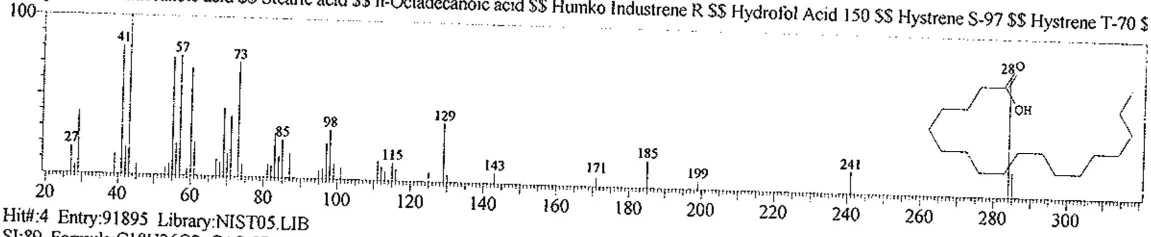

Hit#:4 Entry:91895 Library:NIST05.LIB
SI:89 Formula:C18H36O2 CAS:57-11-4 MolWeight:284 RetIndex:2167
CompName:Octadecanoic acid $$ Stearic acid $$ n-Octadecanoic acid $$ Humko Industrene R $$ Hydrofol Acid 150 $$ Hystrene S-97 $$ Hystrene T-70 $

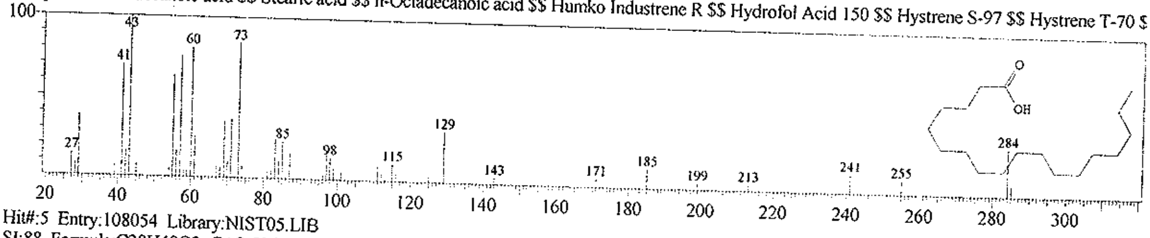

Hit#:5 Entry:108054 Library:NIST05.LIB
SI:88 Formula:C20H40O2 CAS:506-30-9 MolWeight:312 RetIndex:2366
CompName:Eicosanoic acid $$ Arachic acid $$ Arachidic acid $$ Icosanoic acid $$ n-Eicosanoic acid $$ Arachidic acid (synthetic) $$

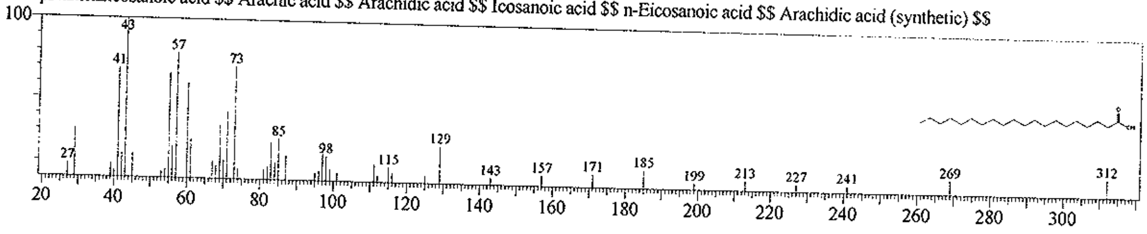

<< Target >>
Line#:4 R.Time:29.583(Scan#:3191) MassPeaks:28
RawMode:Single 29.583(3191) BasePeak:57.10(16235)
BG Mode:29.617(3195) Group 1 - Event 1

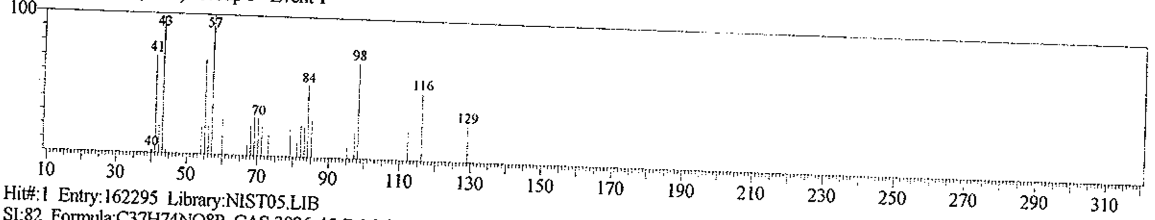

Hit#:1 Entry:162295 Library:NIST05.LIB
SI:82 Formula:C37H74NO8P CAS:3026-45-7 MolWeight:691 RetIndex:0
CompName:Hexadecanoic acid, 1-[[[(2-aminoethoxy)hydroxyphosphinyl]oxy]methyl]-1,2-ethanediyl ester $$ Palmitin, 1,2-di-, 2-aminoethyl hydrogen phos

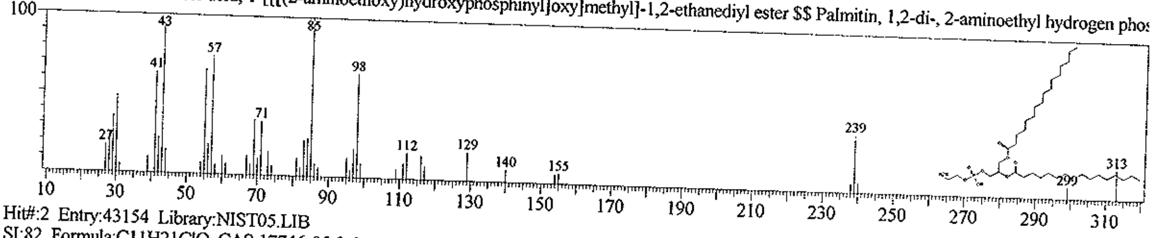

Hit#:2 Entry:43154 Library:NIST05.LIB
SI:82 Formula:C11H21ClO CAS:17746-05-3 MolWeight:204 RetIndex:1427
CompName:Undecanoyl chloride $$ n-Undecanoyl chloride $$

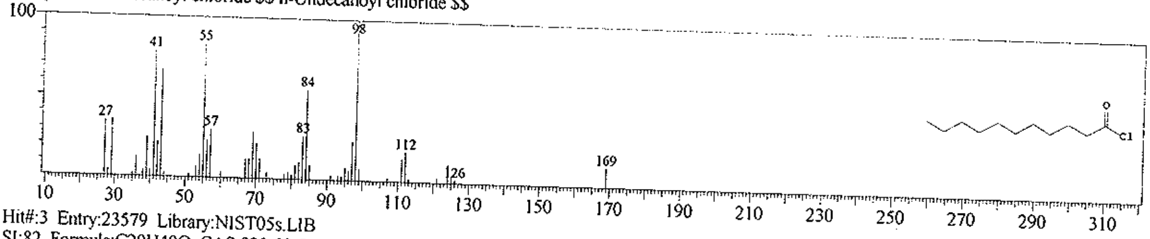

Hit#:3 Entry:23579 Library:NIST05s.LIB
SI:82 Formula:C20H40O CAS:930-02-9 MolWeight:296 RetIndex:2075
CompName:Octadecane, 1-(ethenyloxy)- $$ Ether, octadecyl vinyl $$ Octadecyl vinyl ether $$ Stearyl vinyl ether $$ Vinyl stearyl ether $$ Vinyl octadecyl

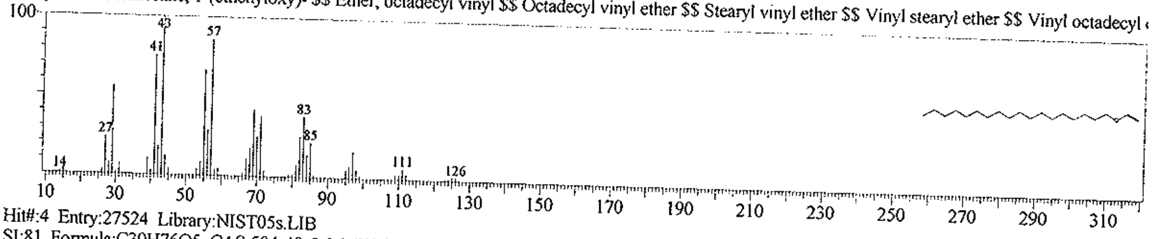

Hit#:4 Entry:27524 Library:NIST05s.LIB
SI:81 Formula:C39H76O5 CAS:504-40-5 MolWeight:624 RetIndex:4395
CompName:Octadecanoic acid, 2-hydroxy-1,3-propanediyl ester $$ Stearin, 1,3-di- $$ Glycerin 1,3-distearate $$ Glyceryl 1,3-distearate $$ Stearic acid digl

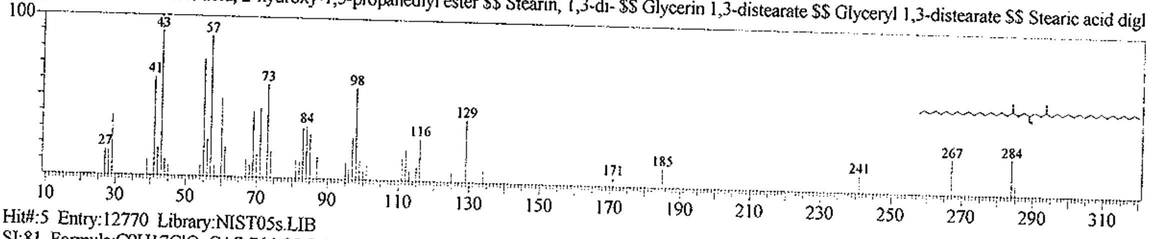

Hit#:5 Entry:12770 Library:NIST05s.LIB
SI:81 Formula:C9H17ClO CAS:764-85-2 MolWeight:176 RetIndex:1228
CompName:Nonanoyl chloride $$ Pelargonoyl chloride $$ Nonanoic acid chloride $$

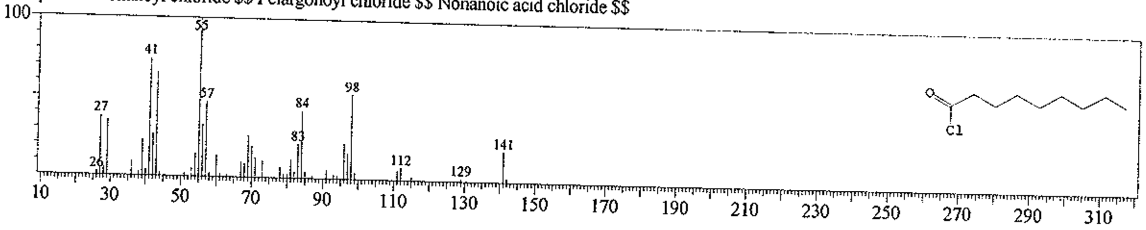

<< Target >>
Line#:5 R.Time:31.558(Scan#:3428) MassPeaks:31
RawMode:Single 31.558(3428) BasePeak:67.10(16352)
BG Mode:31.583(3431) Group 1 - Event 1

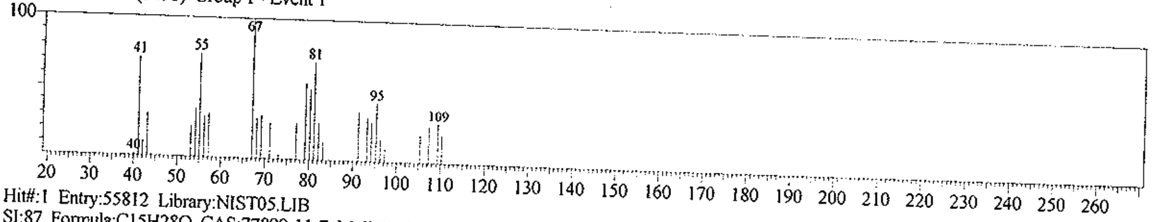

Hit#:1 Entry:55812 Library:NIST05.LIB
SI:87 Formula:C15H28O CAS:77899-11-7 MolWeight:224 RetIndex:1771
CompName:(Z)6,(Z)9-Pentadecadien-1-ol $$ (6Z,9Z)-6,9-Pentadecadien-1-ol # $$

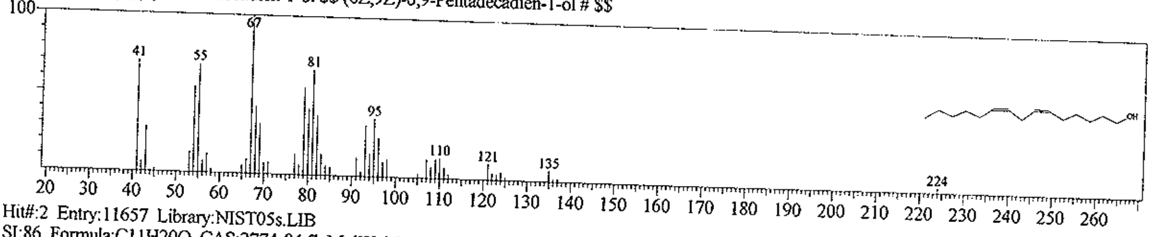

Hit#:2 Entry:11657 Library:NIST05s.LIB
SI:86 Formula:C11H20O CAS:2774-84-7 MolWeight:168 RetIndex:1355
CompName:10-Undecyn-1-ol

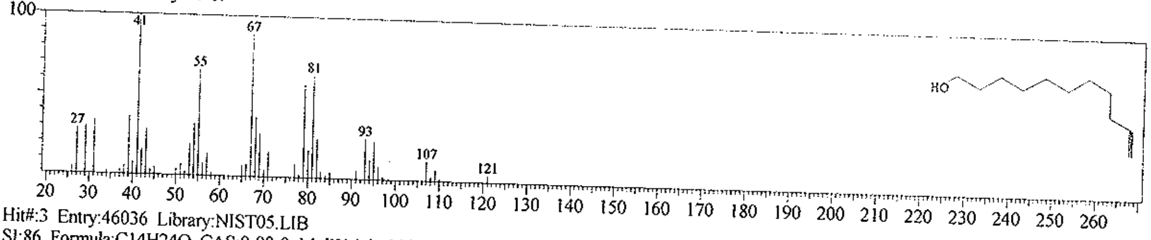

Hit#:3 Entry:46036 Library:NIST05.LIB
SI:86 Formula:C14H24O CAS:0-00-0 MolWeight:208 RetIndex:1663
CompName:13-Tetradece-11-yn-1-ol

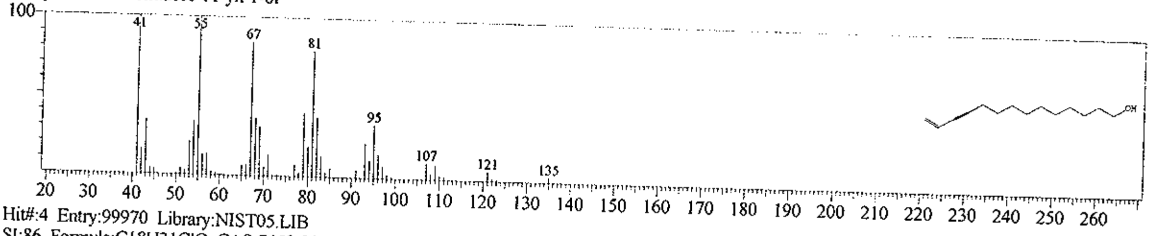

Hit#:4 Entry:99970 Library:NIST05.LIB
SI:86 Formula:C18H31ClO CAS:7459-33-8 MolWeight:298 RetIndex:2139
CompName:9,12-Octadecadienoyl chloride, (Z,Z)- $$ Linoleoyl chloride $$ Lineoleoyl chloride $$ Linoleic acid chloride $$ (9E,12E)-9,12-Octadecadienoy

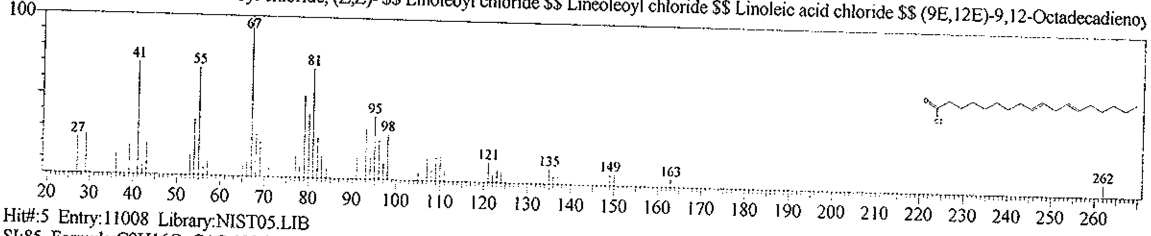

Hit#:5 Entry:11008 Library:NIST05.LIB
SI:85 Formula:C9H16O CAS:13366-81-9 MolWeight:140 RetIndex:1245
CompName:4-Cyclooctene-1-methanol $$ 4-Cycloocten-1-ylmethanol # $$

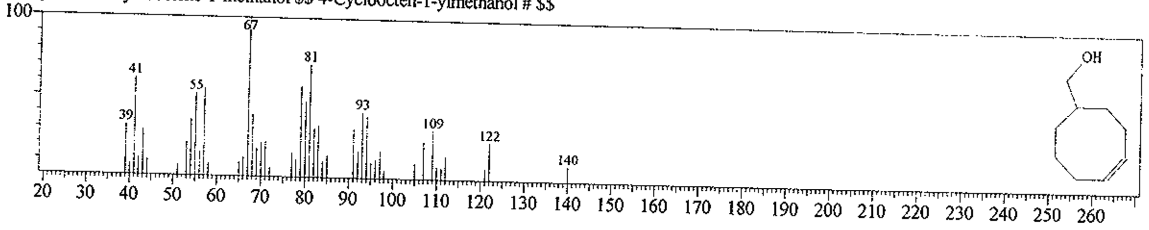

<< Target >>
Line#:6 R.Time:31.633(Scan#:3437) MassPeaks:37
RawMode:Single 31.633(3437) BasePeak:55.10(23485)
BG Mode:31.667(3441) Group 1 - Event 1

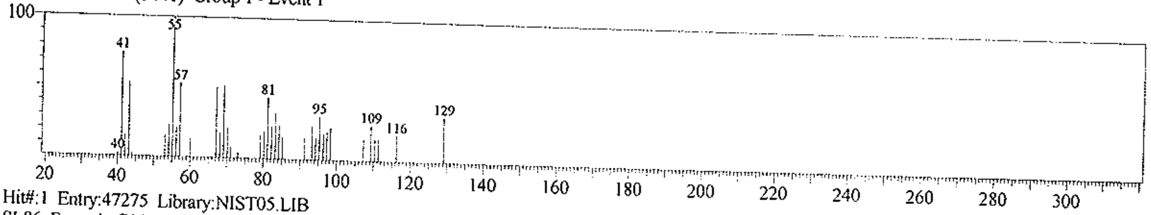

Hit#:1 Entry:47275 Library:NIST05.LIB
SI:86 Formula:C14H26O CAS:65128-96-3 MolWeight:210 RetIndex:1609
CompName:7-Tetradecenal, (Z)- $$ Z-7-Tetradecenal $$ (7Z)-7-Tetradecenal # $$

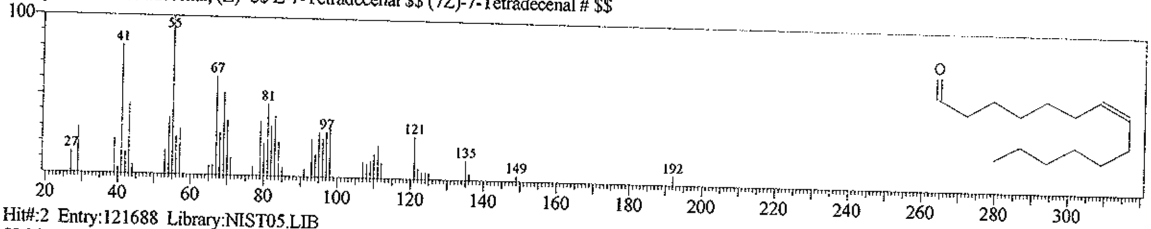

Hit#:2 Entry:121688 Library:NIST05.LIB
SI:86 Formula:C22H42O2 CAS:506-33-2 MolWeight:338 RetIndex:2572
CompName:(E)-13-Docosenoic acid $$ 13-Docosenoic acid, (E)- $$ trans-13-Docosenoic acid $$ Brassidic acid $$ (13E)-13-Docosenoic acid # $$

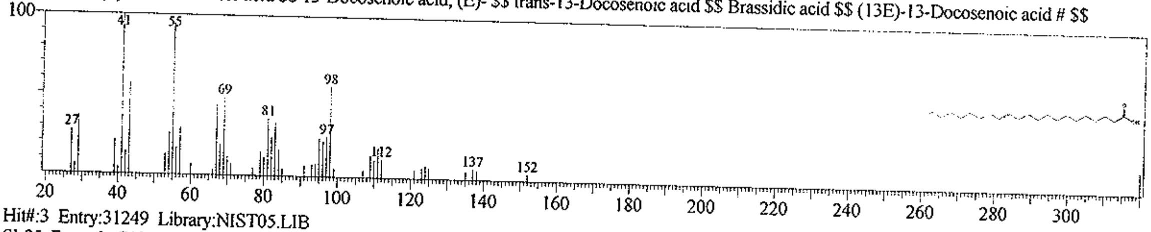

Hit#:3 Entry:31249 Library:NIST05.LIB
SI:85 Formula:C12H22O CAS:286-99-7 MolWeight:182 RetIndex:1450
CompName:13-Oxabicyclo[10.1.0]tridecane $$ Cyclododecane, 1,2-epoxy- $$ Cyclododecene epoxide $$ Epoxycyclododecane $$ Cyclododecane epoxide

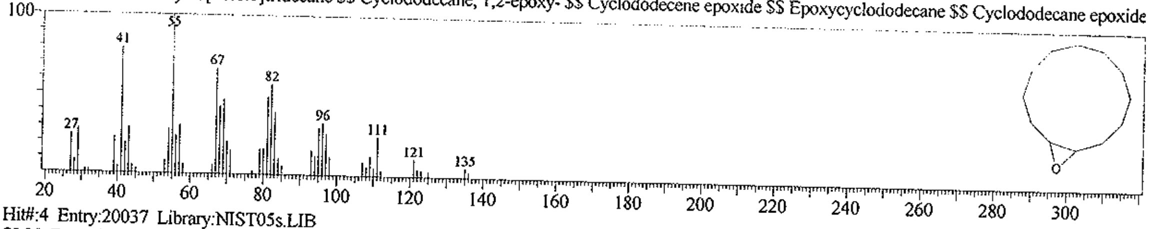

Hit#:4 Entry:20037 Library:NIST05s.LIB
SI:85 Formula:C16H30O CAS:56219-04-6 MolWeight:238 RetIndex:1808
CompName:cis-9-Hexadecenal $$ 9-Hexadecenal, (Z)- $$ (Z)-9-Hexadecenal $$ Z-9-Hexadecenal $$ (9Z)-9-Hexadecenal # $$

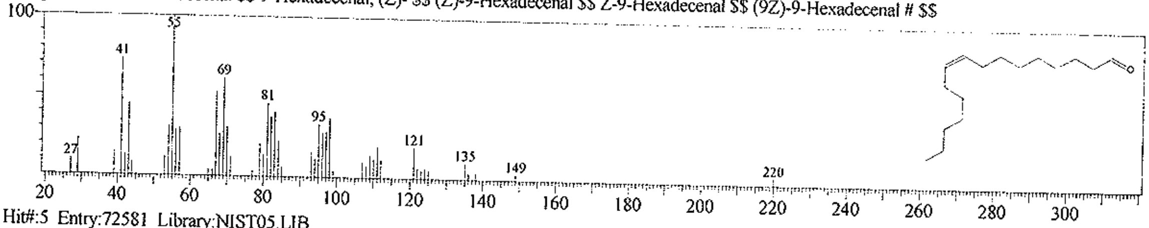

Hit#:5 Entry:72581 Library:NIST05.LIB
SI:85 Formula:C17H32O CAS:60609-53-2 MolWeight:252 RetIndex:1843
CompName:8-Hexadecenal, 14-methyl-, (Z)- $$ 14-Methyl-8-hexadecenal Z; $$ (8Z)-14-Methyl-8-hexadecenal # $$

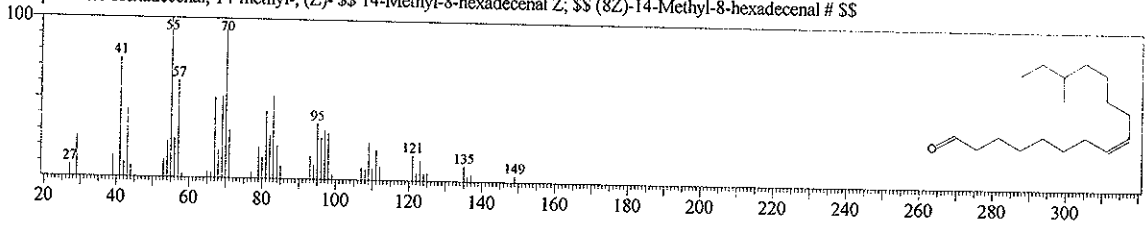

<< Target >>
Line#:7 R.Time:33.875(Scan#:3706) MassPeaks:36
RawMode:Single 33.875(3706) BasePeak:55.10(7476)
BG Mode:33.900(3709) Group 1 - Event 1

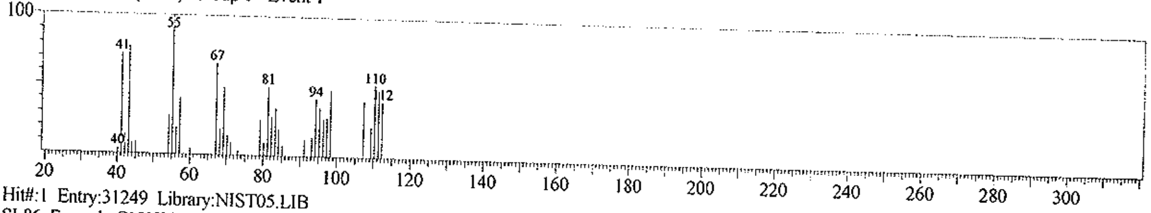

Hit#:1 Entry:31249 Library:NIST05.LIB
SI:86 Formula:C12H22O CAS:286-99-7 MolWeight:182 RetIndex:1450
CompName:13-Oxabicyclo[10.1.0]tridecane $$ Cyclododecane, 1,2-epoxy- $$ Cyclododecene epoxide $$ Epoxycyclododecane $$ Cyclododecane epoxide

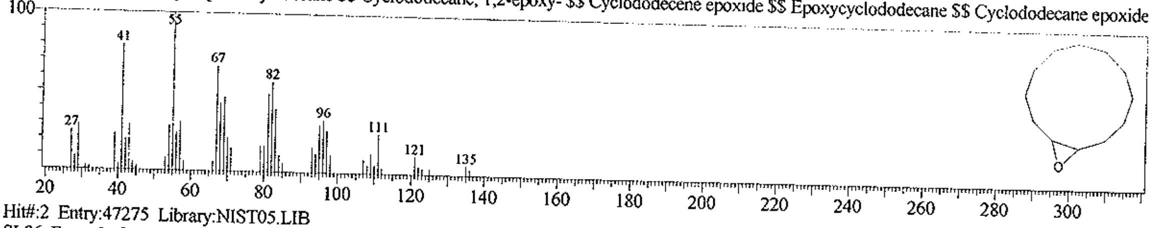

Hit#:2 Entry:47275 Library:NIST05.LIB
SI:86 Formula:C14H26O CAS:65128-96-3 MolWeight:210 RetIndex:1609
CompName:7-Tetradecenal, (Z)- $$ Z-7-Tetradecenal $$ (7Z)-7-Tetradecenal # $$

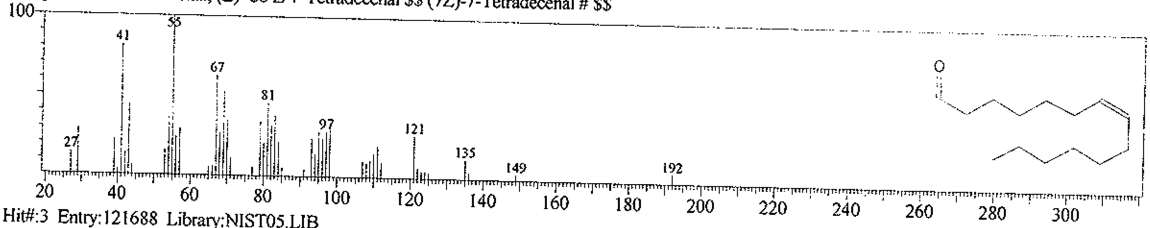

Hit#:3 Entry:121688 Library:NIST05.LIB
SI:86 Formula:C22H42O2 CAS:506-33-2 MolWeight:338 RetIndex:2572
CompName:(E)-13-Docosenoic acid $$ 13-Docosenoic acid, (E)- $$ trans-13-Docosenoic acid $$ Brassidic acid $$ (13E)-13-Docosenoic acid # $$

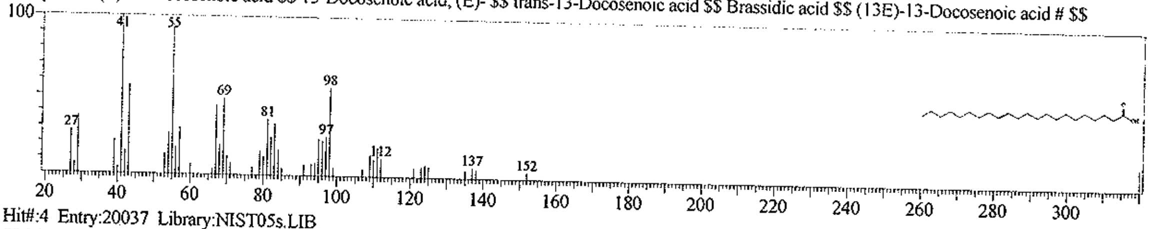

Hit#:4 Entry:20037 Library:NIST05s.LIB
SI:86 Formula:C16H30O CAS:56219-04-6 MolWeight:238 RetIndex:1808
CompName:cis-9-Hexadecenal $$ 9-Hexadecenal, (Z)- $$ (Z)-9-Hexadecenal $$ Z-9-Hexadecenal $$ (9Z)-9-Hexadecenal # $$

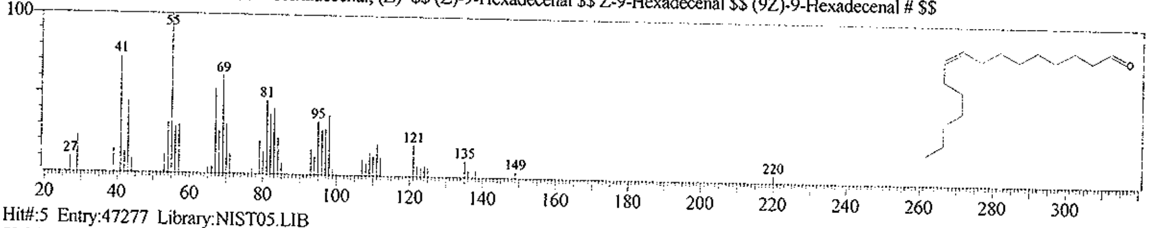

Hit#:5 Entry:47277 Library:NIST05.LIB
SI:86 Formula:C14H26O CAS:85896-31-7 MolWeight:210 RetIndex:1591
CompName:13-Tetradecenal

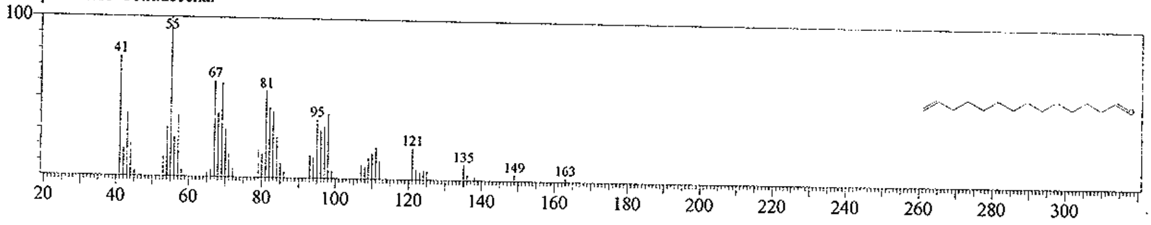

<< Target >>
Line#:8 R.Time:35.583(Scan#:3911) MassPeaks:32
RawMode:Single 35.583(3911) BasePeak:69.15(109865)
BG Mode:35.625(3916) Group 1 - Event 1

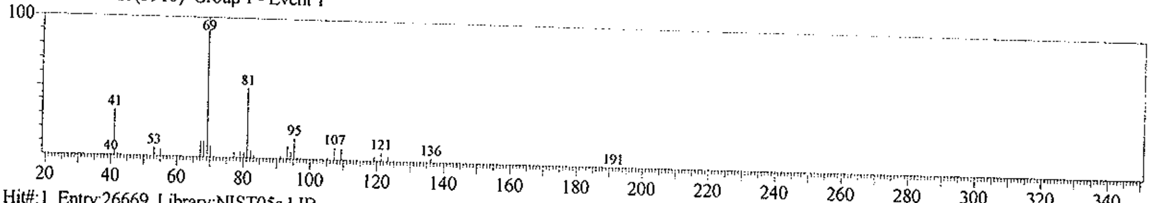

Hit#:1 Entry:26669 Library:NIST05s.LIB
SI:89 Formula:C30H50 CAS:7683-64-9 MolWeight:410 RetIndex:2914
CompName:Squalene $$ 2,6,10,14,18,22-Tetracosahexaene, 2,6,10,15,19,23-hexamethyl- $$ Skvalen $$ Spinacene $$ Supraene $$ (6E,10E,14E,18E)-2,6,

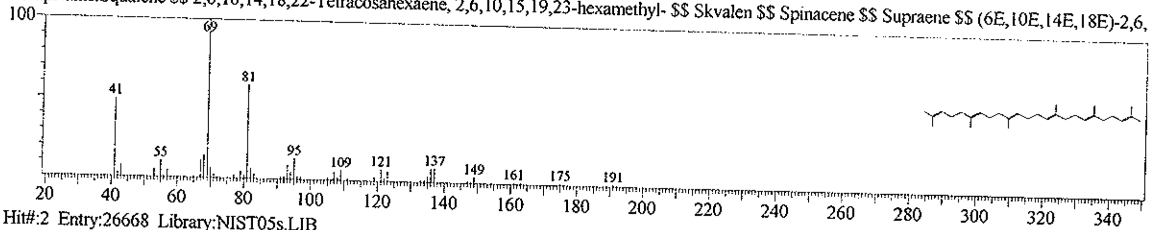

Hit#:2 Entry:26668 Library:NIST05s.LIB
SI:89 Formula:C30H50 CAS:7683-64-9 MolWeight:410 RetIndex:2914
CompName:Squalene $$ 2,6,10,14,18,22-Tetracosahexaene, 2,6,10,15,19,23-hexamethyl- $$ Skvalen $$ Spinacene $$ Supraene $$ (6E,10E,14E,18E)-2,6,

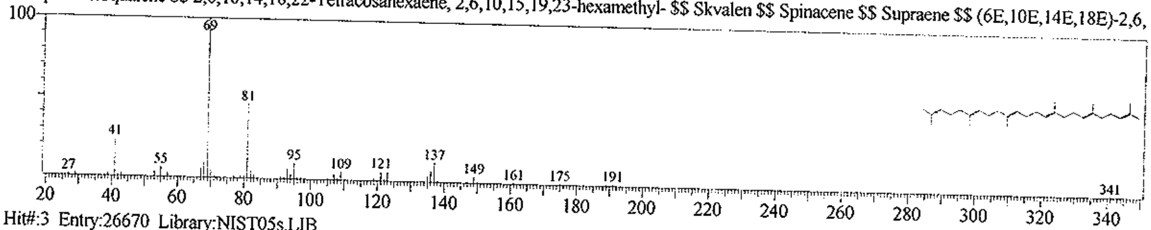

Hit#:3 Entry:26670 Library:NIST05s.LIB
SI:89 Formula:C30H50 CAS:7683-64-9 MolWeight:410 RetIndex:2914
CompName:Squalene $$ 2,6,10,14,18,22-Tetracosahexaene, 2,6,10,15,19,23-hexamethyl- $$ Skvalen $$ Spinacene $$ Supraene $$ (6E,10E,14E,18E)-2,6,

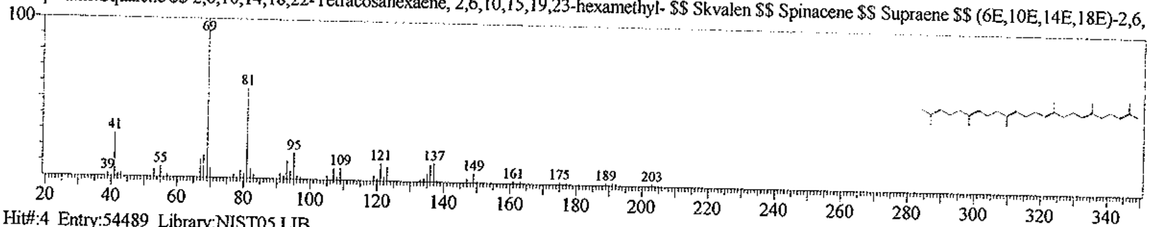

Hit#:4 Entry:54489 Library:NIST05.LIB
SI:89 Formula:C15H26O CAS:0-00-0 MolWeight:222 RetIndex:1710
CompName:Farnesol isomer a

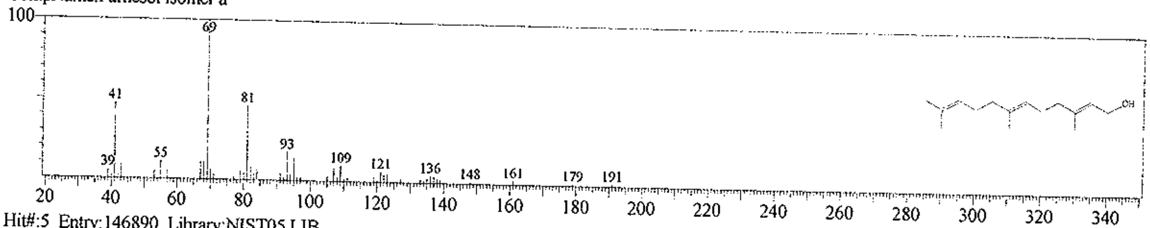

Hit#:5 Entry:146890 Library:NIST05.LIB
SI:88 Formula:C30H50 CAS:111-02-4 MolWeight:410 RetIndex:2914
CompName:2,6,10,14,18,22-Tetracosahexaene, 2,6,10,15,19,23-hexamethyl-, (all-E)- $$ All-trans-Squalene $$ trans-Squalene $$ Spinacen $$ Spinacene $$

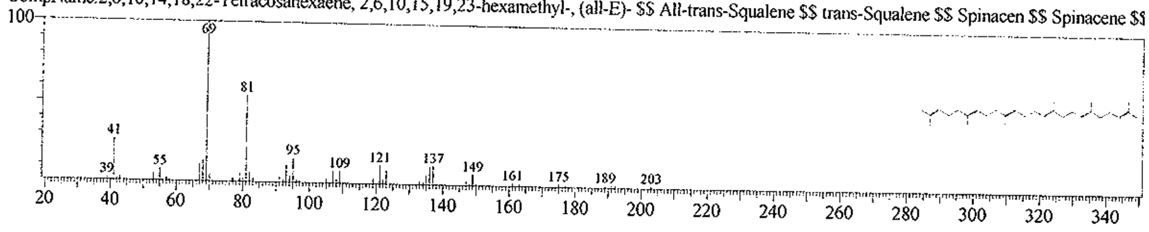

<< Target >>
Line#:9 R.Time:35.783(Scan#:3935) MassPeaks:39
RawMode:Single 35.783(3935) BasePeak:43.10(22486)
BG Mode:35.717(3927) Group 1 - Event 1

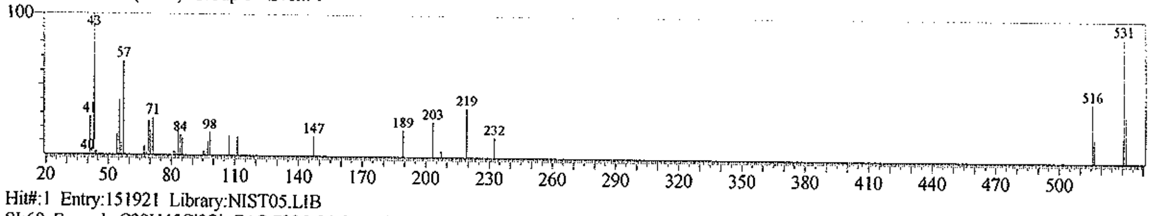

Hit#:1 Entry:151921 Library:NIST05.LIB
SI:68 Formula:C22H45Cl3Si CAS:7325-84-0 MolWeight:442 RetIndex:2647
CompName:Silane, trichlorodocosyl- $$ Docosyltrichlorosilane $$ Trichloro(docosyl)silane # $$

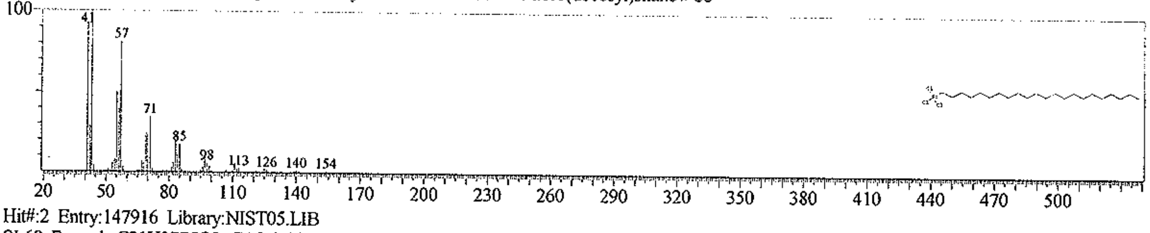

Hit#:2 Entry:147916 Library:NIST05.LIB
SI:68 Formula:C21H37F5O2 CAS:0-00-0 MolWeight:416 RetIndex:1971
CompName:Pentafluoropropionic acid, octadecyl ester

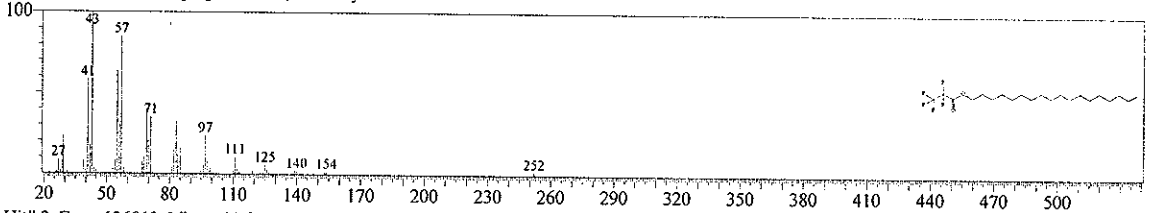

Hit#:3 Entry:136813 Library:NIST05.LIB
SI:68 Formula:C18H31F5O2 CAS:0-00-0 MolWeight:374 RetIndex:1673
CompName:Pentafluoropropionic acid, pentadecyl ester

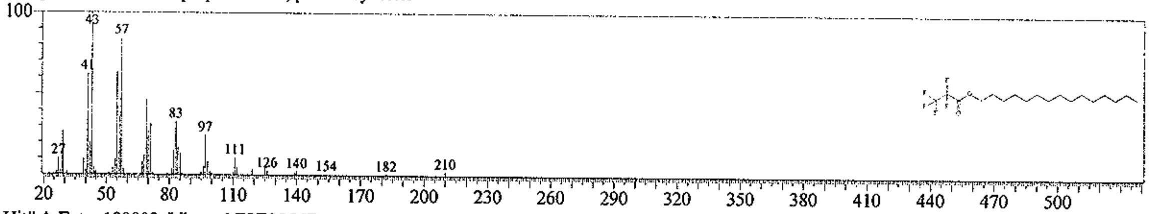

Hit#:4 Entry:109003 Library:NIST05.LIB
SI:67 Formula:C18H34O4 CAS:0-00-0 MolWeight:314 RetIndex:2145
CompName:Oxalic acid, propyl tridecyl ester

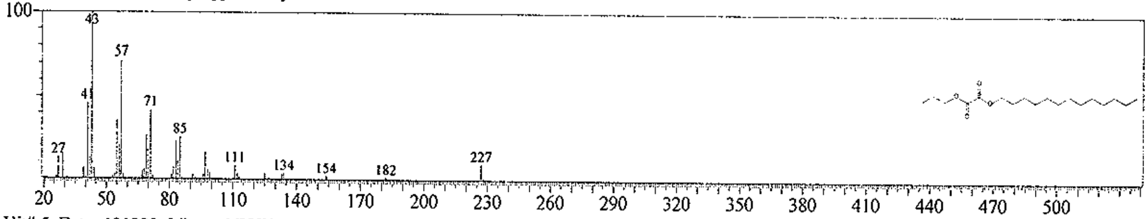

Hit#:5 Entry:131223 Library:NIST05.LIB
SI:67 Formula:C17H29F5O2 CAS:6222-06-6 MolWeight:360 RetIndex:1574
CompName:Pentafluoropropionic acid, tetradecyl ester $$ Tetradecyl 2,2,3,3,3-pentafluoropropanoate # $$

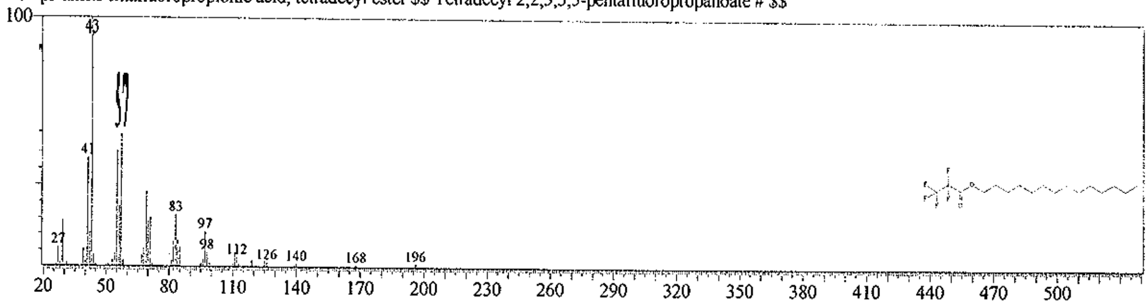

Supplement: Additional file 2 — Gas chromatography-mass spectrometry result of the leaf of Ancistrocladus uncinatus. [file 1746-6148-9-120-S2.pdf]
