# Supplementary material for: Phytochemical analysis and in-vitro anti-African swine fever virus activity of extracts and fractions of Ancistrocladus uncinatus, Hutch and Dalziel (Ancistrocladaceae)
Source: BMC Vet Res. 2013 Jun 19;9:120. doi: 10.1186/1746-6148-9-120 (PMC3694037; doi:10.1186/1746-6148-9-120)

FASINA

GCMS-QP2010 PLUS
SHIMADZU,JAPAN

NARICT, ZARIA
GCMS ANALYSIS

SAMPLE - STEM BARK

Gas Chromatogra[illegible]
Mass spectrometer

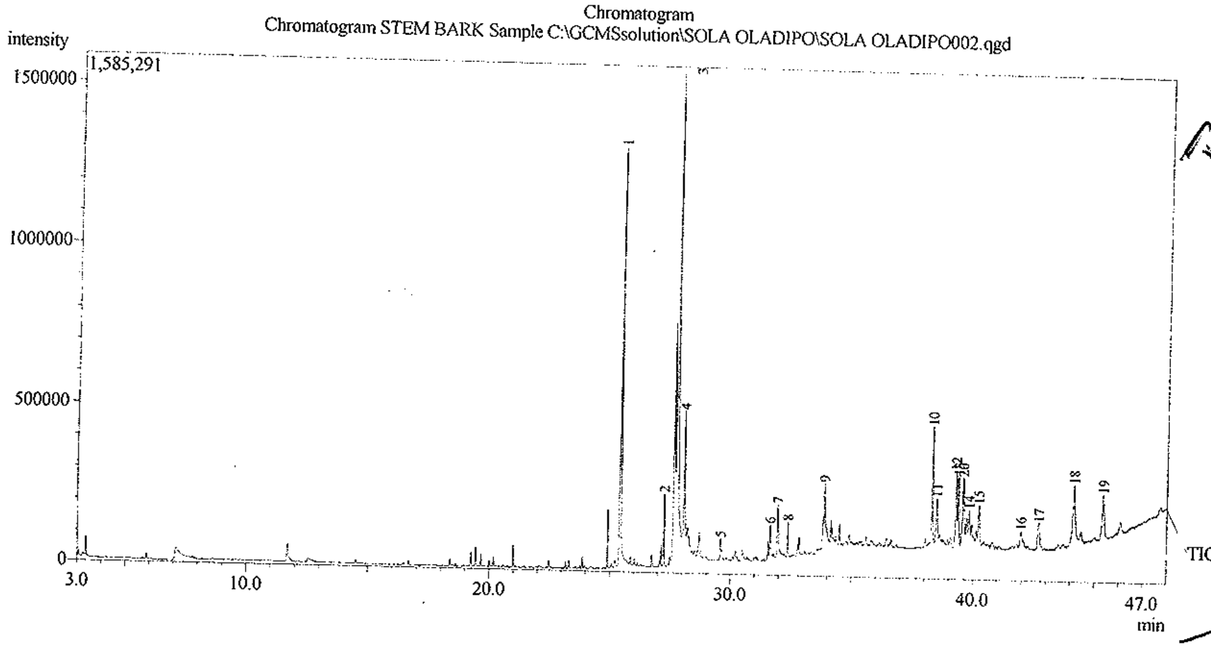

Total Ion Chromatogram

— retention time

Method

[Comment]

═══ Analytical Line 1 ═══

[AOC-20i]

# of Rinses with Presolvent	:5
# of Rinses with Solvent(post)	:5
# of Rinses with Sample	:3
Plunger Speed(Suction)	:High
Viscosity Comp. Time	:0.2 sec
Plunger Speed(Injection)	:High
Syringe Insertion Speed	:High
Injection Mode	:Normal
Pumping Times	:5
Inj. Port Dwell Time	:0.3 sec
Terminal Air Gap	:No
Plunger Washing Speed	:High
Washing Volume	:8uL
Syringe Suction Position	:0.0 mm
Syringe Injection Position	:0.0 mm
Use 3 Solvent Vial	1 vial

[GC-2010]

Column Oven Temp.	:60.0 °C
Injection Temp.	:250.00 °C
Injection Mode	:Split
Flow Control Mode	:Linear Velocity
Pressure	:100.2 kPa
Total Flow	:6.2 mL/min
Column Flow	:1.61 mL/min
Linear Velocity	:46.3 cm/sec
Purge Flow	:3.0 mL/min
Split Ratio	1.0
High Pressure Injection	:OFF
Carrier Gas Saver	:OFF
Splitter Hold	:OFF

Oven Temp. Program

Rate	Temperature(°C)	Hold Time(min)
-	60.0	3.00
7.00	140.0	0.00
7.00	280.0	15.00

< Ready Check Heat Unit >
Column Oven : Yes
SPL2 : Yes
MS : Yes
< Ready Check Detector(FTD) >
< Ready Check Baseline Drift >
< Ready Check Injection Flow >
SPL2 Carrier : Yes
SPL2 Purge : Yes
< Ready Check APC Flow >
< Ready Check Detector APC Flow >
External Wait :No
Equilibrium Time :3.0 min

[GC Program]

[GCMS-QP2010 Plus]
IonSourceTemp :200.00 °C
Interface Temp. :250.00 °C
Solvent Cut Time :2.50 min
Detector Gain Mode :Relative
Detector Gain :0.00 kV
Threshold :3000

[MS Table]
--Group 1 - Event 1--
Start Time :3.00min
End Time :48.00min
ACQ Mode :Scan
Event Time :0.50sec
Scan Speed :1666
Start m/z :40.00
End m/z :800.00

Sample Inlet Unit :GC

[MS Program]
Use MS Program :OFF

Compare to pg 5

Spectrum

Line#:1 R.Time:25.4(Scan#:2692)
MassPeaks:53
RawMode:Single 25.4(2692) BasePeak:43(122999)
BG Mode:25.5(2695) Group 1 - Event 1

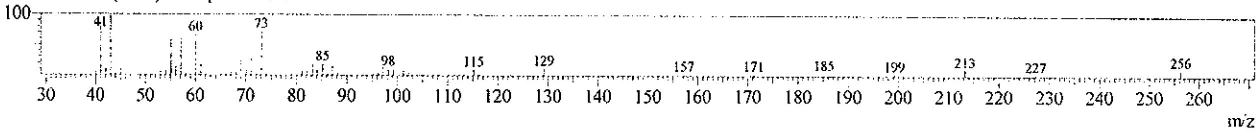

Line#:2 R.Time:27.2(Scan#:2910)
MassPeaks:27
RawMode:Single 27.2(2910) BasePeak:55(16837)
BG Mode:27.3(2913) Group 1 - Event 1

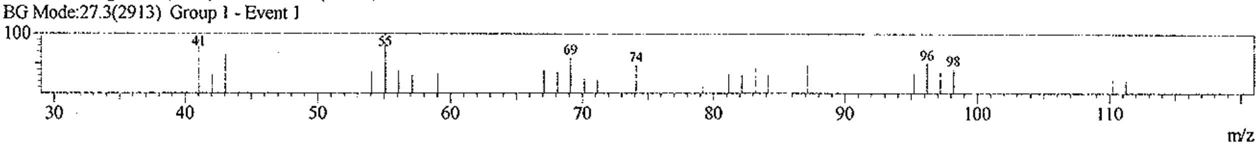

Line#:3 R.Time:27.8(Scan#:2972)
MassPeaks:57
RawMode:Single 27.8(2972) BasePeak:55(114294)
BG Mode:27.8(2976) Group 1 - Event 1

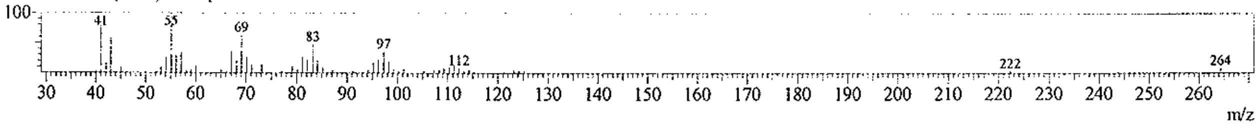

Line#:4 R.Time:28.1(Scan#:3008)
MassPeaks:42
RawMode:Single 28.1(3008) BasePeak:43(21385)
BG Mode:28.1(3011) Group 1 - Event 1

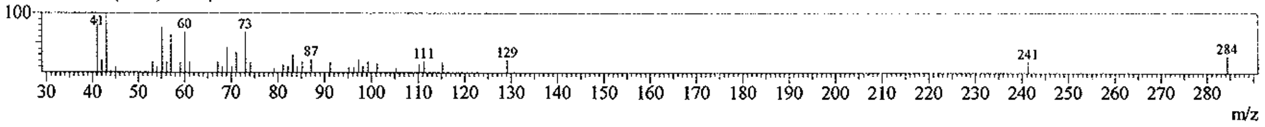

Line#:5 R.Time:29.6(Scan#:3191)
MassPeaks:17
RawMode:Single 29.6(3191) BasePeak:98(6282)
BG Mode:29.6(3194) Group 1 - Event 1

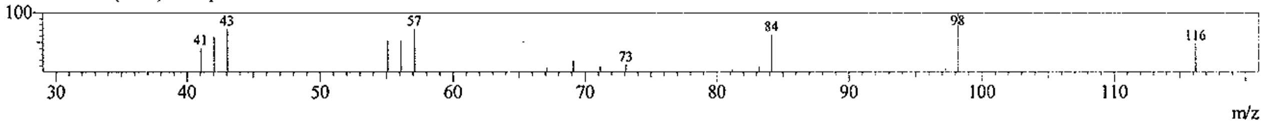

Line#:6 R.Time:31.6(Scan#:3437)
MassPeaks:21
RawMode:Single 31.6(3437) BasePeak:55(6908)
BG Mode:31.7(3440) Group 1 - Event 1

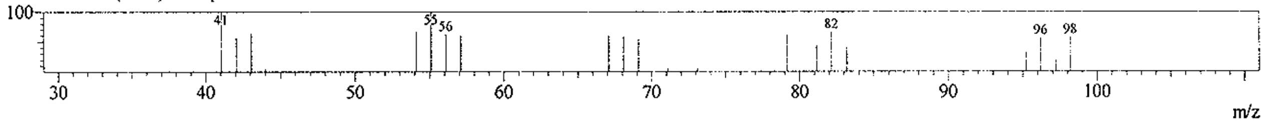

Line#:7 R.Time:31.9(Scan#:3474)
MassPeaks:27
RawMode:Single 31.9(3474) BasePeak:43(13734)
BG Mode:32.0(3481) Group 1 - Event 1

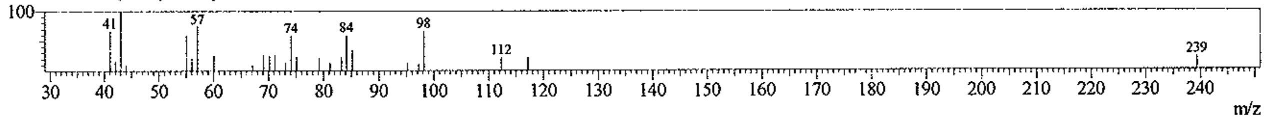

Line#:8 R.Time:32.4(Scan#:3525)
MassPeaks:17
RawMode:Single 32.4(3525) BasePeak:57(12405)
BG Mode:32.4(3528) Group 1 - Event 1

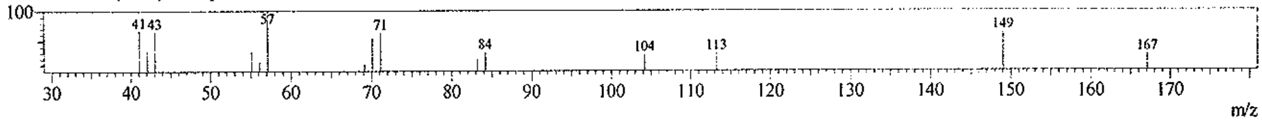

Line#:9 R.Time:33.9(Scan#:3706)
MassPeaks:33
RawMode:Single 33.9(3706) BasePeak:55(8970)
BG Mode:33.9(3709) Group 1 - Event 1

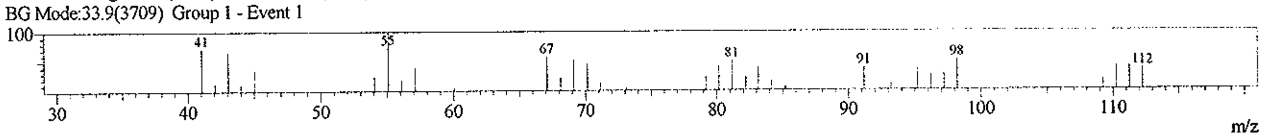

Line#:10 R.Time:38.3(Scan#:4238)
MassPeaks:34
RawMode:Single 38.3(4238) BasePeak:406(134255)
BG Mode:38.3(4242) Group 1 - Event 1

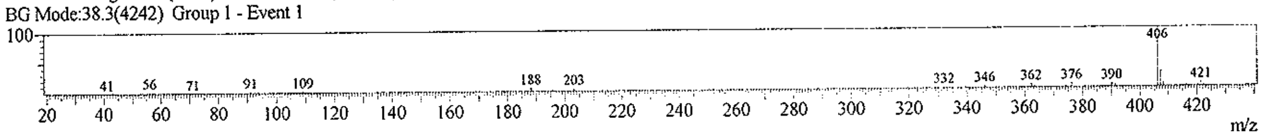

Line#:11 R.Time:38.5(Scan#:4261)
MassPeaks:16
RawMode:Single 38.5(4261) BasePeak:420(52495)
BG Mode:38.5(4265) Group 1 - Event 1

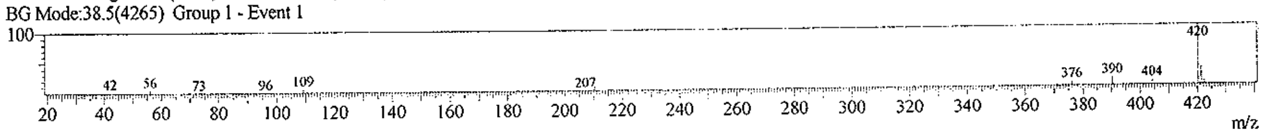

Line#:12 R.Time:39.3(Scan#:4355)
MassPeaks:31
RawMode:Single 39.3(4355) BasePeak:392(67066)
BG Mode:39.3(4360) Group 1 - Event 1

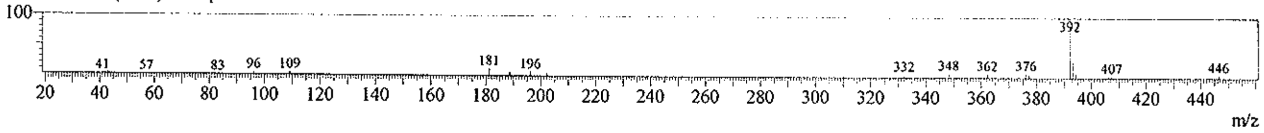

Line#:13 R.Time:39.4(Scan#:4367)
MassPeaks:19
RawMode:Single 39.4(4367) BasePeak:392(56381)
BG Mode:39.4(4363) Group 1 - Event 1

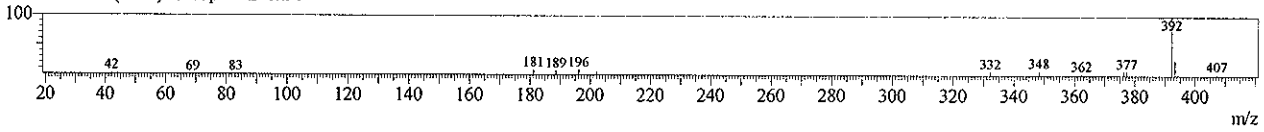

Line#:14 R.Time:39.8(Scan#:4420)
MassPeaks:15
RawMode:Single 39.8(4420) BasePeak:392(24520)
BG Mode:39.8(4416) Group 1 - Event 1

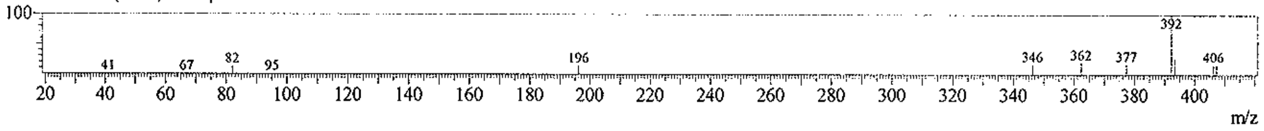

Line#:15 R.Time:40.2(Scan#:4468)
MassPeaks:16
RawMode:Single 40.2(4468) BasePeak:392(33293)
BG Mode:40.3(4472) Group 1 - Event 1

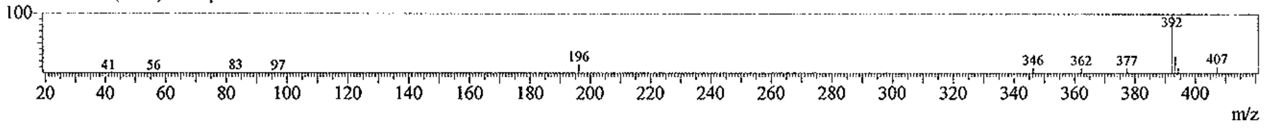

Line#:16 R.Time:42.0(Scan#:4677)
MassPeaks:20
RawMode:Single 42.0(4677) BasePeak:91(3279)
BG Mode:42.0(4679) Group 1 - Event 1

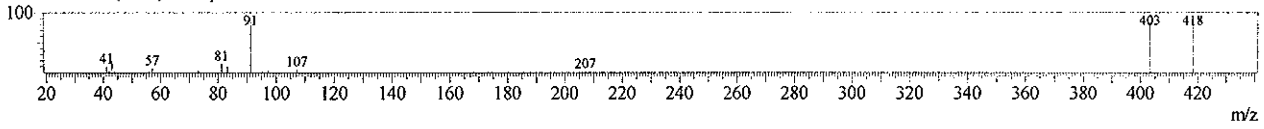

Line#:17 R.Time:42.7(Scan#:4764)
MassPeaks:24
RawMode:Single 42.7(4764) BasePeak:55(5539)
BG Mode:42.7(4770) Group 1 - Event 1

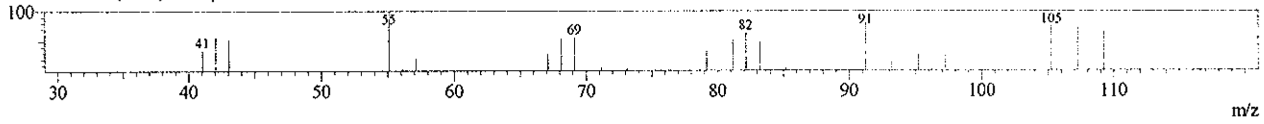

Line#:18 R.Time:44.1(Scan#:4938)
MassPeaks:32
RawMode:Single 44.1(4938) BasePeak:43(18086)
BG Mode:44.2(4948) Group 1 - Event 1

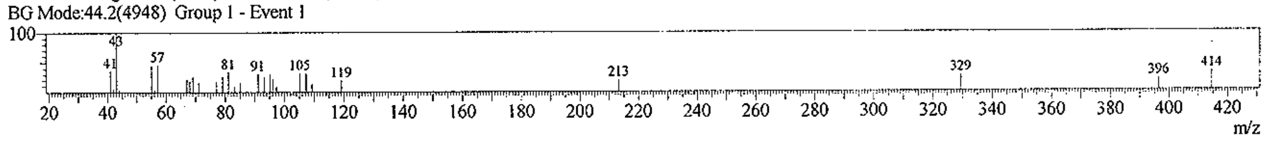

Line#:19 R.Time:45.4(Scan#:5083)
MassPeaks:34
RawMode:Single 45.4(5083) BasePeak:95(5560)
BG Mode:45.4(5091) Group 1 - Event 1

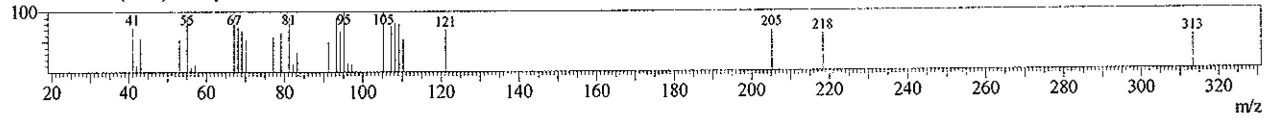

Line#:20 R.Time:39.6(Scan#:4390)
MassPeaks:20
RawMode:Single 39.6(4390) BasePeak:406(16421)
BG Mode:39.6(4392) Group 1 - Event 1

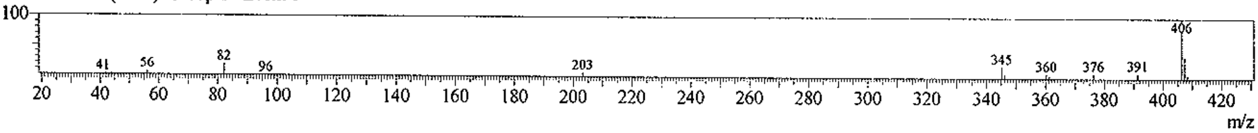

Spectrum Comparison

Library

<< Target >>
Line#:1 R.Time:25.425(Scan#:2692) MassPeaks:53
RawMode:Single 25.425(2692) BasePeak:43.05(122999)
BG Mode:25.450(2695) Group 1 - Event 1

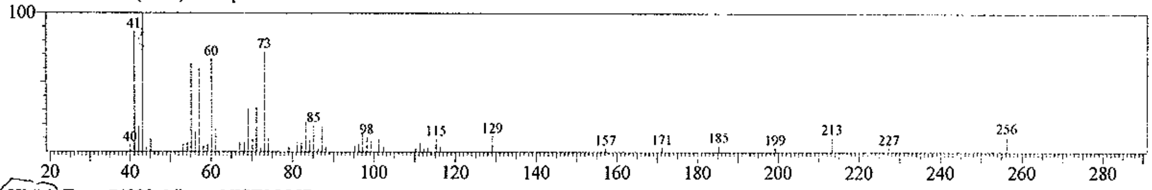

Hit#:1 Entry:74999 Library:NIST05.LIB
SI:94 Formula:C16H32O2 CAS:57-10-3 MolWeight:256 RetIndex:1968
CompName:n-Hexadecanoic acid $$ Hexadecanoic acid $$ n-Hexadecoic acid $$ Palmitic acid $$ Pentadecanecarboxylic acid $$ 1-Pentadecanecarboxylic

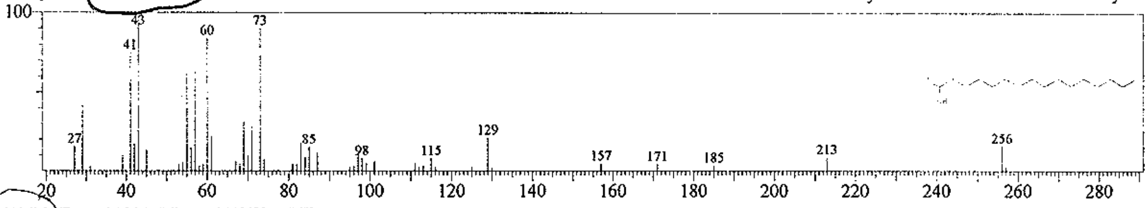

Hit#:2 Entry:21331 Library:NIST05s.LIB
SI:92 Formula:C16H32O2 CAS:57-10-3 MolWeight:256 RetIndex:1968
CompName:n-Hexadecanoic acid $$ Hexadecanoic acid $$ n-Hexadecoic acid $$ Palmitic acid $$ Pentadecanecarboxylic acid $$ 1-Pentadecanecarboxylic

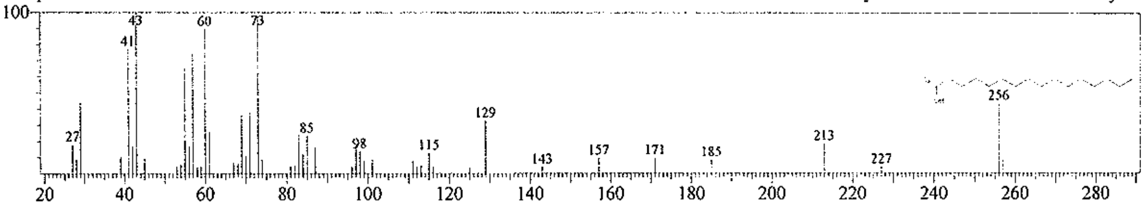

Hit#:3 Entry:22977 Library:NIST05s.LIB
SI:92 Formula:C18H36O2 CAS:57-11-4 MolWeight:284 RetIndex:2167
CompName:Octadecanoic acid $$ Stearic acid $$ n-Octadecanoic acid $$ Humko Industrene R $$ Hydrofol Acid 150 $$ Hystrene S-97 $$ Hystrene T-70 $

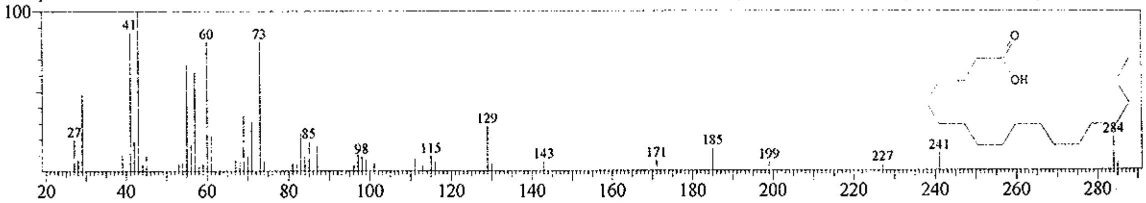

Hit#:4 Entry:66523 Library:NIST05.LIB
SI:91 Formula:C15H30O2 CAS:1002-84-2 MolWeight:242 RetIndex:1869
CompName:Pentadecanoic acid $$ Pentadecylic acid $$ n-Pentadecanoic acid $$ n-Pentadecylic acid $$

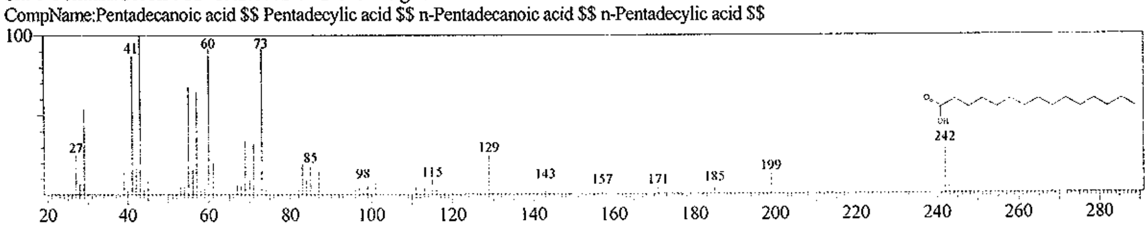

<< Target >>
Line#:1 R.Time:25.425(Scan#:2692) MassPeaks:53
RawMode:Single 25.425(2692) BasePeak:43.05(122999)
BG Mode:25.450(2695) Group 1 - Event 1

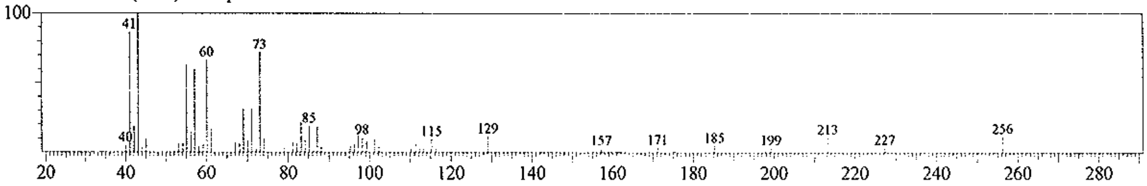

Hit#:5 Entry:136237 Library:NIST05.LIB
SI:91 Formula:C22H44O4 CAS:106-11-6 MolWeight:372 RetIndex:2694
CompName:Octadecanoic acid, 2-(2-hydroxyethoxy)ethyl ester $$ Aqua Cera $$ Atlas G 2146 $$ Cerasynt $$ Cerasynt Special $$ Clindrol SDG $$ Diethy

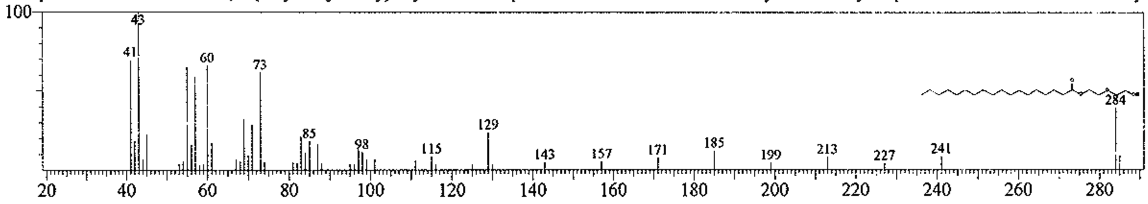

<< Target >>
Line#:2 R.Time:27.242(Scan#:2910) MassPeaks:27
RawMode:Single 27.242(2910) BasePeak:55.10(16837)
BG Mode:27.267(2913) Group 1 - Event 1

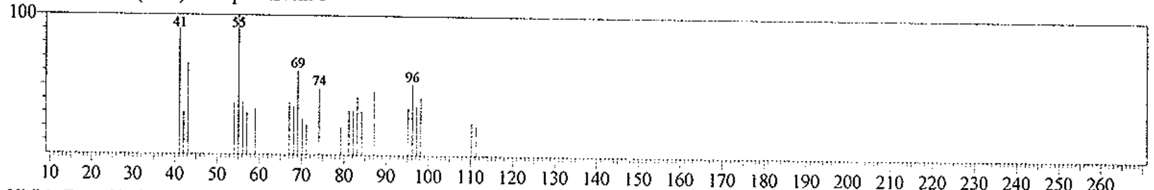

Hit#:1 Entry:82163 Library:NIST05.LIB
SI:88 Formula:C17H32O2 CAS:56875-67-3 MolWeight:268 RetIndex:1886
CompName:7-Hexadecenoic acid, methyl ester, (Z)- $$ Methyl (7E)-7-hexadecenoate # $$

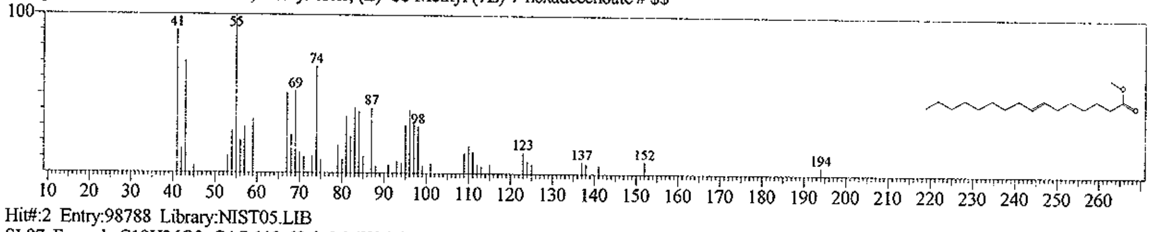

Hit#:2 Entry:98788 Library:NIST05.LIB
SI:87 Formula:C19H36O2 CAS:112-62-9 MolWeight:296 RetIndex:2085
CompName:9-Octadecenoic acid (Z)-, methyl ester $$ Oleic acid, methyl ester $$ Emery oleic acid ester 2301 $$ Methyl cis-9-octadecenoate $$ Methyl ole:

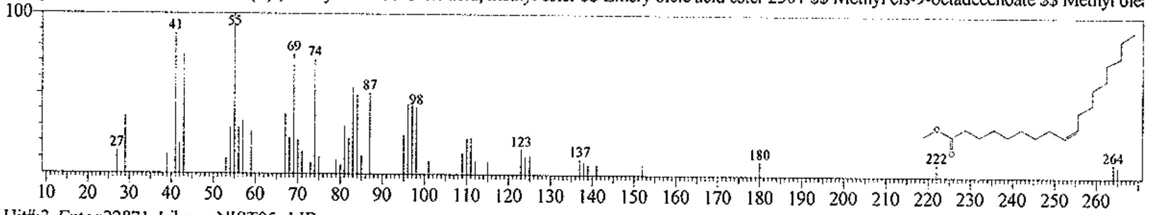

Hit#:3 Entry:22871 Library:NIST05s.LIB
SI:87 Formula:C18H34O2 CAS:10152-61-1 MolWeight:282 RetIndex:1941
CompName:Cyclopropaneoctanoic acid, 2-hexyl-, methyl ester $$ Methyl 8-(2-hexylcyclopropyl)octanoate # $$

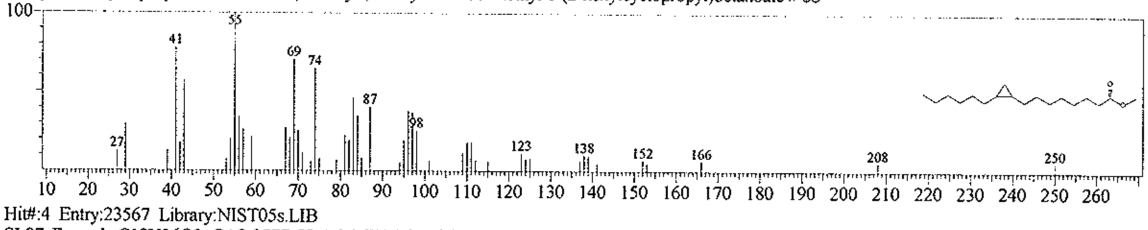

Hit#:4 Entry:23567 Library:NIST05s.LIB
SI:87 Formula:C19H36O2 CAS:2777-58-4 MolWeight:296 RetIndex:2085
CompName:6-Octadecenoic acid, methyl ester, (Z)- $$ Methyl cis-6-octadecenoate $$ Methyl petroselinate $$ Methyl (6Z)-6-octadecenoate # $$

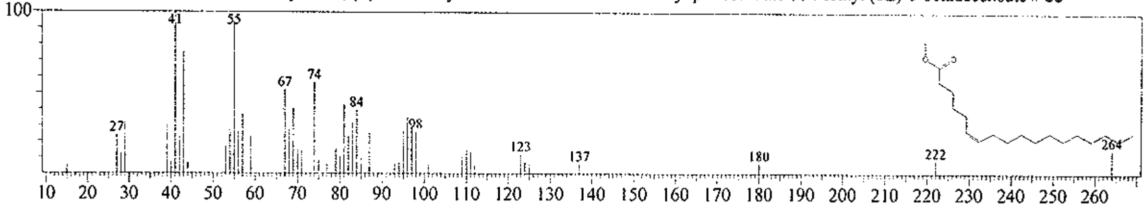

Hit#:5 Entry:23570 Library:NIST05s.LIB
SI:87 Formula:C19H36O2 CAS:1937-62-8 MolWeight:296 RetIndex:2085
CompName:9-Octadecenoic acid, methyl ester, (E)- $$ Elaidic acid, methyl ester $$ Methyl elaidate $$ Methyl trans-9-octadecenoate $$ (E)-9-Octadecenoi

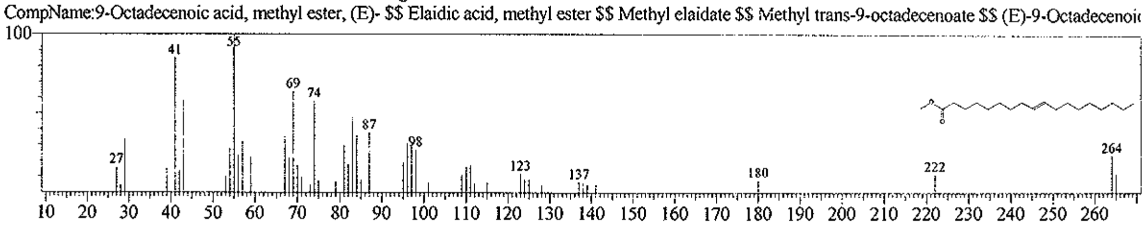

<< Target >>
Line#:3 R.Time:27.758(Scan#:2972) MassPeaks:57
RawMode:Single 27.758(2972) BasePeak:55.10(114294)
BG Mode:27.792(2976) Group 1 - Event 1

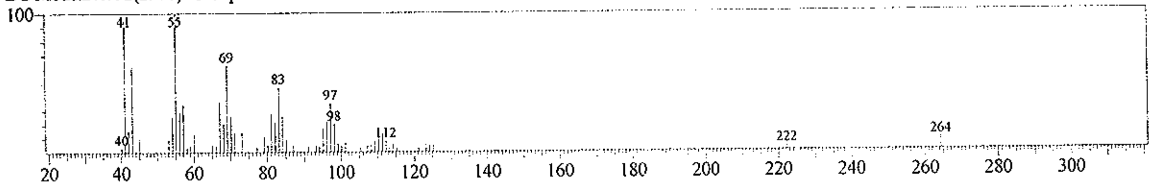

Hit#:1 Entry:73685 Library:NIST05.LIB
SI:92 Formula:C16H30O2 CAS:2091-29-4 MolWeight:254 RetIndex:1976
CompName:9-Hexadecenoic acid $$ (9E)-9-Hexadecenoic acid # $$

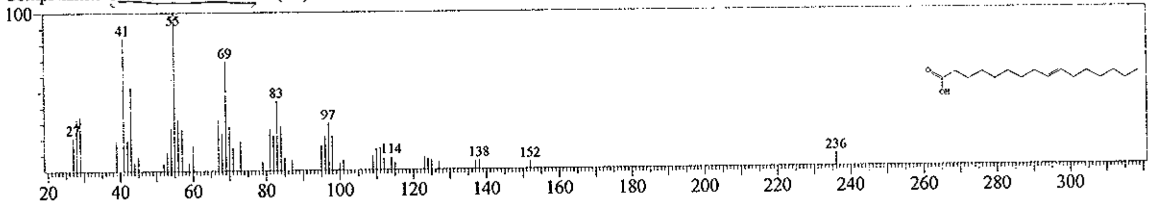

Hit#:2 Entry:121691 Library:NIST05.LIB
SI:91 Formula:C22H42O2 CAS:112-86-7 MolWeight:338 RetIndex:2572
CompName:Erucic acid $$ 13-Docosenoic acid, (Z)- $$ .delta.13-cis-Docosenoic acid $$ cis-13-Docosenoic acid $$ (Z)-13-Docosenoic acid $$ Prifrac 299

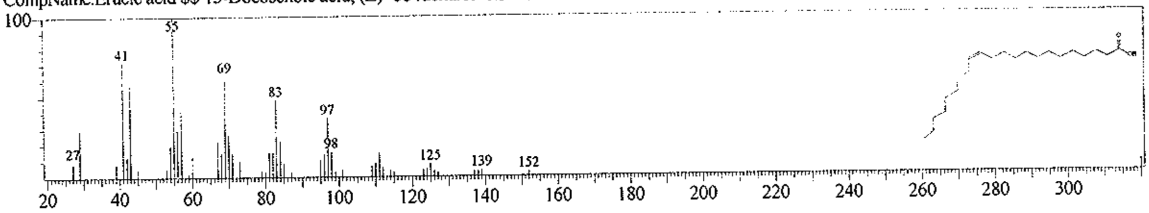

Hit#:3 Entry:22869 Library:NIST05s.LIB
SI:90 Formula:C18H34O2 CAS:112-80-1 MolWeight:282 RetIndex:2175
CompName:Oleic Acid $$ 9-Octadecenoic acid (Z)- $$ .delta.(Sup9)-cis-Oleic acid $$ cis-.delta.(Sup9)-Octadecenoic acid $$ cis-Oleic Acid $$ cis-9-Octad

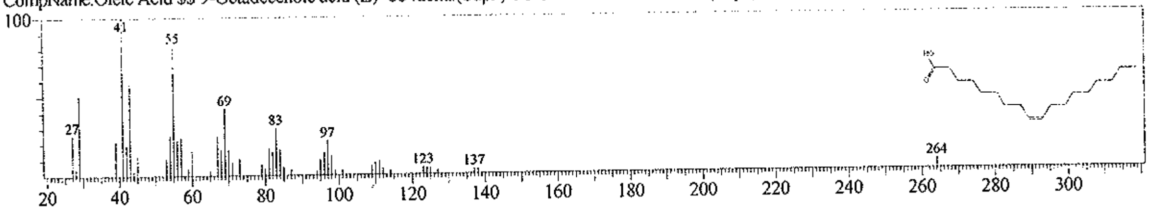

Hit#:4 Entry:56983 Library:NIST05.LIB
SI:90 Formula:C14H26O2 CAS:0-00-0 MolWeight:226 RetIndex:1777
CompName:Z-11-Tetradecenoic acid

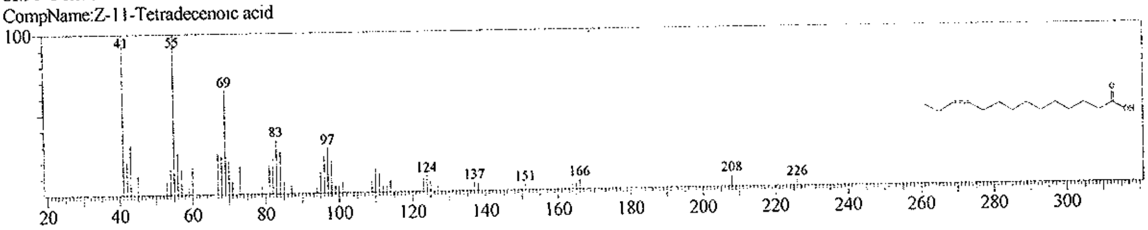

Hit#:5 Entry:90577 Library:NIST05.LIB
SI:90 Formula:C18H34O2 CAS:112-80-1 MolWeight:282 RetIndex:2175
CompName:Oleic Acid $$ 9-Octadecenoic acid (Z)- $$ .delta.(Sup9)-cis-Oleic acid $$ cis-.delta.(Sup9)-Octadecenoic acid $$ cis-Oleic Acid $$ cis-9-Octad

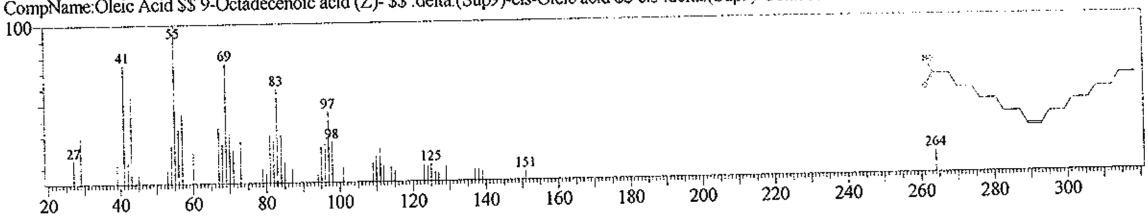

<< Target >>
Line#:4 R.Time:28.058(Scan#:3008) MassPeaks:42
RawMode:Single 28.058(3008) BasePeak:43.05(21385)
BG Mode:28.083(3011) Group 1 - Event 1

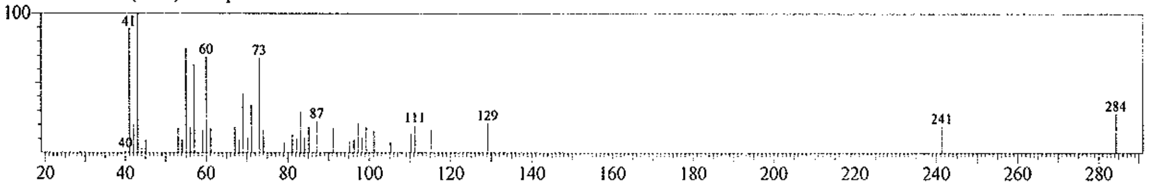

Hit#:1 Entry:22977 Library:NIST05s.LIB
SI:89 Formula:C18H36O2 CAS:57-11-4 MolWeight:284 RetIndex:2167
CompName:Octadecanoic acid $$ Stearic acid $$ n-Octadecanoic acid $$ Humko Industrene R $$ Hydrofol Acid 150 $$ Hystrene S-97 $$ Hystrene T-70 $

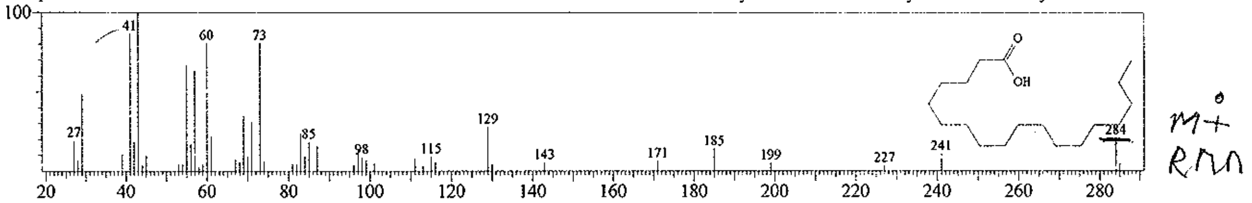

Hit#:2 Entry:22978 Library:NIST05s.LIB
SI:88 Formula:C18H36O2 CAS:57-11-4 MolWeight:284 RetIndex:2167
CompName:Octadecanoic acid $$ Stearic acid $$ n-Octadecanoic acid $$ Humko Industrene R $$ Hydrofol Acid 150 $$ Hystrene S-97 $$ Hystrene T-70 $

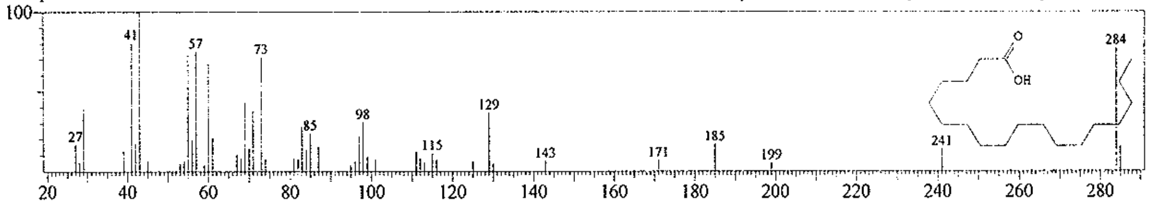

Hit#:3 Entry:22979 Library:NIST05s.LIB
SI:88 Formula:C18H36O2 CAS:57-11-4 MolWeight:284 RetIndex:2167
CompName:Octadecanoic acid $$ Stearic acid $$ n-Octadecanoic acid $$ Humko Industrene R $$ Hydrofol Acid 150 $$ Hystrene S-97 $$ Hystrene T-70 $

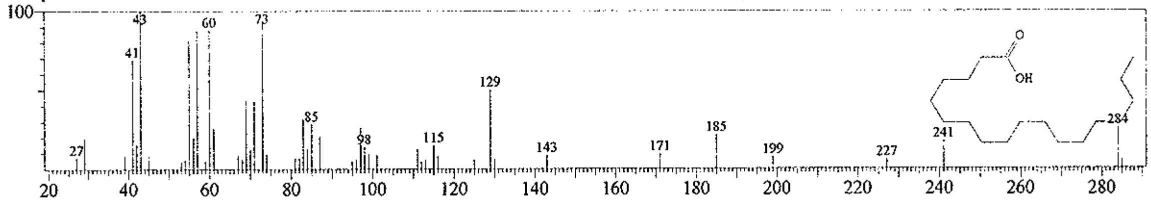

Hit#:4 Entry:136237 Library:NIST05.LIB
SI:87 Formula:C22H44O4 CAS:106-11-6 MolWeight:372 RetIndex:2694
CompName:Octadecanoic acid, 2-(2-hydroxyethoxy)ethyl ester $$ Aqua Cera $$ Atlas G 2146 $$ Cerasynt $$ Cerasynt Special $$ Clindrol SDG $$ Diethy

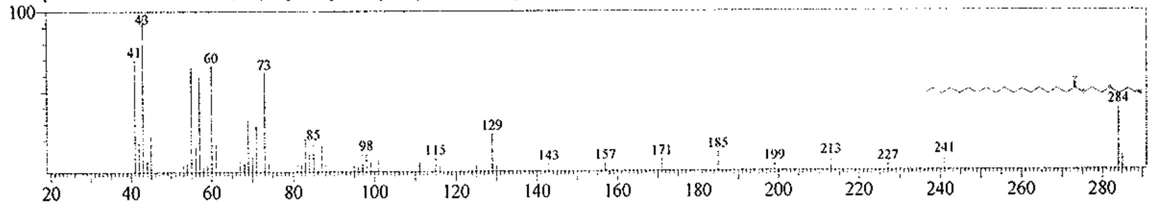

Hit#:5 Entry:91895 Library:NIST05.LIB
SI:87 Formula:C18H36O2 CAS:57-11-4 MolWeight:284 RetIndex:2167
CompName:Octadecanoic acid $$ Stearic acid $$ n-Octadecanoic acid $$ Humko Industrene R $$ Hydrofol Acid 150 $$ Hystrene S-97 $$ Hystrene T-70 $

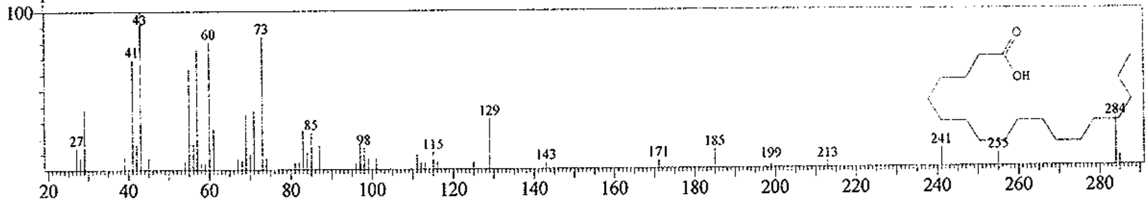

<< Target >>
Line#:5 R.Time:29.583(Scan#:3191) MassPeaks:17
RawMode:Single 29.583(3191) BasePeak:98.20(6282)
BG Mode:29.608(3194) Group 1 - Event 1

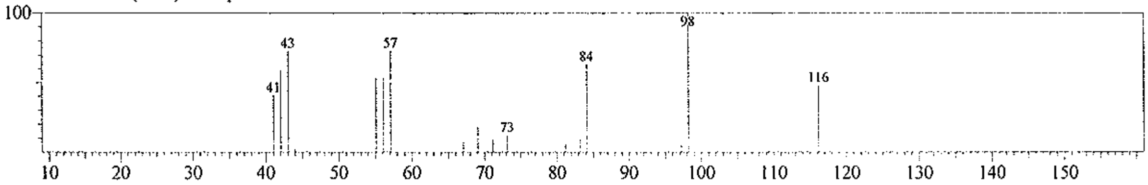

Hit#:1 Entry:17418 Library:NIST05.LIB
SI:79 Formula:C10H21N CAS:35448-31-8 MolWeight:155 RetIndex:1103
CompName:1-Butanamine, 3-methyl-N-(3-methylbutylidene)- $$ 3-Methyl-N-[(E)-3-methylbutylidene]-1-butanamine # $$

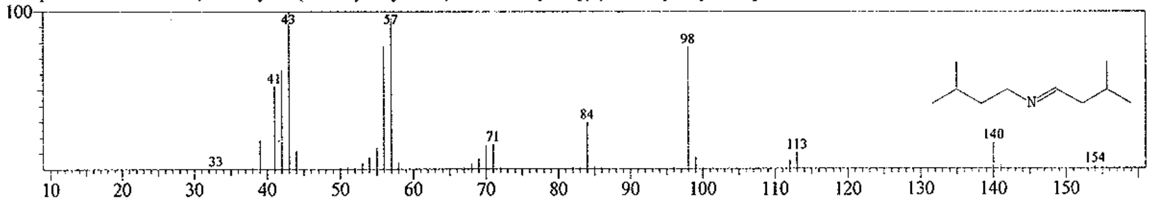

Hit#:2 Entry:1569 Library:NIST05.LIB
SI:77 Formula:C5H10N2 CAS:66075-09-0 MolWeight:98 RetIndex:755
CompName:Formaldehyde, methyl(2-propenyl)hydrazone $$ Formaldehyde allyl(methyl)hydrazone # $$

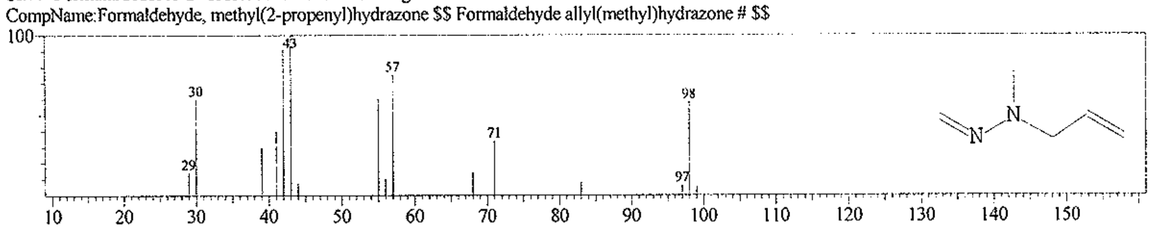

Hit#:3 Entry:1545 Library:NIST05s.LIB
SI:76 Formula:C6H10O CAS:6672-30-6 MolWeight:98 RetIndex:832
CompName:(R)-(+)-3-Methylcyclopentanone $$ Cyclopentanone, 3-methyl-, (R)- $$ 3-Methylcyclopentanone # $$

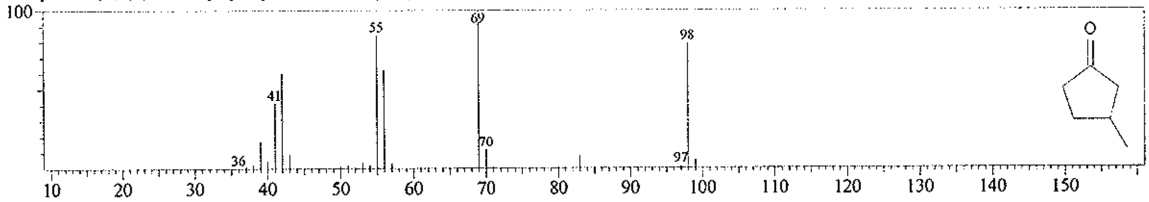

Hit#:4 Entry:73696 Library:NIST05.LIB
SI:76 Formula:C16H30O2 CAS:0-00-0 MolWeight:254 RetIndex:1787
CompName:11-Tridecenyl propionate

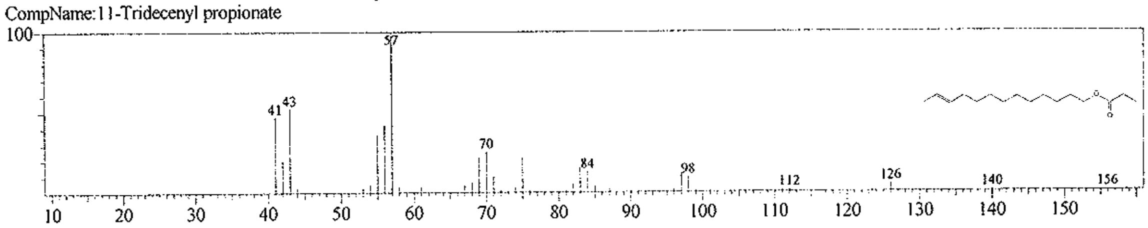

Hit#:5 Entry:12753 Library:NIST05.LIB
SI:75 Formula:C9H20O CAS:18450-74-3 MolWeight:144 RetIndex:1030
CompName:1-Heptanol, 2,4-dimethyl-, (2S,4R)-(-)- $$ 2,4-Dimethyl-1-heptanol # $$

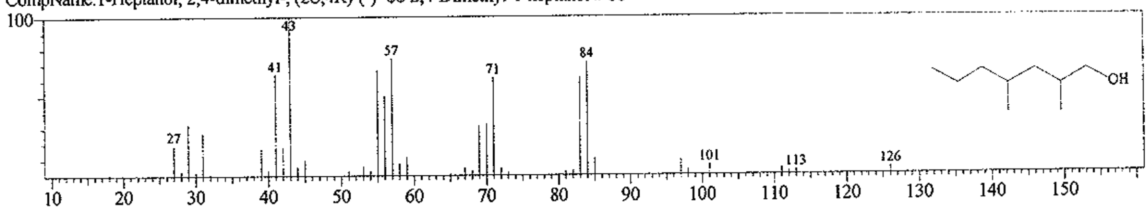

<< Target >>
Line#:5 R.Time:29.583(Scan#:3191) MassPeaks:17
RawMode:Single 29.583(3191) BasePeak:98.20(6282)
BG Mode:29.608(3194) Group 1 - Event 1

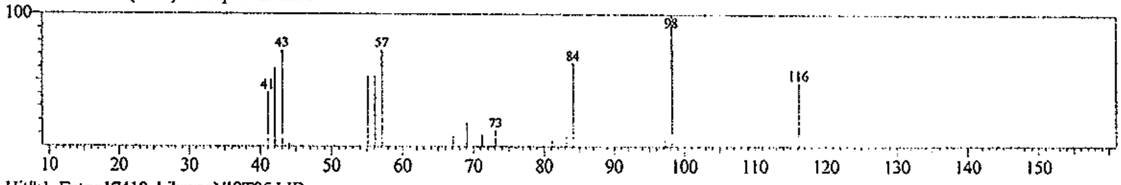

Hit#:1 Entry:17418 Library:NIST05.LIB
SI:79 Formula:C10H21N CAS:35448-31-8 MolWeight:155 RetIndex:1103
CompName:1-Butanamine, 3-methyl-N-(3-methylbutylidene)- $$ 3-Methyl-N-[(E)-3-methylbutylidene]-1-butanamine # $$

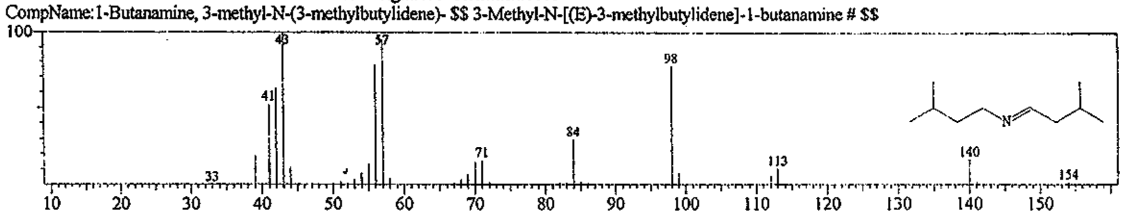

Hit#:2 Entry:1569 Library:NIST05.LIB
SI:77 Formula:C5H10N2 CAS:66075-09-0 MolWeight:98 RetIndex:755
CompName:Formaldehyde, methyl(2-propenyl)hydrazone $$ Formaldehyde allyl(methyl)hydrazone # $$

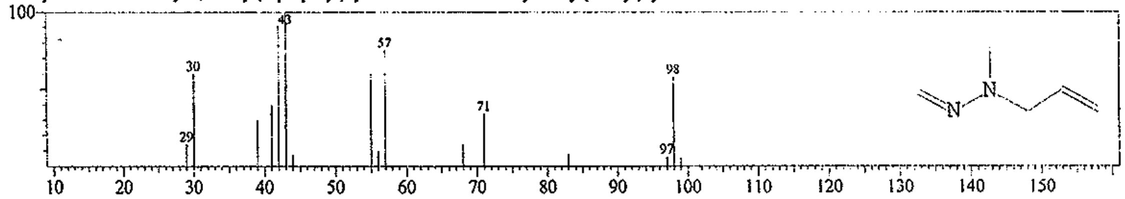

Hit#:3 Entry:1545 Library:NIST05s.LIB
SI:76 Formula:C6H10O CAS:6672-30-6 MolWeight:98 RetIndex:832
CompName:(R)-(+)-3-Methylcyclopentanone $$ Cyclopentanone, 3-methyl-, (R)- $$ 3-Methylcyclopentanone # $$

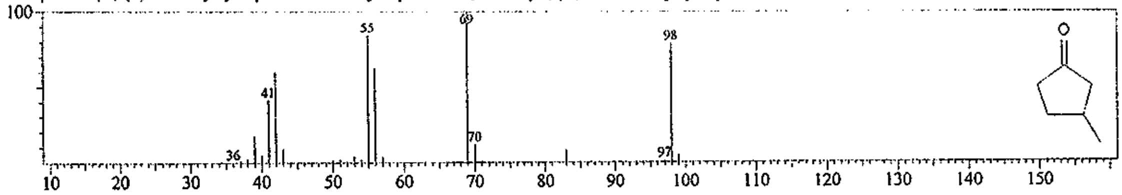

Hit#:4 Entry:73696 Library:NIST05.LIB
SI:76 Formula:C16H30O2 CAS:0-00-0 MolWeight:254 RetIndex:1787
CompName:11-Tridecenyl propionate

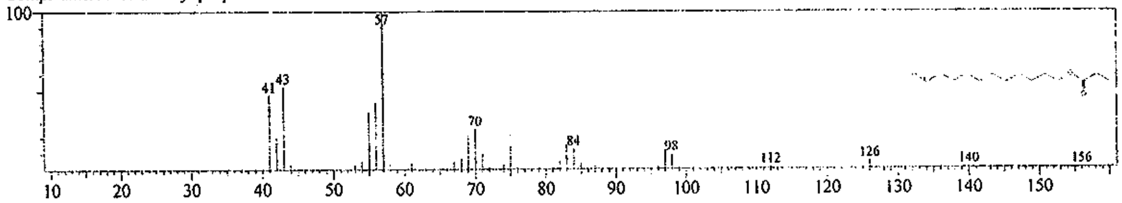

Hit#:5 Entry:12753 Library:NIST05.LIB
SI:75 Formula:C9H20O CAS:18450-74-3 MolWeight:144 RetIndex:1030
CompName:1-Heptanol, 2,4-dimethyl-, (2S,4R)-(-)- $$ 2,4-Dimethyl-1-heptanol # $$

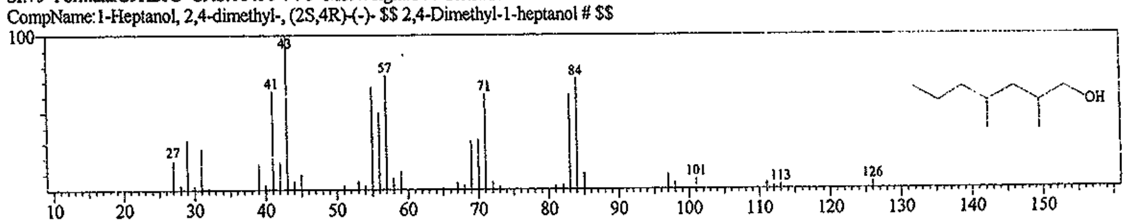

<< Target >>
Line#:6 R.Time:31.633(Scan#:3437) MassPeaks:21
RawMode:Single 31.633(3437) BasePeak:55.10(6908)
BG Mode:31.658(3440) Group 1 - Event 1

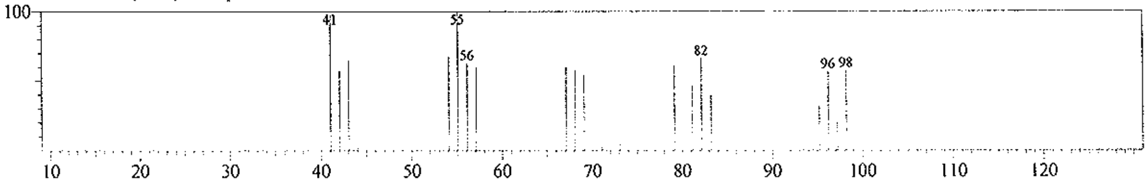

Hit#:1 Entry:10263 Library:NIST05s.LIB
SI:85 Formula:C9H20O2 CAS:3937-56-2 MolWeight:160 RetIndex:1401
CompName:1,9-Nonanediol $$ .alpha.,.omega.-Nonanediol $$

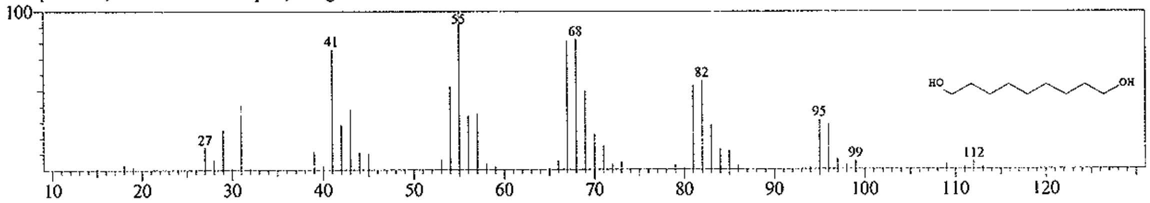

Hit#:2 Entry:12543 Library:NIST05s.LIB
SI:85 Formula:C10H22O2 CAS:112-47-0 MolWeight:174 RetIndex:1501
CompName:1,10-Decanediol $$ Decamethylene glycol $$ Decamethylenediol $$ Decane-1,10-diol $$ 1,10-Decamethylene diol $$ 1,6-Bis(2-hydroxyethyl)

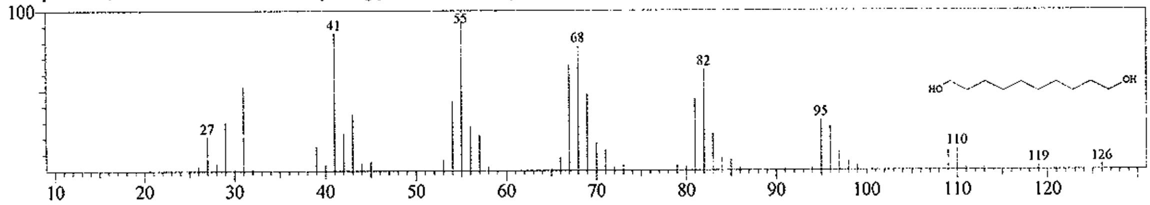

Hit#:3 Entry:59400 Library:NIST05.LIB
SI:85 Formula:C14H30O2 CAS:19812-64-7 MolWeight:230 RetIndex:1898
CompName:1,14-Tetradecanediol $$ tetradecamethylene glycol $$

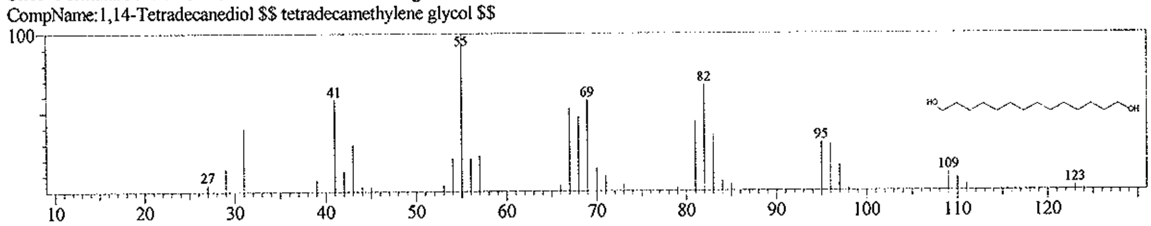

Hit#:4 Entry:10260 Library:NIST05s.LIB
SI:85 Formula:C9H20O2 CAS:3937-56-2 MolWeight:160 RetIndex:1401
CompName:1,9-Nonanediol $$ .alpha.,.omega.-Nonanediol $$

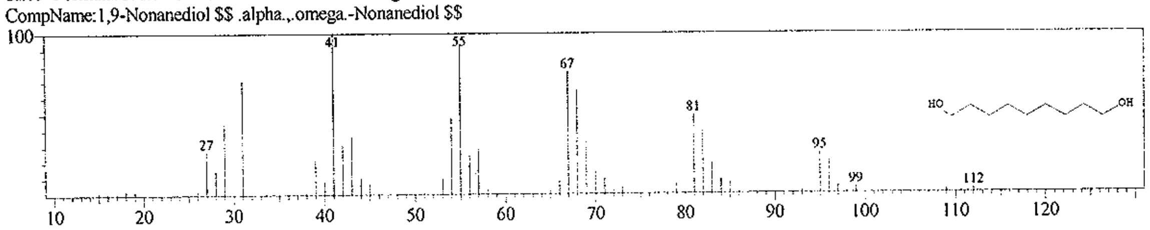

Hit#:5 Entry:19559 Library:NIST05.LIB
SI:84 Formula:C9H20O2 CAS:3937-56-2 MolWeight:160 RetIndex:1401
CompName:1,9-Nonanediol $$ .alpha.,.omega.-Nonanediol $$

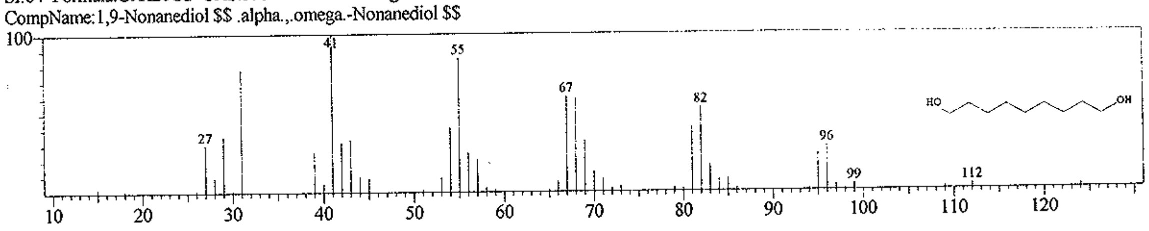

<< Target >>
Line#:7 R.Time:31.942(Scan#:3474) MassPeaks:27
RawMode:Single 31.942(3474) BasePeak:43.05(13734)
BG Mode:32.000(3481) Group 1 - Event 1

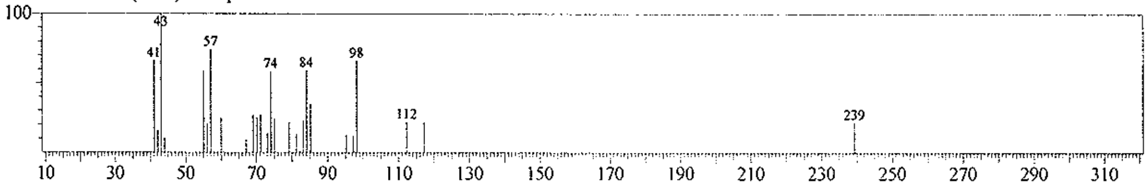

Hit#:1 Entry:117519 Library:NIST05.LIB
SI:82 Formula:C19H38O4 CAS:23470-00-0 MolWeight:330 RetIndex:2498
CompName:Hexadecanoic acid, 2-hydroxy-1-(hydroxymethyl)ethyl ester $$ Palmitin, 2-mono- $$ Palmitic acid .beta.-monoglyceride $$ 2-Hexadecanoyl gl

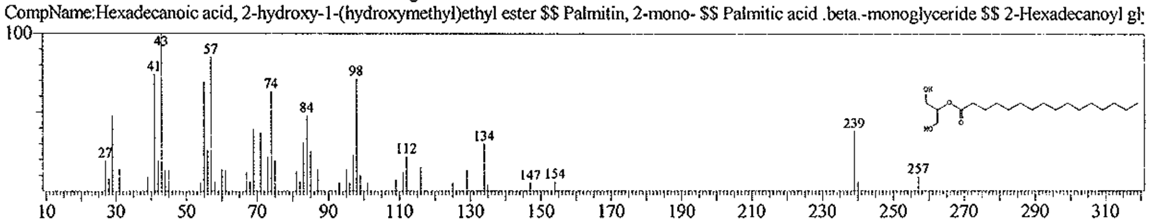

Hit#:2 Entry:117520 Library:NIST05.LIB
SI:82 Formula:C19H38O4 CAS:542-44-9 MolWeight:330 RetIndex:2482
CompName:Hexadecanoic acid, 2,3-dihydroxypropyl ester $$ Palmitin, 1-mono- $$ .alpha.-Monopalmitin $$ Glycerol 1-monopalmitate $$ Glycerol 1-palm

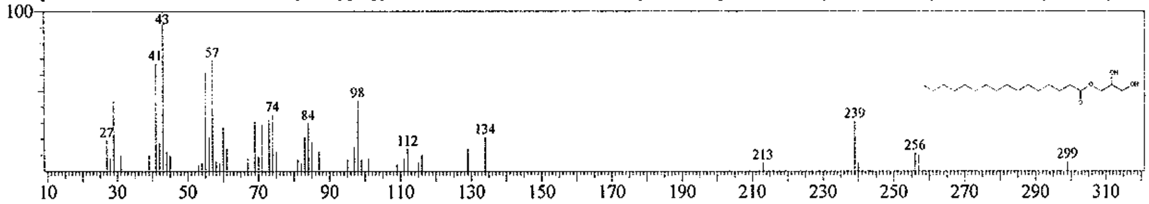

Hit#:3 Entry:110136 Library:NIST05.LIB
SI:81 Formula:C18H36O4 CAS:98863-01-5 MolWeight:316 RetIndex:2399
CompName:Pentadecanoic acid, 2-hydroxy-1-(hydroxymethyl)ethyl ester $$ 2-Hydroxy-1-(hydroxymethyl)ethyl pentadecanoate # $$

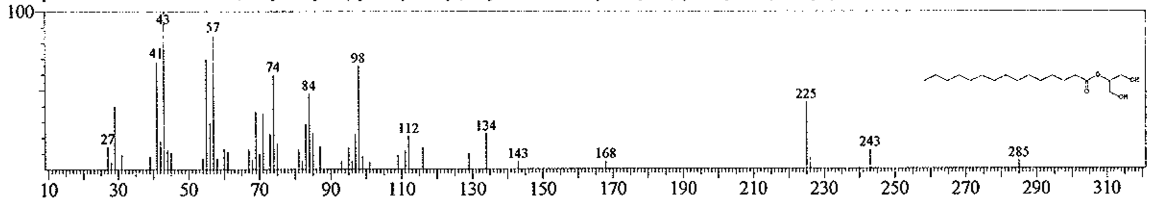

Hit#:4 Entry:162295 Library:NIST05.LIB
SI:81 Formula:C37H74NO8P CAS:3026-45-7 MolWeight:691 RetIndex:0
CompName:Hexadecanoic acid, 1-[[[(2-aminoethoxy)hydroxyphosphinyl]oxy]methyl]-1,2-ethanediyl ester $$ Palmitin, 1,2-di-, 2-aminoethyl hydrogen phos

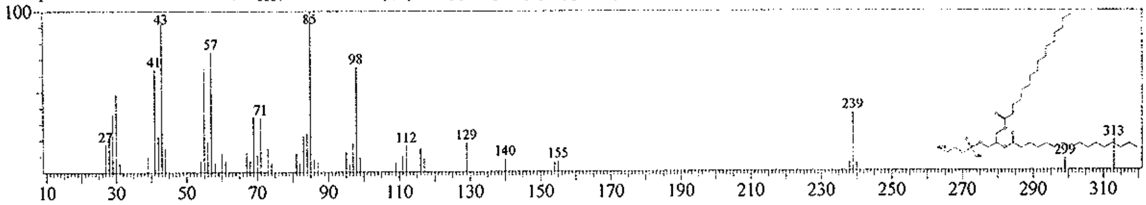

Hit#:5 Entry:117518 Library:NIST05.LIB
SI:81 Formula:C19H38O4 CAS:19670-51-0 MolWeight:330 RetIndex:2482
CompName:Hexadecanoic acid, 2,3-dihydroxypropyl ester, (.+/-.)- $$ 2,3-Dihydroxypropyl palmitate # $$

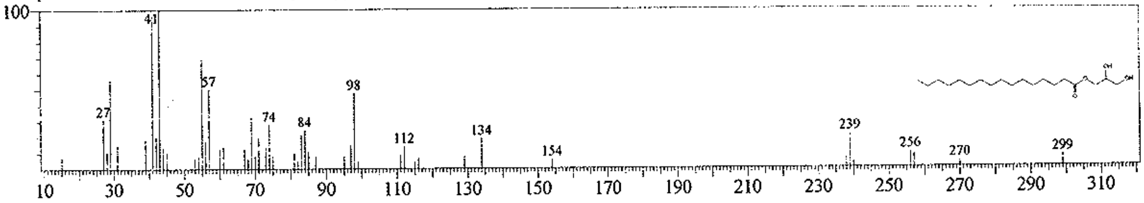

<< Target >>
Line#:8 R.Time:32.367(Scan#:3525) MassPeaks:17
RawMode:Single 32.367(3525) BasePeak:57.10(12405)
BG Mode:32.392(3528) Group 1 - Event 1

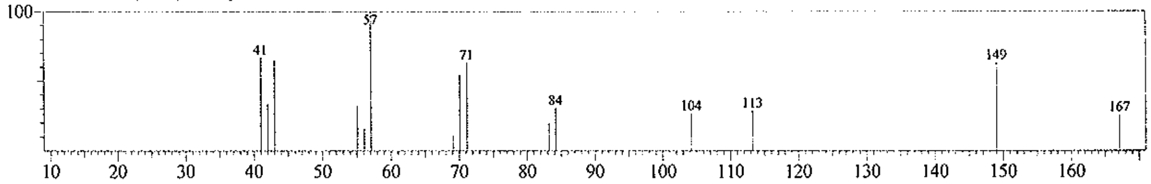

Hit#:1 Entry:36026 Library:NIST05.LIB
SI:79 Formula:C8H17Br CAS:999-64-4 MolWeight:192 RetIndex:1049
CompName:3-Bromooctane $$ Octane, 3-bromo- $$

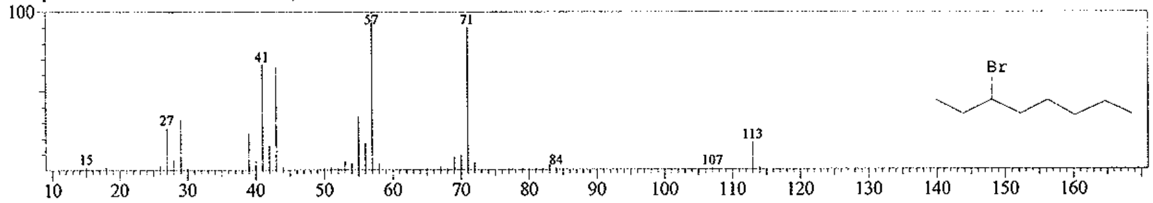

Hit#:2 Entry:36028 Library:NIST05.LIB
SI:79 Formula:C8H17Br CAS:557-35-7 MolWeight:192 RetIndex:1049
CompName:Octane, 2-bromo- $$ sec-Octyl Bromide $$ 1-Methylheptyl bromide $$ 2-Bromooctane $$ 2-Bromoooctane $$ 2-Octyl bromide $$

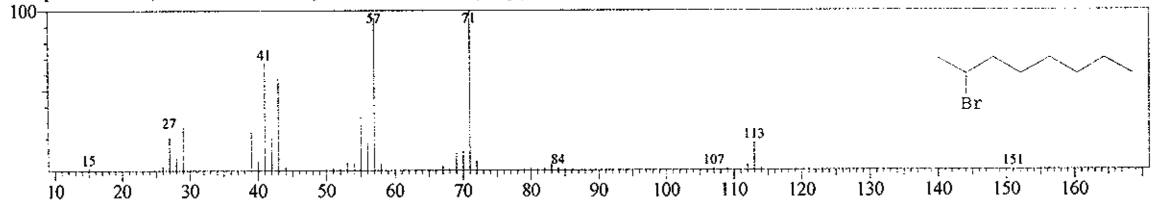

Hit#:3 Entry:14984 Library:NIST05s.LIB
SI:79 Formula:C8H17Br CAS:557-35-7 MolWeight:192 RetIndex:1049
CompName:Octane, 2-bromo- $$ sec-Octyl Bromide $$ 1-Methylheptyl bromide $$ 2-Bromooctane $$ 2-Bromoooctane $$ 2-Octyl bromide $$

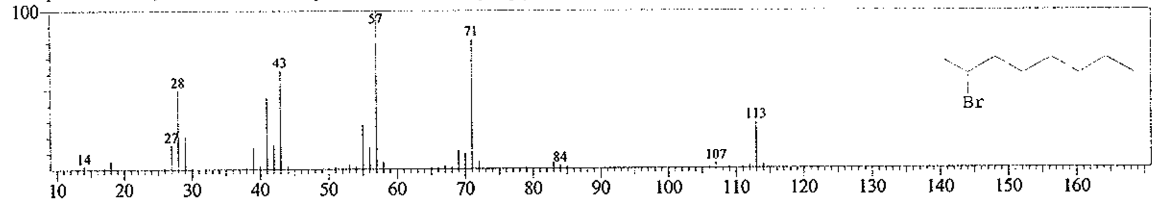

Hit#:4 Entry:20395 Library:NIST05s.LIB
SI:79 Formula:C16H34O CAS:629-82-3 MolWeight:242 RetIndex:1688
CompName:Octane, 1,1'-oxybis- $$ Octyl ether $$ n-Octyl ether $$ Antar $$ Caprylic ether $$ Di-n-octyl ether $$ Dioctyl ether $$ Ether, di-n-octyl- $$ 1-(

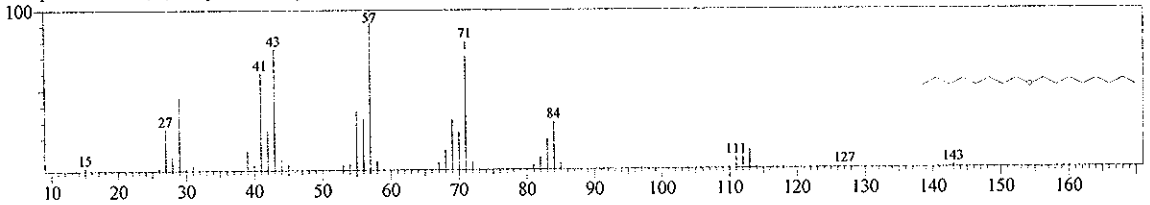

Hit#:5 Entry:66615 Library:NIST05.LIB
SI:78 Formula:C16H34O CAS:629-82-3 MolWeight:242 RetIndex:1688
CompName:Octane, 1,1'-oxybis- $$ Octyl ether $$ n-Octyl ether $$ Antar $$ Caprylic ether $$ Di-n-octyl ether $$ Dioctyl ether $$ Ether, di-n-octyl- $$ 1-(

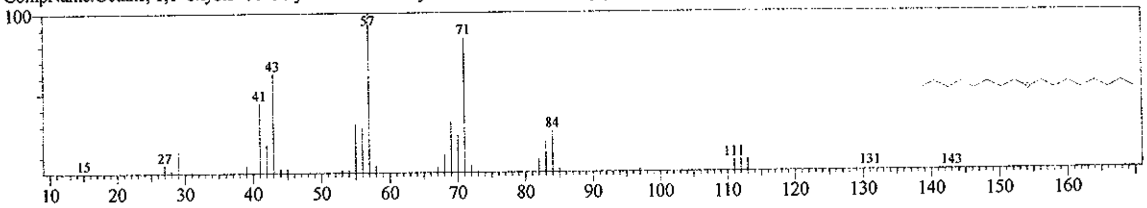

<< Target >>
Line#:9 R.Time:33.875(Scan#:3706) MassPeaks:33
RawMode:Single 33.875(3706) BasePeak:55.10(8970)
BG Mode:33.900(3709) Group 1 - Event 1

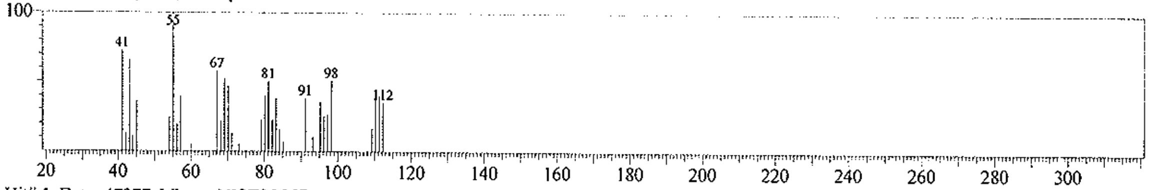

Hit#:1 Entry:47277 Library:NIST05.LIB
SI:85 Formula:C14H26O CAS:85896-31-7 MolWeight:210 RetIndex:1591
CompName:13-Tetradecenal

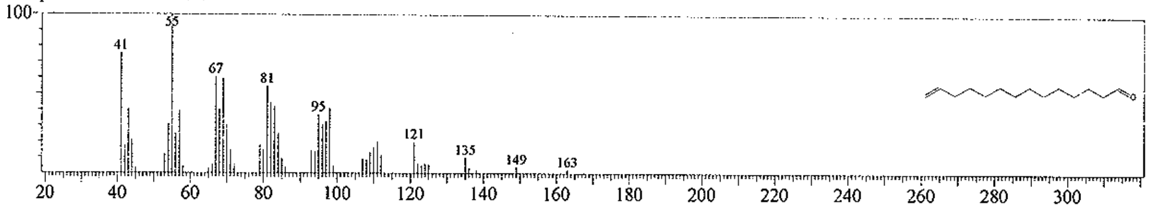

Hit#:2 Entry:73796 Library:NIST05.LIB
SI:85 Formula:C17H34O CAS:30689-78-2 MolWeight:254 RetIndex:1898
CompName:(R)-(-)-(Z)-14-Methyl-8-hexadecen-1-ol $$ (8Z)-14-Methyl-8-hexadecen-1-ol # $$

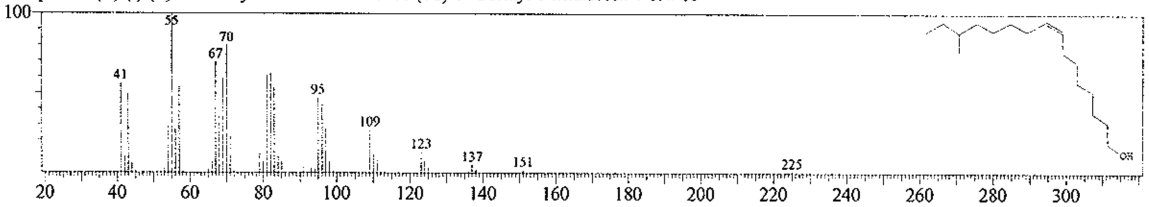

Hit#:3 Entry:47275 Library:NIST05.LIB
SI:85 Formula:C14H26O CAS:65128-96-3 MolWeight:210 RetIndex:1609
CompName:7-Tetradecenal, (Z)- $$ Z-7-Tetradecenal $$ (7Z)-7-Tetradecenal # $$

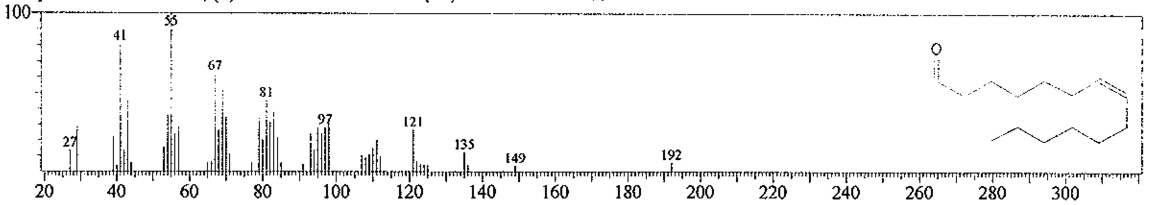

Hit#:4 Entry:121688 Library:NIST05.LIB
SI:85 Formula:C22H42O2 CAS:506-33-2 MolWeight:338 RetIndex:2572
CompName:(E)-13-Docosenoic acid $$ 13-Docosenoic acid, (E)- $$ trans-13-Docosenoic acid $$ Brassidic acid $$ (13E)-13-Docosenoic acid # $$

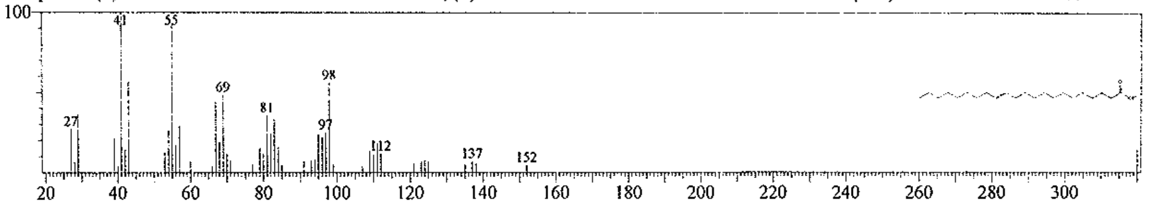

Hit#:5 Entry:20037 Library:NIST05s.LIB
SI:84 Formula:C16H30O CAS:56219-04-6 MolWeight:238 RetIndex:1808
CompName:cis-9-Hexadecenal $$ 9-Hexadecenal, (Z)- $$ (Z)-9-Hexadecenal $$ Z-9-Hexadecenal $$ (9Z)-9-Hexadecenal # $$

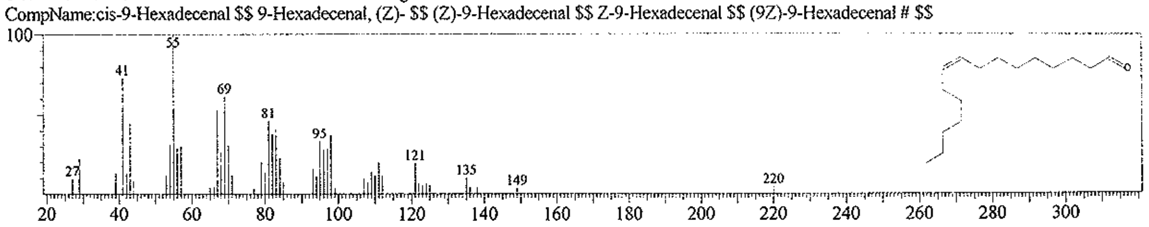

<< Target >>
Line#:10 R.Time:38.308(Scan#:4238) MassPeaks:34
RawMode:Single 38.308(4238) BasePeak:406.30(134255)
BG Mode:38.342(4242) Group 1 - Event 1

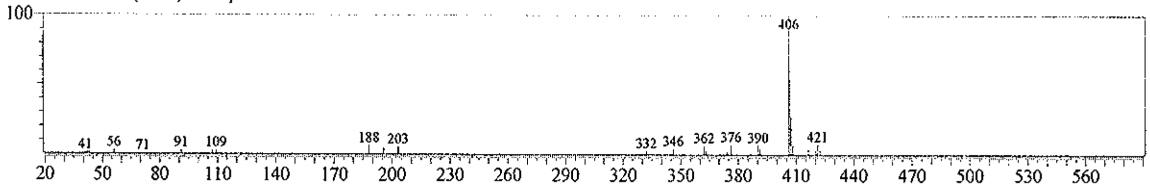

Hit#:1 Entry:148847 Library:NIST05.LIB
SI:64 Formula:C19H31NO4Si3 CAS:55319-89-6 MolWeight:421 RetIndex:2310
CompName:2-Quinolinecarboxylic acid, 4,8-bis[(trimethylsilyl)oxy]-, trimethylsilyl ester $$ Trimethylsilyl 4,8-bis[(trimethylsilyl)oxy]-2-quinolinecarboxyla

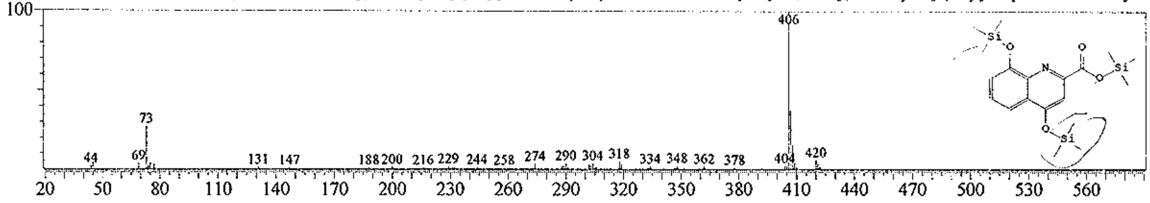

Hit#:2 Entry:160326 Library:NIST05.LIB
SI:64 Formula:C38H70OS CAS:59782-72-8 MolWeight:574 RetIndex:4191
CompName:1-Tetradecanone, 1-(4-methyl-5-nonadecyl-2-thienyl)- $$ 1-(4-Methyl-5-nonadecyl-2-thienyl)-1-tetradecanone # $$

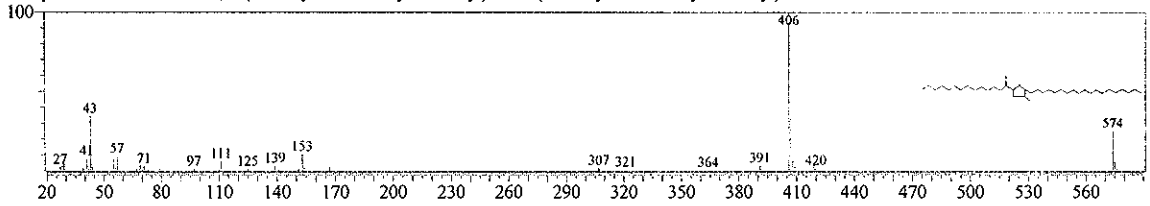

Hit#:3 Entry:154501 Library:NIST05.LIB
SI:60 Formula:C18H22F3N2O3PS2 CAS:0-00-0 MolWeight:466 RetIndex:0
CompName:O,O-Diethyl O-(6-((ethylsulfanyl)methyl)-2-(4-(trifluoromethyl)phenyl)-4-pyrimidinyl) thiophosphate

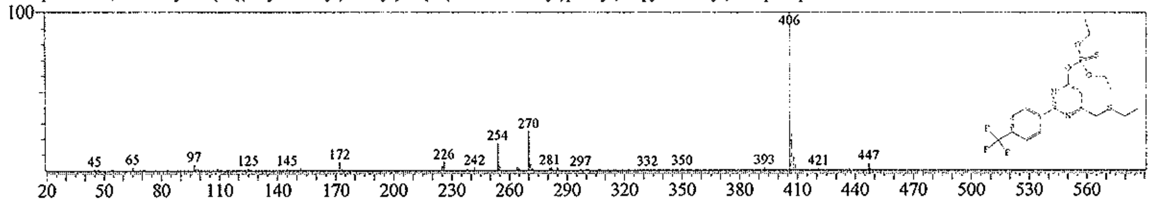

Hit#:4 Entry:146169 Library:NIST05.LIB
SI:60 Formula:C28H38O2 CAS:100807-73-6 MolWeight:406 RetIndex:3235
CompName:2,2'-Dipentyl-3,4,3',4'-tetrahydro-2H,2'H-[6,6']bichromenyl

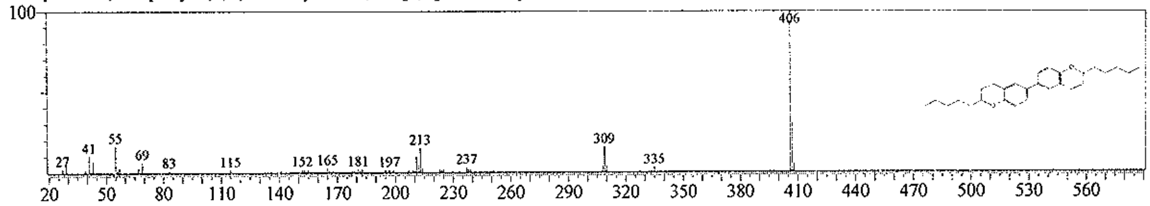

Hit#:5 Entry:160514 Library:NIST05.LIB
SI:59 Formula:C33H33FeO6 CAS:15713-87-8 MolWeight:581 RetIndex:0
CompName:Tris(3-phenyl-2,4-pentanedionato)iron(iii)

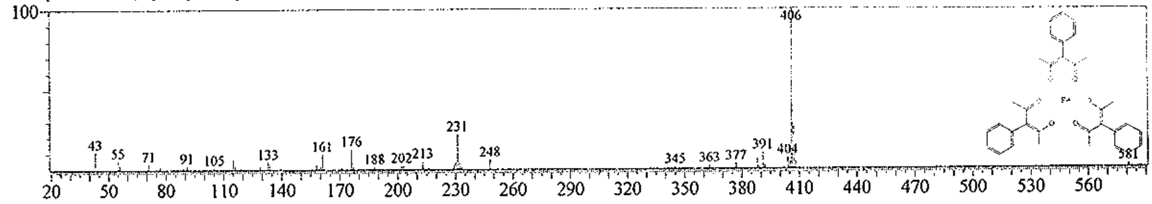

<< Target >>
Line#:11 R.Time:38.500(Scan#:4261) MassPeaks:16
RawMode:Single 38.500(4261) BasePeak:420.30(52495)
BG Mode:38.533(4265) Group 1 - Event 1

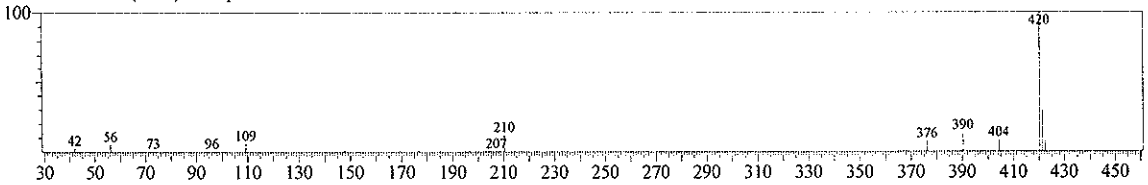

Hit#:1 Entry:148790 Library:NIST05.LIB
SI:73 Formula:C28H24N2O2 CAS:10123-03-2 MolWeight:420 RetIndex:3749
CompName:N,N'-Bis(p-methoxybenzylidene)benzidine

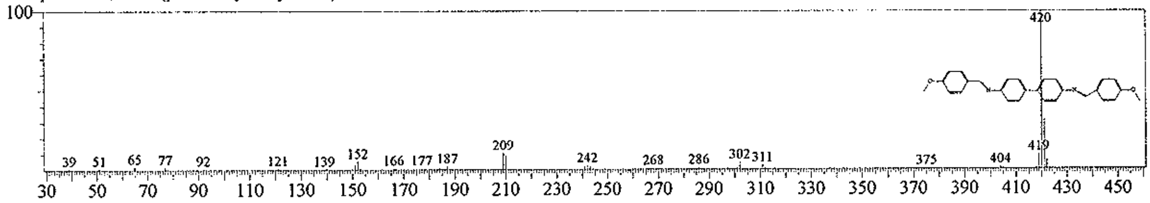

Hit#:2 Entry:148781 Library:NIST05.LIB
SI:68 Formula:C28H18F2N2 CAS:22158-34-5 MolWeight:420 RetIndex:3365
CompName:Pyrazine, 2,5-bis(p-fluorophenyl)-3,6-diphenyl- $$ 2,5-Bis(4-fluorophenyl)-3,6-diphenylpyrazine # $$

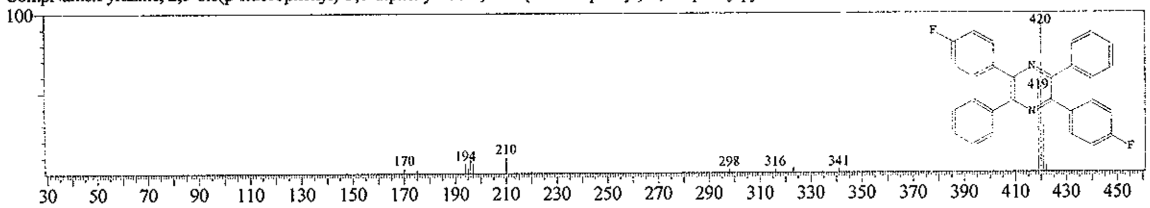

Hit#:3 Entry:148791 Library:NIST05.LIB
SI:63 Formula:C28H24N2O2 CAS:16196-93-3 MolWeight:420 RetIndex:3749
CompName:N,N'-Dibenzylidene-3,3'-dimethoxybenzidine

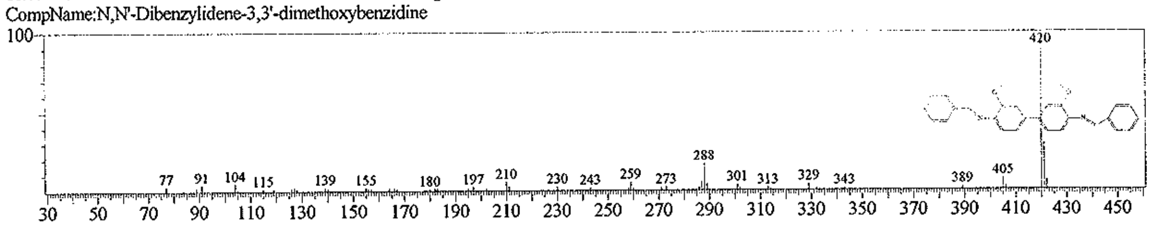

Hit#:4 Entry:148793 Library:NIST05.LIB
SI:58 Formula:C28H28N4 CAS:0-00-0 MolWeight:420 RetIndex:3759
CompName:1,5-Naphthyldiamine, N,N'-bis[4-(dimethylamino)benzylidene]-

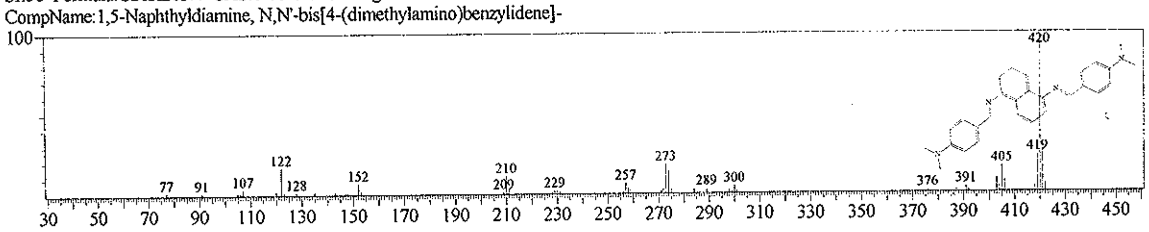

Hit#:5 Entry:153005 Library:NIST05.LIB
SI:55 Formula:C23H17NO9 CAS:37914-17-3 MolWeight:451 RetIndex:3386
CompName:4H-Pyrido[3,2,1-jk]carbazole-1,2,3,6-tetracarboxylic acid, 4-oxo-, tetramethyl ester $$ Tetramethyl 4-oxo-4H-pyrido[3,2,1-jk]carbazole-1,2,3,6

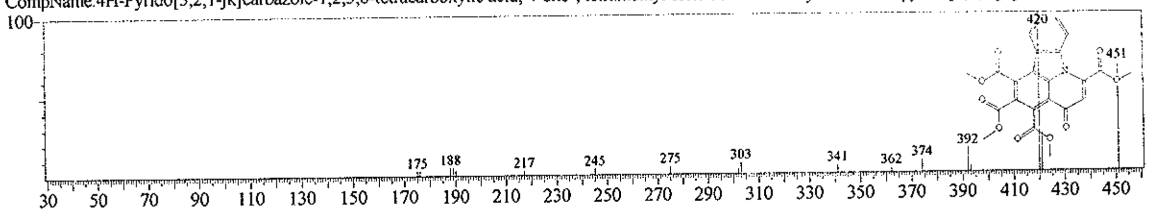

<< Target >>
Line#:12 R.Time:39.283(Scan#:4355) MassPeaks:31
RawMode:Single 39.283(4355) BasePeak:392.30(67066)
BG Mode:39.325(4360) Group 1 - Event 1

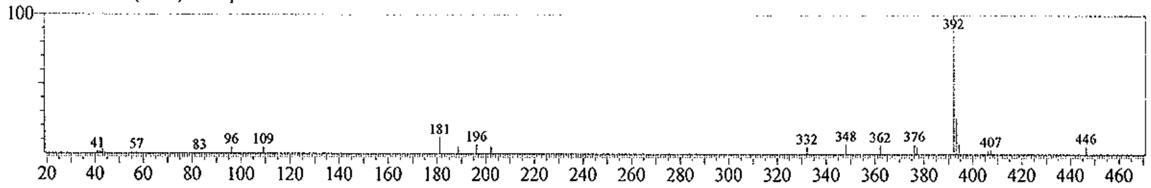

Hit#:1 Entry:146259 Library:NIST05.LIB
SI:72 Formula:C24H25NO5 CAS:0-00-0 MolWeight:407 RetIndex:3792
CompName:N-Formylkorupensamin b

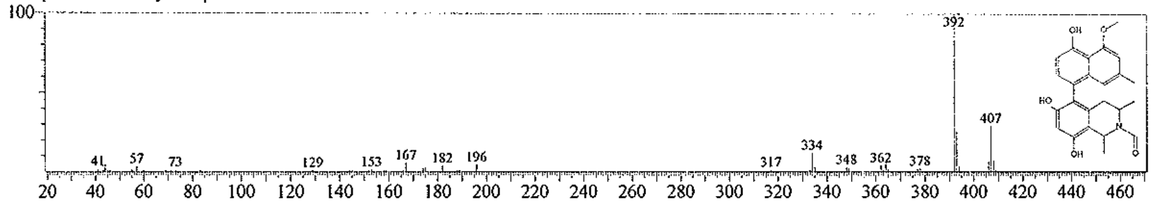

Hit#:2 Entry:142719 Library:NIST05.LIB
SI:67 Formula:C27H24N2O CAS:0-00-0 MolWeight:392 RetIndex:3428
CompName:1H-Pyrrolo[3,2-g]quinoline, 9-methoxy-1,2,3-trimethyl-5,7-diphenyl-

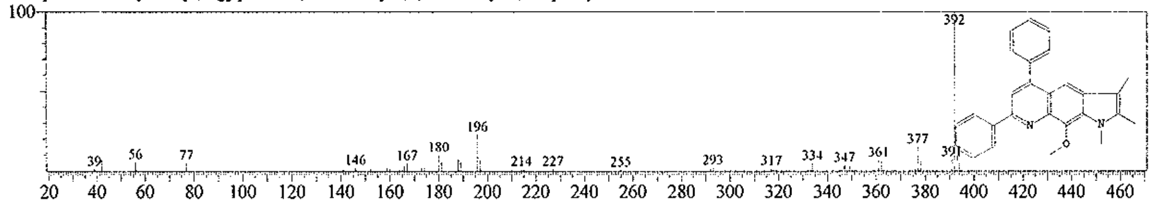

Hit#:3 Entry:154118 Library:NIST05.LIB
SI:63 Formula:C27H50F3NO CAS:65623-32-7 MolWeight:461 RetIndex:3300
CompName:1-(Trifluoroacetyl)azacyclohexacosane $$ Azacyclohexacosane, 1-(trifluoroacetyl)- $$

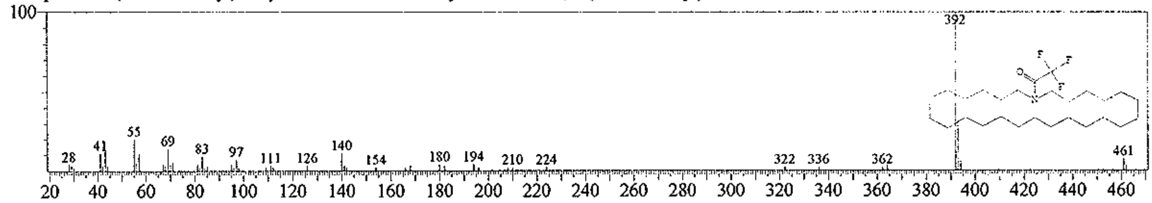

Hit#:4 Entry:142737 Library:NIST05.LIB
SI:61 Formula:C31H20 CAS:86623-52-1 MolWeight:392 RetIndex:3690
CompName:Perylene, 3-(2-naphthalenylmethyl)- $$ 3-(2-Naphthylmethyl)perylene # $$

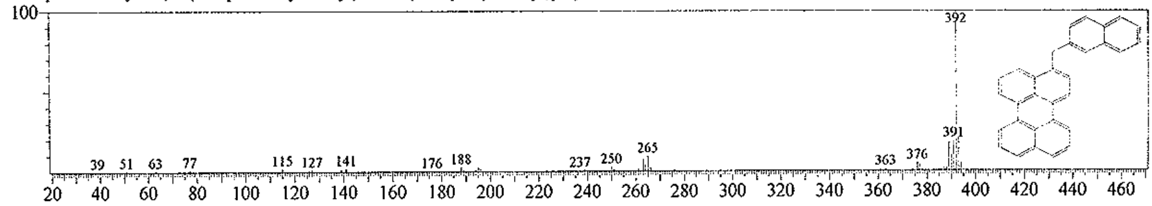

Hit#:5 Entry:142480 Library:NIST05.LIB
SI:60 Formula:C16H13IN2S CAS:330682-81-0 MolWeight:392 RetIndex:2861
CompName:2-[(3-Iodo-benzylidene)-amino]-4,5,6,7-tetrahydro-benzo[b]thiophene-3-carbonitrile $$ 2-([(E)-(3-Iodophenyl)methylidene]amino)-4,5,6,7-tetr

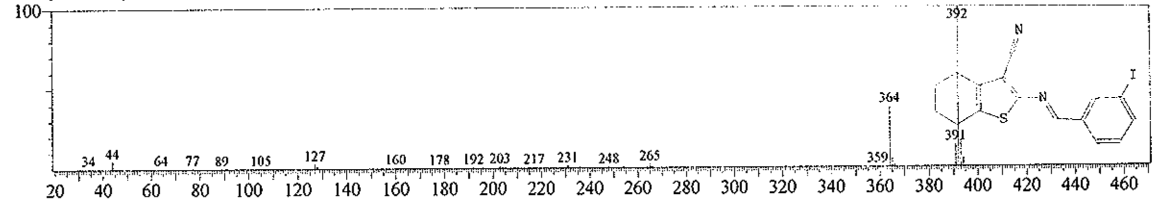

<< Target >>
Line#:13 R.Time:39.383(Scan#:4367) MassPeaks:19
RawMode:Single 39.383(4367) BasePeak:392.30(56381)
BG Mode:39.350(4363) Group 1 - Event 1

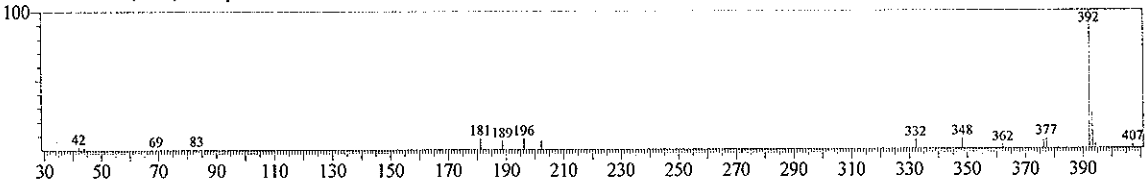

Hit#:1 Entry:146259 Library:NIST05.LIB
SI:72 Formula:C24H25NO5 CAS:0-00-0 MolWeight:407 RetIndex:3792
CompName:N-Formylkorupensamin b

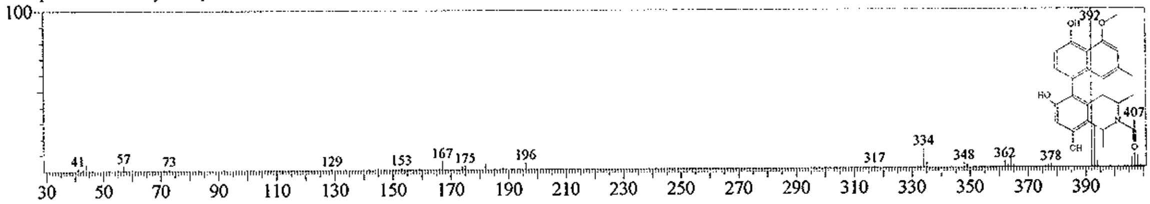

Hit#:2 Entry:142719 Library:NIST05.LIB
SI:70 Formula:C27H24N2O CAS:0-00-0 MolWeight:392 RetIndex:3428
CompName:1H-Pyrrolo[3,2-g]quinoline, 9-methoxy-1,2,3-trimethyl-5,7-diphenyl-

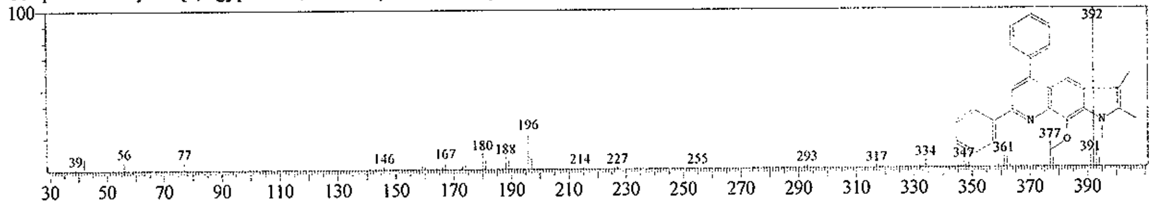

Hit#:3 Entry:142737 Library:NIST05.LIB
SI:64 Formula:C31H20 CAS:86623-52-1 MolWeight:392 RetIndex:3690
CompName:Perylene, 3-(2-naphthalenylmethyl)- $$ 3-(2-Naphthylmethyl)perylene # $$

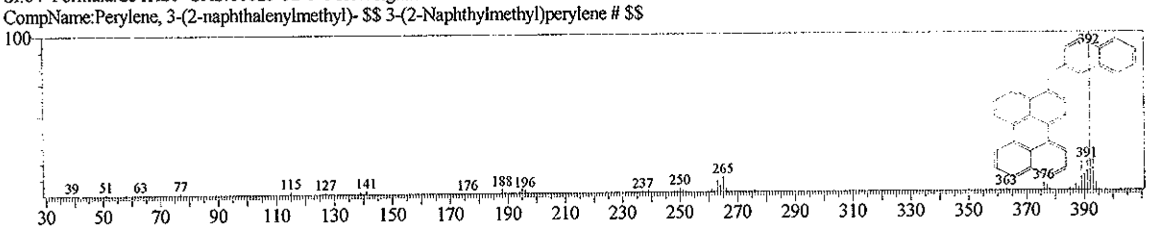

Hit#:4 Entry:142480 Library:NIST05.LIB
SI:64 Formula:C16H13IN2S CAS:330682-81-0 MolWeight:392 RetIndex:2861
CompName:2-[(3-Iodo-benzylidene)-amino]-4,5,6,7-tetrahydro-benzo[b]thiophene-3-carbonitrile $$ 2-{[(E)-(3-Iodophenyl)methylidene]amino}-4,5,6,7-tetr

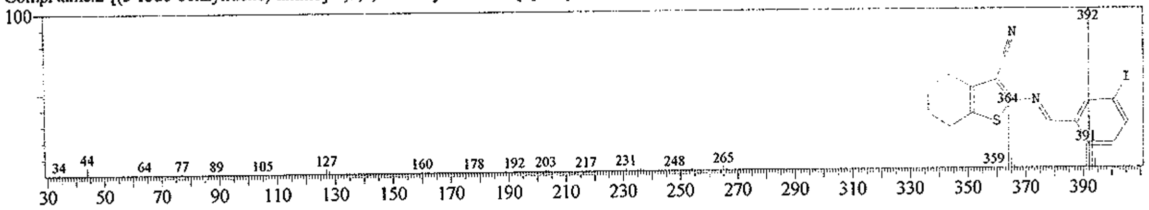

Hit#:5 Entry:142450 Library:NIST05.LIB
SI:61 Formula:C14H11F7O5 CAS:51145-23-4 MolWeight:392 RetIndex:1484
CompName:4-Heptafluorobutyryloxy-3-methoxybenzeneacetic acid, methyl ester $$ 2-Methoxy-4-(2-methoxy-2-oxoethyl)phenyl 2,2,3,3,4,4,4-heptafluorob

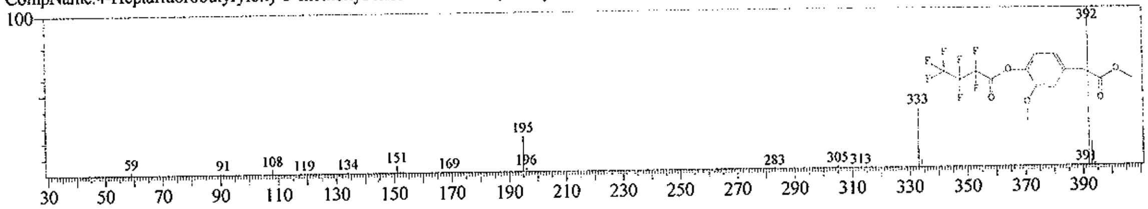

<< Target >>
Line#:14 R.Time:39.825(Scan#:4420) MassPeaks:15
RawMode:Single 39.825(4420) BasePeak:392.30(24520)
BG Mode:39.792(4416) Group 1 - Event 1

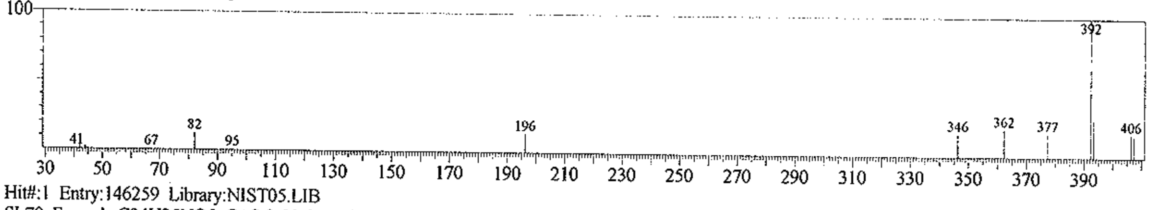

Hit#:1 Entry:146259 Library:NIST05.LIB
SI:70 Formula:C24H25NO5 CAS:0-00-0 MolWeight:407 RetIndex:3792
CompName:N-Formylkorupensamin b

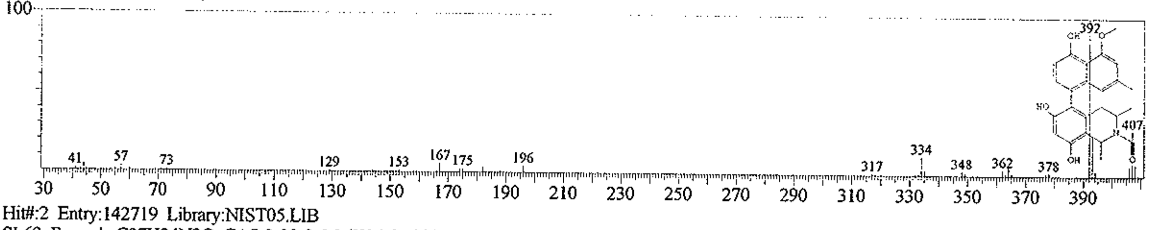

Hit#:2 Entry:142719 Library:NIST05.LIB
SI:62 Formula:C27H24N2O CAS:0-00-0 MolWeight:392 RetIndex:3428
CompName:1H-Pyrrolo[3,2-g]quinoline, 9-methoxy-1,2,3-trimethyl-5,7-diphenyl-

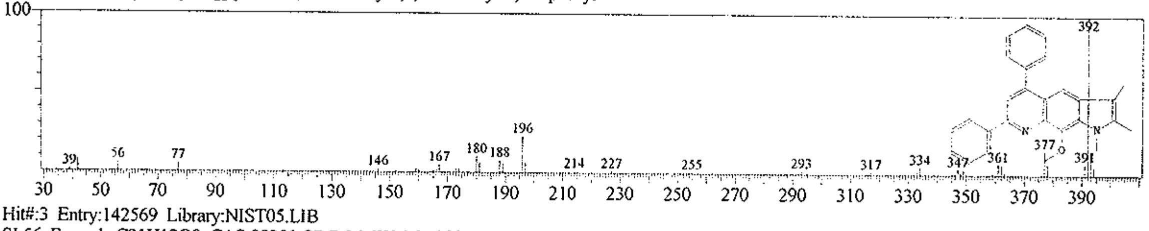

Hit#:3 Entry:142569 Library:NIST05.LIB
SI:56 Formula:C21H12O8 CAS:88381-87-7 MolWeight:392 RetIndex:3678
CompName:3,5,5'-Trihydroxy-3'-methoxy-2,2'-binaphthalene-1,1',4,4'-tetrone

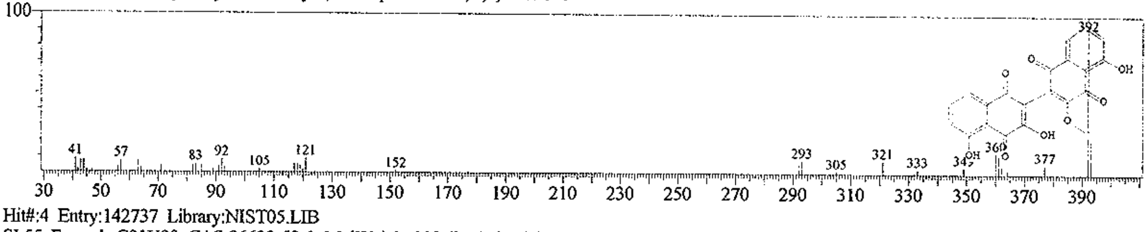

Hit#:4 Entry:142737 Library:NIST05.LIB
SI:55 Formula:C31H20 CAS:86623-52-1 MolWeight:392 RetIndex:3690
CompName:Perylene, 3-(2-naphthalenylmethyl)- $$ 3-(2-Naphthylmethyl)perylene # $$

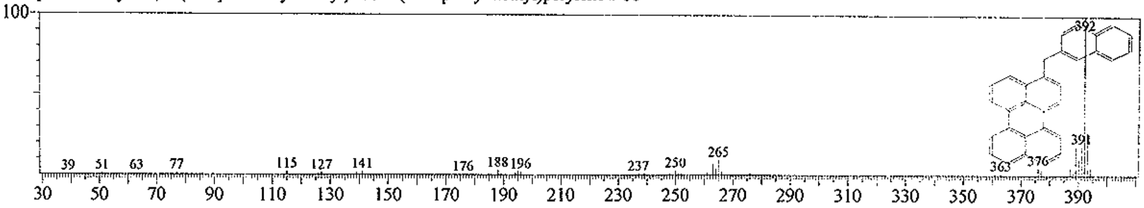

Hit#:5 Entry:142480 Library:NIST05.LIB
SI:55 Formula:C16H13IN2S CAS:330682-81-0 MolWeight:392 RetIndex:2861
CompName:2-[(3-Iodo-benzylidene)-amino]-4,5,6,7-tetrahydro-benzo[b]thiophene-3-carbonitrile $$ 2-([(E)-(3-Iodophenyl)methylidene]amino)-4,5,6,7-tetr

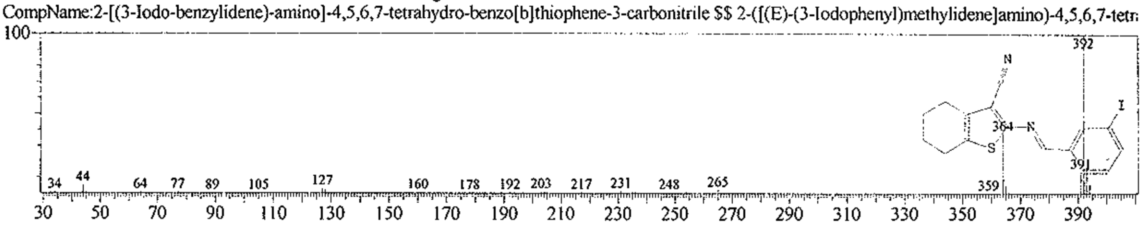

<< Target >>
Line#:15 R.Time:40.225(Scan#:4468) MassPeaks:16
RawMode:Single 40.225(4468) BasePeak:392.30(33293)
BG Mode:40.258(4472) Group 1 - Event 1

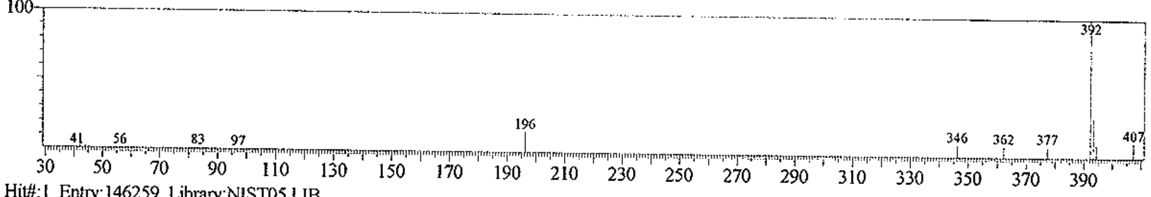

Hit#:1 Entry:146259 Library:NIST05.LIB
SI:73 Formula:C24H25NO5 CAS:0-00-0 MolWeight:407 RetIndex:3792
CompName:N-Formylkorupensamin b

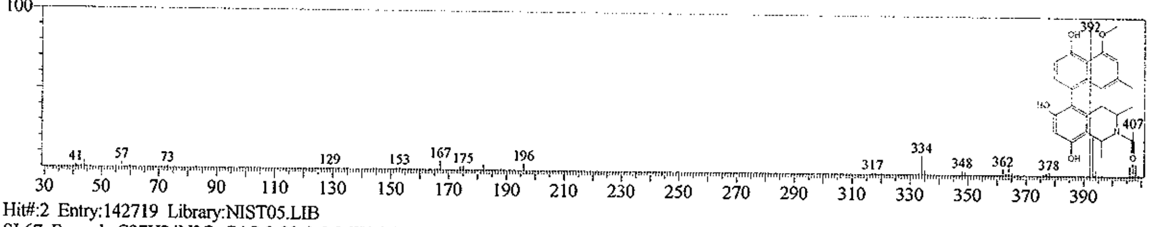

Hit#:2 Entry:142719 Library:NIST05.LIB
SI:67 Formula:C27H24N2O CAS:0-00-0 MolWeight:392 RetIndex:3428
CompName:1H-Pyrrolo[3,2-g]quinoline, 9-methoxy-1,2,3-trimethyl-5,7-diphenyl-

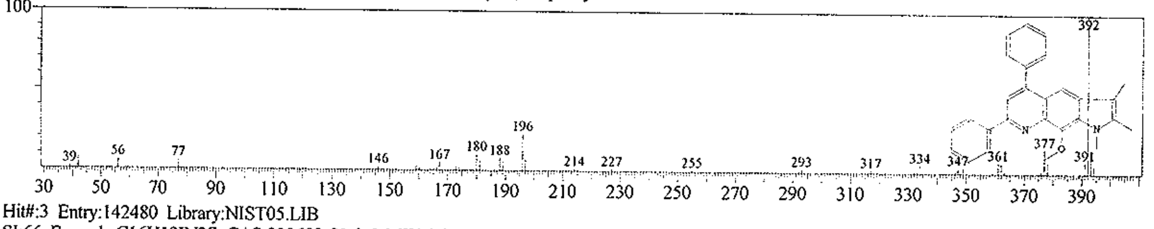

Hit#:3 Entry:142480 Library:NIST05.LIB
SI:66 Formula:C16H13IN2S CAS:330682-81-0 MolWeight:392 RetIndex:2861
CompName:2-[(3-Iodo-benzylidene)-amino]-4,5,6,7-tetrahydro-benzo[b]thiophene-3-carbonitrile $$ 2-([(E)-(3-Iodophenyl)methylidene]amino)-4,5,6,7-tetr

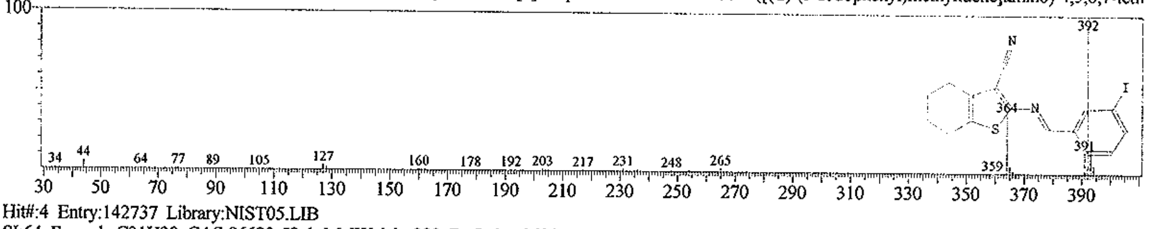

Hit#:4 Entry:142737 Library:NIST05.LIB
SI:64 Formula:C31H20 CAS:86623-52-1 MolWeight:392 RetIndex:3690
CompName:Perylene, 3-(2-naphthalenylmethyl)- $$ 3-(2-Naphthylmethyl)perylene # $$

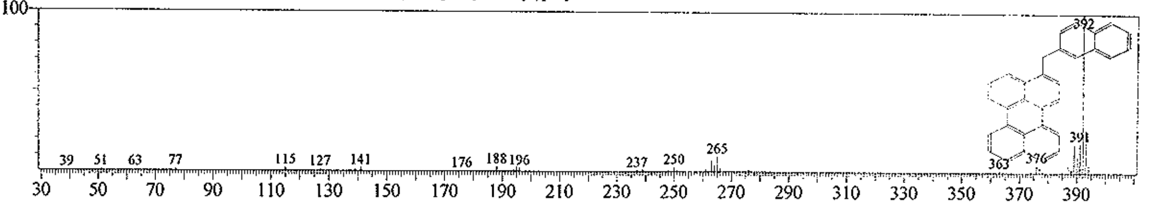

Hit#:5 Entry:142450 Library:NIST05.LIB
SI:63 Formula:C14H11F7O5 CAS:51145-23-4 MolWeight:392 RetIndex:1484
CompName:4-Heptafluorobutyryloxy-3-methoxybenzeneacetic acid, methyl ester $$ 2-Methoxy-4-(2-methoxy-2-oxoethyl)phenyl 2,2,3,3,4,4,4-heptafluorob

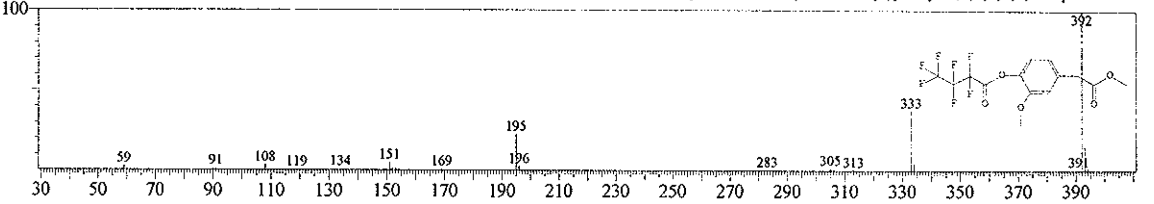

<< Target >>
Line#:16 R.Time:41.967(Scan#:4677) MassPeaks:20
RawMode:Single 41.967(4677) BasePeak:91.20(3279)
BG Mode:41.983(4679) Group 1 - Event 1

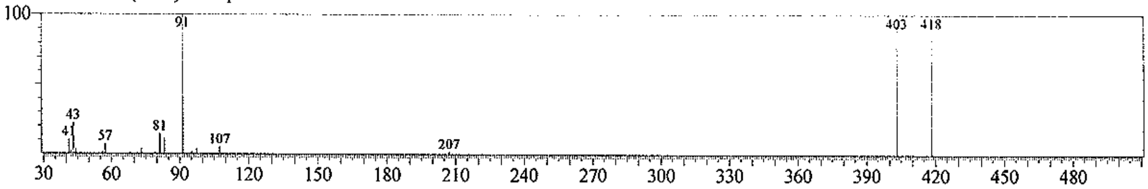

Hit#:1 Entry:153994 Library:NIST05.LIB
SI:60 Formula:C26H20O8 CAS:104505-99-9 MolWeight:460 RetIndex:3926
CompName:4-Acetoxy-6',7-dimethyl-5',8'-dimethoxy-1,2'-binaphthalene-1',4',5,8-tetrone

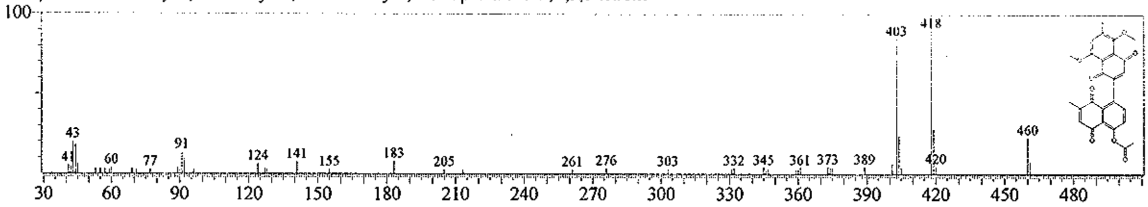

Hit#:2 Entry:145506 Library:NIST05.LIB
SI:60 Formula:C24H18FNO4 CAS:141523-17-3 MolWeight:403 RetIndex:3292
CompName:4,5-Dihydrooxazol-5-one, 4-[4-benzyloxy-2-fluoro-5-methoxybenzylidene]- $$ (4Z)-4-[4-(Benzyloxy)-2-fluoro-5-methoxybenzylidene]-2-phen

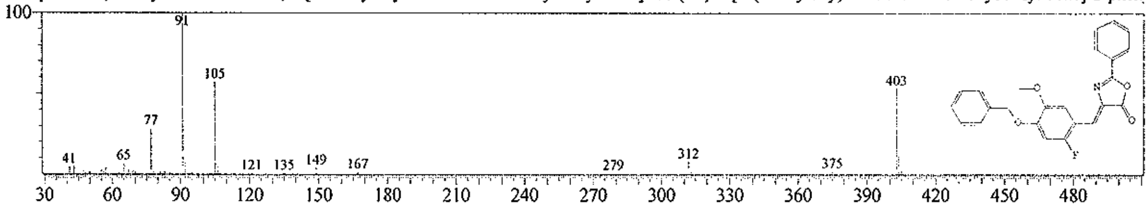

Hit#:3 Entry:157785 Library:NIST05.LIB
SI:57 Formula:C33H35NO4 CAS:0-00-0 MolWeight:509 RetIndex:4051
CompName:Tetrabenzylarabinofuranose

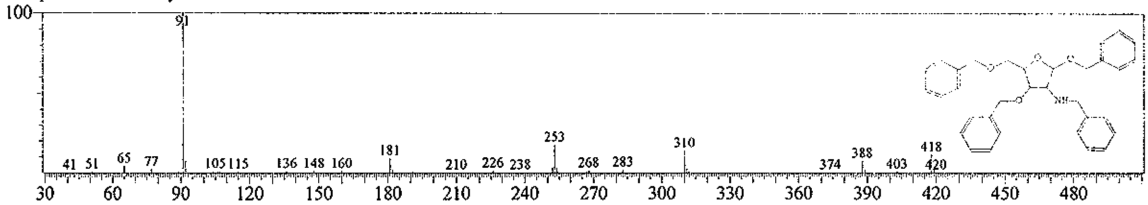

Hit#:4 Entry:148396 Library:NIST05.LIB
SI:57 Formula:C26H26O5 CAS:0-00-0 MolWeight:418 RetIndex:3354
CompName:2,3,5-O,O,O-Tribenzyl-arabinolactone

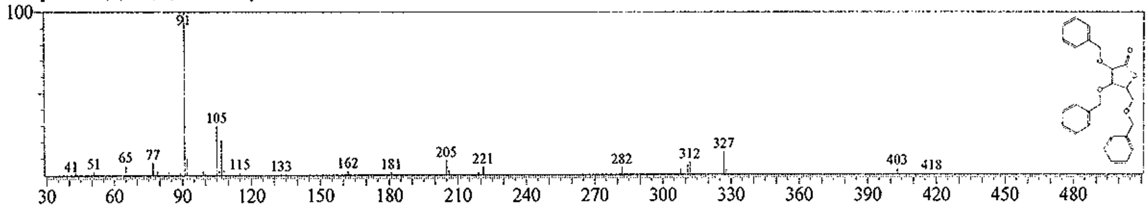

Hit#:5 Entry:148367 Library:NIST05.LIB
SI:57 Formula:C25H22O6 CAS:95817-41-7 MolWeight:418 RetIndex:3378
CompName:7-Benzyloxy-3-methoxy-2-(3,4-dimethoxyphenyl)-4H-chromen-4-one $$ 7-(Benzyloxy)-2-(3,4-dimethoxyphenyl)-3-methoxy-4H-chromen-4-or

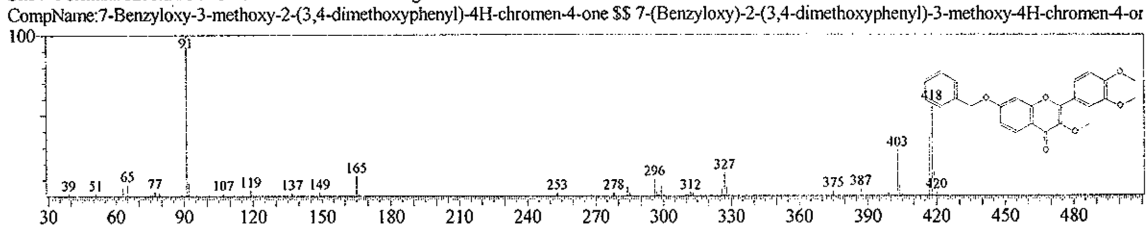

<< Target >>
Line#:20 R.Time:39.575(Scan#:4390) MassPeaks:20
RawMode:Single 39.575(4390) BasePeak:406.30(16421)
BG Mode:39.592(4392) Group 1 - Event 1

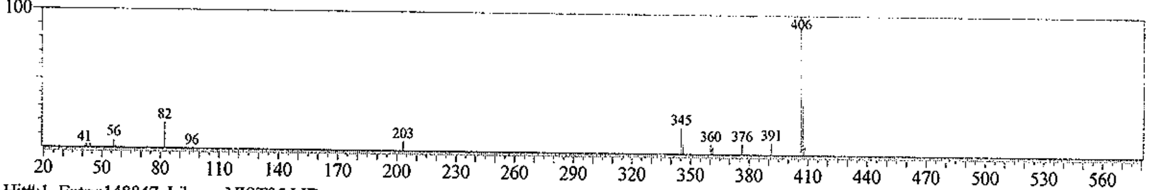

Hit#:1 Entry:148847 Library:NIST05.LIB
SI:61 Formula:C19H31NO4Si3 CAS:55319-89-6 MolWeight:421 RetIndex:2310
CompName:2-Quinolinecarboxylic acid, 4,8-bis[(trimethylsilyl)oxy]-, trimethylsilyl ester $$ Trimethylsilyl 4,8-bis[(trimethylsilyl)oxy]-2-quinolinecarboxyla

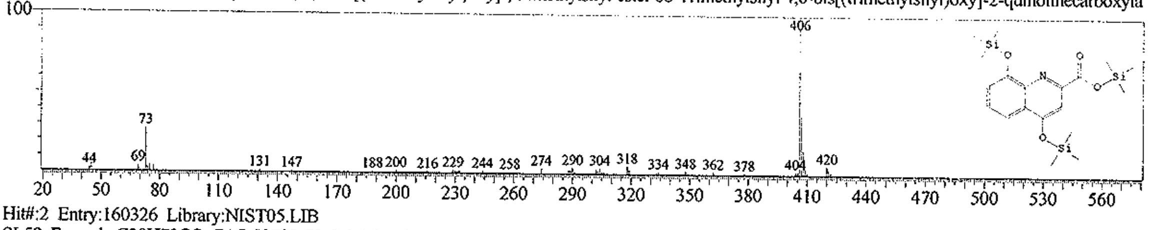

Hit#:2 Entry:160326 Library:NIST05.LIB
SI:59 Formula:C38H70OS CAS:59782-72-8 MolWeight:574 RetIndex:4191
CompName:1-Tetradecanone, 1-(4-methyl-5-nonadecyl-2-thienyl)- $$ 1-(4-Methyl-5-nonadecyl-2-thienyl)-1-tetradecanone # $$

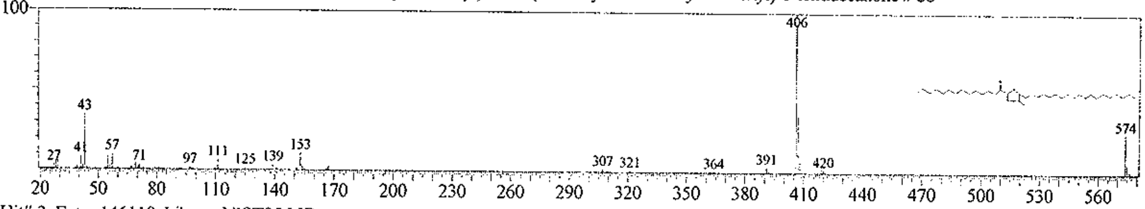

Hit#:3 Entry:146118 Library:NIST05.LIB
SI:57 Formula:C24H26N2O4 CAS:808-24-2 MolWeight:406 RetIndex:2960
CompName:Nicodicodine $$ Morphinan-6-ol, 4,5-epoxy-3-methoxy-17-methyl-, 3-pyridinecarboxylate (ester), (5.alpha.,6.alpha.)- $$ Morphinan-6.alpha.-o!

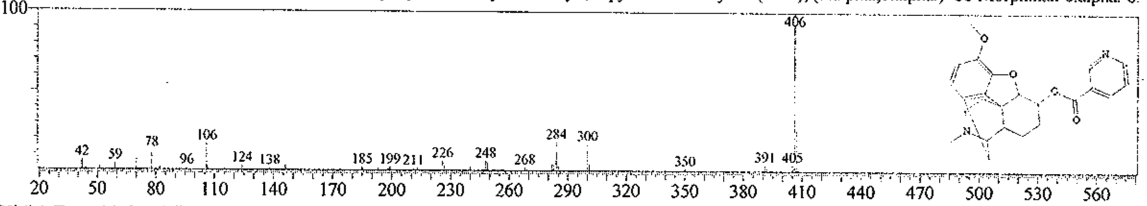

Hit#:4 Entry:145955 Library:NIST05.LIB
SI:57 Formula:C12H8I2 CAS:3001-15-8 MolWeight:406 RetIndex:2175
CompName:4,4'-Diiododiphenyl $$ 1,1'-Biphenyl, 4,4'-diiodo- $$ 4,4'-Diiodo-1,1'-biphenyl # $$

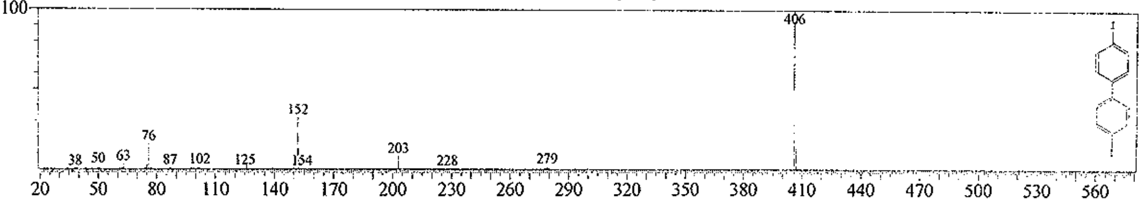

Hit#:5 Entry:146169 Library:NIST05.LIB
SI:57 Formula:C28H38O2 CAS:100807-73-6 MolWeight:406 RetIndex:3235
CompName:2,2'-Dipentyl-3,4,3',4'-tetrahydro-2H,2'H-[6,6']bichromenyl

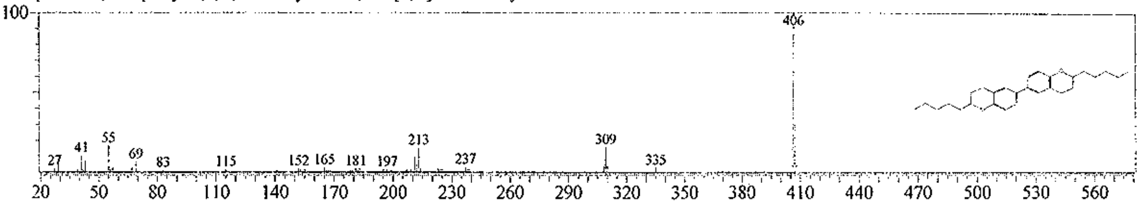

Supplement: Additional file 3 — Gas chromatography-mass spectrometry result of the stem bark of Ancistrocladus uncinatus. [file 1746-6148-9-120-S3.pdf]
