# Supplementary material for: Phytochemical analysis and in-vitro anti-African swine fever virus activity of extracts and fractions of Ancistrocladus uncinatus, Hutch and Dalziel (Ancistrocladaceae)
Source: BMC Vet Res. 2013 Jun 19;9:120. doi: 10.1186/1746-6148-9-120 (PMC3694037; doi:10.1186/1746-6148-9-120)
Supplement: Additional file 4 — Preparation of Primary Bone Marrow Cultures. [file 1746-6148-9-120-S4.doc]

AGRICULTURAL RESEARCH COUNCIL

Onderstepoort Veterinary Institute (OVI)

Exotic Diseases Division (EDD)

## Standard Operating Procedure:

### Preparation of Primary Bone Marrow Cultures

SOP Number: R&D ASF 04-00

Date revised

**Prepared by:**

Name: B.A Lubisi Research Veterinarian 16 May 2005

**Reviewed by:**

Name: Position: Date:

**Approved by:**

Name: Position: Date:

Date issued:

## Table of content

1. SCOPE AND APPLICATION: [3](#__RefHeading___Toc91040148)

2. DEFINITIONS/ABBREVIATIONS: [3](#__RefHeading___Toc91040149)

3. HEALTH AND SAFETY: [3](#__RefHeading___Toc91040150)

4. CAUTIONS: [3](#__RefHeading___Toc91040151)

5. REAGENTS AND SOLUTIONS: [3](#__RefHeading___Toc91040152)

6. APPARATUS AND MATERIALS: [4](#__RefHeading___Toc91040153)

7. PROCEDURE AND ANALYSIS [5](#__RefHeading___Toc91040154)

8. DATA ANALYSIS/CALCULATIONS: [6](#__RefHeading___Toc91040155)

9. QUALITY CONTROL: [7](#__RefHeading___Toc91040156)

10. REFERENCES: [7](#__RefHeading___Toc91040158)

11. FORMS AND DATA SHEET: [7](#__RefHeading___Toc91040159)

# SCOPE AND APPLICATION:

1.1 The purpose of this SOP is to provide guidance for the preparation of primary bone marrow cultures at the EDD laboratory.

1.2 This procedure is intended for use by the laboratory personnel assigned to prepare primary bone marrow cultures for isolation of the African swine fever virus.

# 2. DEFINITIONS/ABBREVIATIONS:

2.1 Haemadsorption: The attachment of red blood cells to macrophages. This can be seen microscopically as rosette formation.

2.2 Cyto-pathic effect: Damage of cells by microorganisms. This effect on macrophages can be seen microscopically as a decrease in number of macrophages.

2.3 ASF African Swine Fever

2.4 CPE Cytopathic effect

2.5 EDD

2.6 Had Haemadsorption

2.7 PBS Phosphate Buffered Saline

# 3. HEALTH AND SAFETY:

3.1 Wear gloves when handling the bones.

# 4. CAUTIONS:

4.1 All work must be conducted inside the laminar flow cubicle in G8

4.2 Discard all needles, razor blades and sharp instruments in the sharp container provided and close it tightly.

# 5. REAGENTS AND SOLUTIONS:

5.1 NaCl (Merck K91061100)

5.2 KCI (Merck 104936)

(UNILAB 504200)

5.3 MgSO_4_ (Merck 5886)

5.4 Glucose (Merck 8337)

5.5 NaH_2_PO4.2H20 (UNILAB 758516)

5.6 CaCl2 (UNILAB 152490) (BDH 83587)

5.7 NaHCO_3_ (Merck 6329)

**5.8 HEPES (C_8_H_12_N_2_O_4_ ) (UNILAB 18578) repeatation in 5.15**

5.9 KCl (Merck 87009)

5.10 NH_4_Cl (BDH 87925)

5.11 Phosphate Buffered Saline

5.12 Long bones 2x

5.13 Blood in heparin 300ml

5.14 Alcohol (70% ethanol)

5.15 Hepes

5.16 Distilled H_2_O

5.17 Swine Serum

5.18 PBS

5.19 Normal Bovine Serum

5.20 Earle 's 2x concentrate

5.21 Neomycin

5.22 Streptomycin

5.23 Growth medium

5.24 Penicillin

# 6. APPARATUS AND MATERIALS:

6.1 Orbital shaker in a walk-in incubator

6.2 Walk-in incubator set at 37^0^C

6.3 Centrifuge

6.4 CO_2_ Incubator set at 37^0^C

6.5 1 X Scalpel and blade

6.6 1 X Anatomical forceps

6.7 1 X Bone cutter

6.8 Sterile cotton wool

6.9 1 X 500ml Beaker

6.10 1 X 1lt Beaker

6.11 1 X Metal tray

6.12 1pair of Surgical gloves

6.13 1 X 1lt Conical flask

6.14 1 X 100ml Measuring cylinder

6.15 1 X 5ml Glass syringe

6.16 1 X Boat

6.17 4 X Funnels covered with butter muslin cloth

6.18 1 X Micro pipette

6.19 Micro pipette tips

6.20 1 X Multi channel pipette

6.21 10 X Micro titer plates

6.22 2 X 20ml McCartney bottles

6.23 1 X Rubber teat

6.24 Timer/stop watch

# PROCEDURE AND ANALYSIS

Collect bones in a wash buffer and leave at 37^0^C for a period not exceeding 1 hour before use.

7.1 Remove bones from the wash buffer, using forceps, and place them on the metal tray. Using a scalpel blade remove all visible flesh, periosteum and cartilage from the bones.

7.2 Soak bones in 300ml fresh wash buffer in a 500ml beaker and incubate at 37ºC (walk-in incubator) for 20 minutes.

7.3 Crush bones into small fragments using bone cutter, suspend fragments in 250ml wash buffer in 1litre conical flask and shake flask (containing fragments) in orbital shaker inside the walk-in incubator (37^0^C) for 90 minutes.

7.4 Pour contents of the flask through a funnel, covered with butter muslin, divide filtrate to equal amounts in centrifuge containers and centrifuge at 1000 rpm for 15 minutes.

- 1. Discard supernatant, re-suspend pellet in 100ml **ammonium chloride-concentration** and centrifuge at 1000 rpm for 15 minutes.
  2. Discard supernatant, re-suspend pellet in **how much** wash buffer and centrifuge at 1000 rpm for 15 minutes.
  3. Repeat 7.6.
  4. Add growth medium to the cells, mix and pour the mixture into the boat. Using a multi-channel pipette transfer 100μl of cell suspension to each well of micro-titre plate (prepare 10 plates).
  5. Cover each plate with the lid and incubate in a CO2 (5%) incubator at 37ºC for 48 hours.
  6. To wash red blood cells:
     1. Pipette 10ml of blood into each of the two 20ml bottles and add 10ml of PBS to the blood so at to obtain a final volume of 20 ml. Gently mix the PBS and blood.
     2. Centrifuge at 1500 rpm for 10 min then pipette the supernatant and discard.
     3. Add **how much** PBS to the packed red blood cells (from 7.10.2), mix again and centrifuge at 1500 rpm for 10 min. Pipette the supernatant and discard.
     4. Store packed red blood cells in the refrigerator at +4ºC **for how long**.

7.11 After 48 hours of incubation, change growth medium:

7.11.1 Remove cover of micro-plate and flick contents over the (**which basin)** basin.

7.11.2 Add 100 μl of growth medium to each well and cover again.

7.11.3 Incubate plates in a CO2 (5%) incubator at 37ºC for 5 days.

7.11.4 Old cultures can be thrown out at this stage. **Where is this step from?**

# 8. DATA ANALYSIS/CALCULATIONS:

- 1. Analysis of bone marrow cultures macroscopically.

8.1.1 The colour of plates would changes from a light orange to a pale yellow colour in normal cultures (Normally active metabolizing cells produce d acid that turn phenol red to yellow).

- - 1. When cell metabolism ceases in infected cultures, this acid production does not occur. Therefore no colour change is observed as a result of the accumulation of cell breakdown products.
    2. Macroscopic contamination e.g. fungal infection is also checked.

- 1. ANALYSIS OF BONE MARROW CULTURES (MICROSCOPIC)
     1. (Check the size, number and structure or shape).

A fair amount of macrophages indicates or reveals normal cultures. The size of macrophages increases every day and its shape is distinctly round.

- - 1. A decreasing number indicate either a bacterial infection or a toxic effect.

8.2.3 Fungal growth: Long filaments can be seen in the wells. This usually affects the cultures 5-6 days post infection.

8.2.4 Bacterial growth: Seen by poor cell growth. All cells get destroyed. Irregularly shaped aggregations are also visible. These are of no specific shape and tend to have a black dot matrix appearance

# 9. QUALITY CONTROL:

# The EDD R&D laboratory conforms to Good Laboratory practice. For quality control purpose, the required information is documented on the appropriate forms

# REFERENCES:

None

# FORMS AND DATA SHEET:

None
